# The efficacy and adverse events of conventional and second-generation androgen receptor inhibitors for castration-resistant prostate cancer: A network meta-analysis

**DOI:** 10.3389/fendo.2023.1131033

**Published:** 2023-02-10

**Authors:** Xianlu Zhang, Gejun Zhang, Jianfeng Wang, Jianbin Bi

**Affiliations:** Department of Urology Surgery, The First Affiliation Hospital of China Medical University, Shenyang, China

**Keywords:** castration-resistant prostate cancer, network meta-analysis, bicalutamide, enzalutamide, apalutamide, darolutamide

## Abstract

**Background:**

Second-generation androgen receptor inhibitors (ARIs) have been developed and approved for treating castration-resistant prostate cancer (CRPC). There is a lack of direct comparison of the therapeutic effects and adverse events between the conventional ARI (bicalutamide) and three second-generation ARIs (enzalutamide, apalutamide and darolutamide).

**Methods:**

Our network meta-analysis evaluated therapeutic effects and adverse events of the conventional ARI (bicalutamide) and the second-generation ARIs in treating CRPC. We systematically searched the Pubmed, Cochrane library and Embase databases for studies published until October 2022 and only randomized clinical trials (RCTs) were included. The progression-free survival, prostate-specific antigen (PSA) progression-free survival, overall survival (PFS/PSA-PFS/OS), PSA response rate and relative adverse events (AEs) of CRPC patients were collected and synthesized. We then performed subgroup analysis. The non-metastatic and metastatic CRPC (nm/mCRPC) observations were analyzed separately. Data analyses were performed using R software (4.2.1) based on Bayesian framework.

**Results:**

6,993 subjects from seven eligible RCTs were analyzed. Enzalutamide, apalutamide and darolutamide were more effective than bicalutamide in treating CRPC, and the performance of darolutamide was slightly worse than the other two second-generation ARIs. Similar adverse events rate were observed among the second-generation ARIs and bicalutamide. Apalutamide showed a slightly higher rate of Grade 3+ AEs, percentages of AE-related drug withdrawals and AE-related mortality. Patients receiving enzalutamide had significantly higher rate of hypertension and fatigue. In subgroup analysis, enzalutamide showed better therapeutic effects compared with bicalutamide in both nmCRPC and mCRPC groups. In nmCRPC group, enzalutamide and apalutamide had more benefits on PFS and PSA-PFS compared with darolutamide. We displayed the probability ranking map of PFS, PSA-PFS, OS, time to cytotoxic chemotherapy, PSA response rate and relative AE outcomes.

**Conclusion:**

The current network meta-analysis indicated that the second-generation ARIs were superior to the conventional ARI, bicalutamide. The three second-generation ARIs showed incomplete equivalence on CRPC treatment. The darolutamide was slightly less effective compared with enzalutamide and apalutamide. The adverse events of apalutamide were worse than the others, but no statistical significance was observed among these vital AEs. All ARIs were generally well-tolerated. These results may provide reference to clinical decision and further direct comparison trials.

**Systematic review registration:**

https://www.crd.york.ac.uk/PROSPERO, identifier CRD42022370842.

## 1 Introduction

Prostate cancer (PCa) is becoming the second most common cancer in men around the world according to the Global Cancer Statistics 2020. The incidence of prostate cancer is still increasing rapidly especially in China (1). The prostate is a hormone-dependent gland and can be regulated by androgen hormones testosterone and dihydrotestosterone. Due to the androgen dependency, androgen deprivation therapy (ADT) has been used as the first-line treatment for aggressive PCa (2). However, multiple mechanisms of resistance such as androgen receptor (AR) amplification, expression of AR splice variants and AR point mutations contribute to the progression of castration resistant prostate cancer (CRPC) within 2-3 years after adopting ADT (3–5). In recent years, several second-generation androgen receptor inhibitors (ARIs) aiming at reducing the resistance were developed accordingly and were applied as the first-line therapy for CRPC. Studies on the therapeutic effects and adverse events of these second-generation ARIs were carried out. This network meta-analysis indirectly compared and evaluated the therapeutic efficacy and adverse events between conventional ARI (bicalutamide) and second-generation ARIs (enzalutamide, apalutamide and darolutamide).

## 2 Methods

This study was conducted following the PRISMA statement (6) and registered on PROSPERO (Registration No. CRD42022370842) (7). The PRISMA checklist is presented in **Additional file 4**.

### 2.1 Search strategy

PubMed, Embase and Cochrane library were searched for randomized controlled trial (RCTs) from inception to October 1, 2022. The included RCTs should evaluate the therapeutic effects and adverse events (AEs) of the conventional ARI (bicalutamide) and second-generation ARIs in treating CRPC patients. Literature search was conducted by two reviewers independently, and search terms mainly included ‘Prostate cancer’ and ‘Androgen receptor inhibitor’. Detailed search strategy is provided in the Additional file 1.

### 2.2 Inclusion and exclusion criteria

Studies meeting the following criteria were included:

**Types of Participants (P):** Patients diagnosed with CRPC without previous chemotherapy;

**Types of interventions (I):** Patients were grouped randomly and treated with certain ARIs (bicalutamide, enzalutamide, apalutamide, or darolutamide) or placebo. All patients were given ADT background therapy during the whole clinical trial;

**Types of comparisons (C):** The treatment of different ARIs (bicalutamide, enzalutamide, apalutamide, and darolutamide) and placebo;

**Outcome measures (O):** Therapeutic effects included progression-free survival, prostate-specific antigen (PSA) progression-free survival, overall survival (PFS/PSA-PFS/OS), PSA response rate, and metastasis-free survival (MFS). Adverse events included overall AEs, grade 3+ AEs, serious AEs, percentages of AE-related drug withdrawal, AE-related mortality and two representative common AEs (fatigue and hypertension);

**Types of Study(S):** Published randomized controlled trials (RCTs).

Literature review, animal study, conference summary, repeated publication, non-English studies, non-RCT design studies, studies with incomplete data or unavailable studies were excluded.

### 2.3 Data extraction and quality assessment

Data extraction was performed using a pre-designed form, which contained first author, publication date, nation, baseline characteristics of participants (mean age, gender, body mass index, etc.), grouping, and treatment. Risk of Bias (ROB) assessment tool in Cochrane Handbook was applied to assess the quality of included RCTs. Each RCT was graded as ‘high risk’, ‘low risk’ or ‘unclear risk’ according to the following items: random sequence generation, allocation concealment, blinding of participants and research personnel, blinding of outcome assessment, incomplete outcome data, selective reporting, and other bias (8). Data extraction and quality assessment were conducted by two reviewers independently. Disagreements were resolved *via* discussion or by consulting a third reviewer.

### 2.4 Statistical analysis

Data analyses were performed using Gemtc package of R software (version 4.1.2). Markov chain Monte Carlo based on Bayesian framework was applied for modeling. The outcomes used in the analysis including PFS, PSA-PFS, OS, PSA response rate and MFS as well as relative AE data were collected. With regard to PFS and PSA-PFS, subgroup analyses were conducted among nmCRPC and mCRPC patients separately. All the collected data were analyzed under the parameters set up in R (number of chains, 5; tuning iterations, 10,000; simulation iterations, 5,000; thinning interval, 10). Hazard ratio (HR) with 95% confidence interval (95%CI) was used as pooled statistics for dichotomous variables, and mean difference (MD) with 95%CI for continuous data. Heterogeneity test was performed using *I^2^* statistic. The random-effects model was applied if there was significant heterogeneity among included studies (*I^2^* > 50%), otherwise (*I^2^* < 50%), the fixed-effects model was adopted. The original data and R codes are presented in **Additional file 3**.

## 3 Results

### 3.1 Characteristics of the included studies

**Figure 1:**
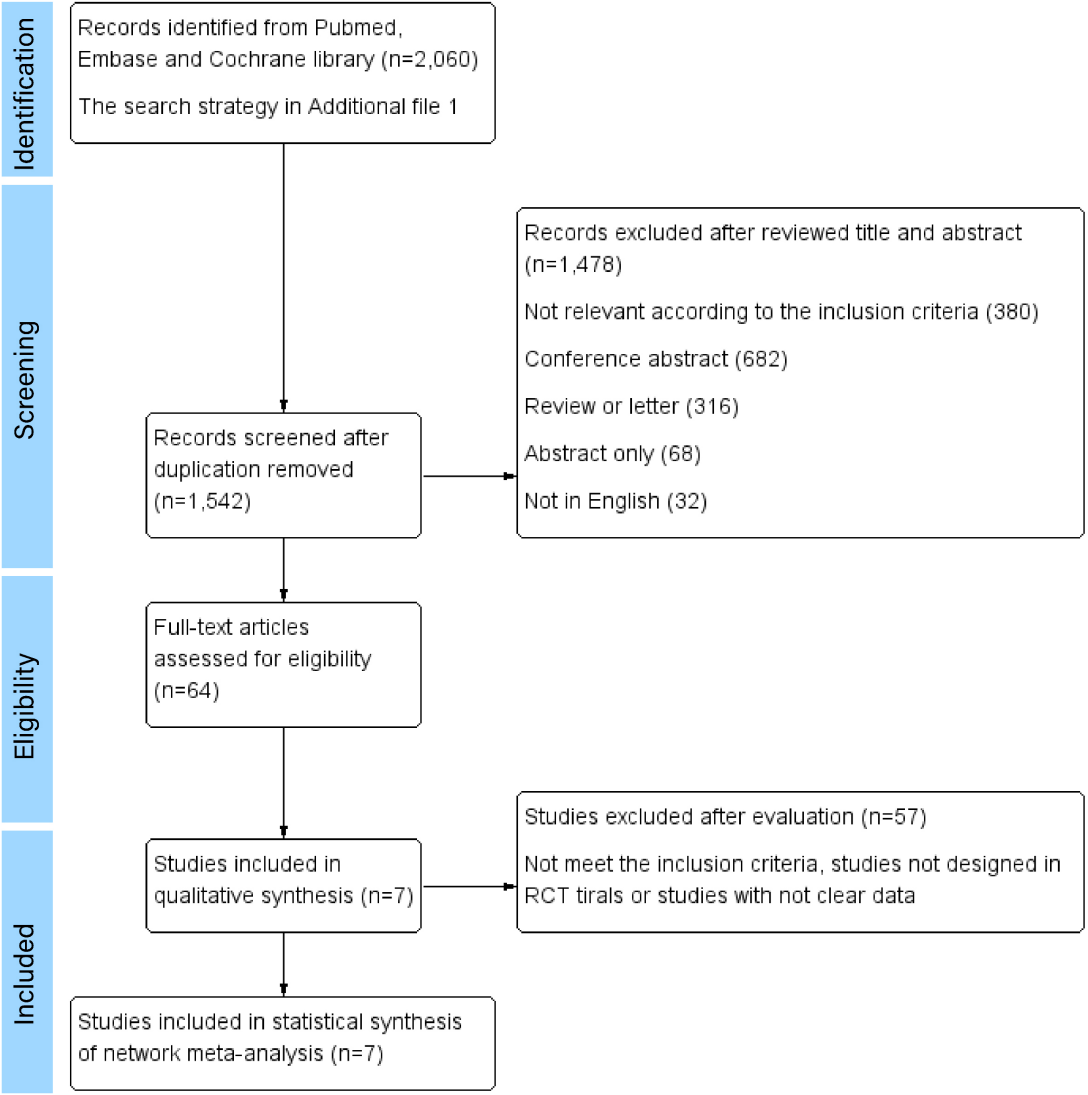
Literature selection process. Seven RCTs were ultimately included in the qualitative and quantitative review for network meta-analyses.

A total of 1,542 articles were initially identified. Among them, 7 eligible studies involving 6,993 participants were synthesized and included in the statistical analysis of this network meta-analysis (9–15). Literature selection process is shown in **Figure 1**.

**Figure 2:**
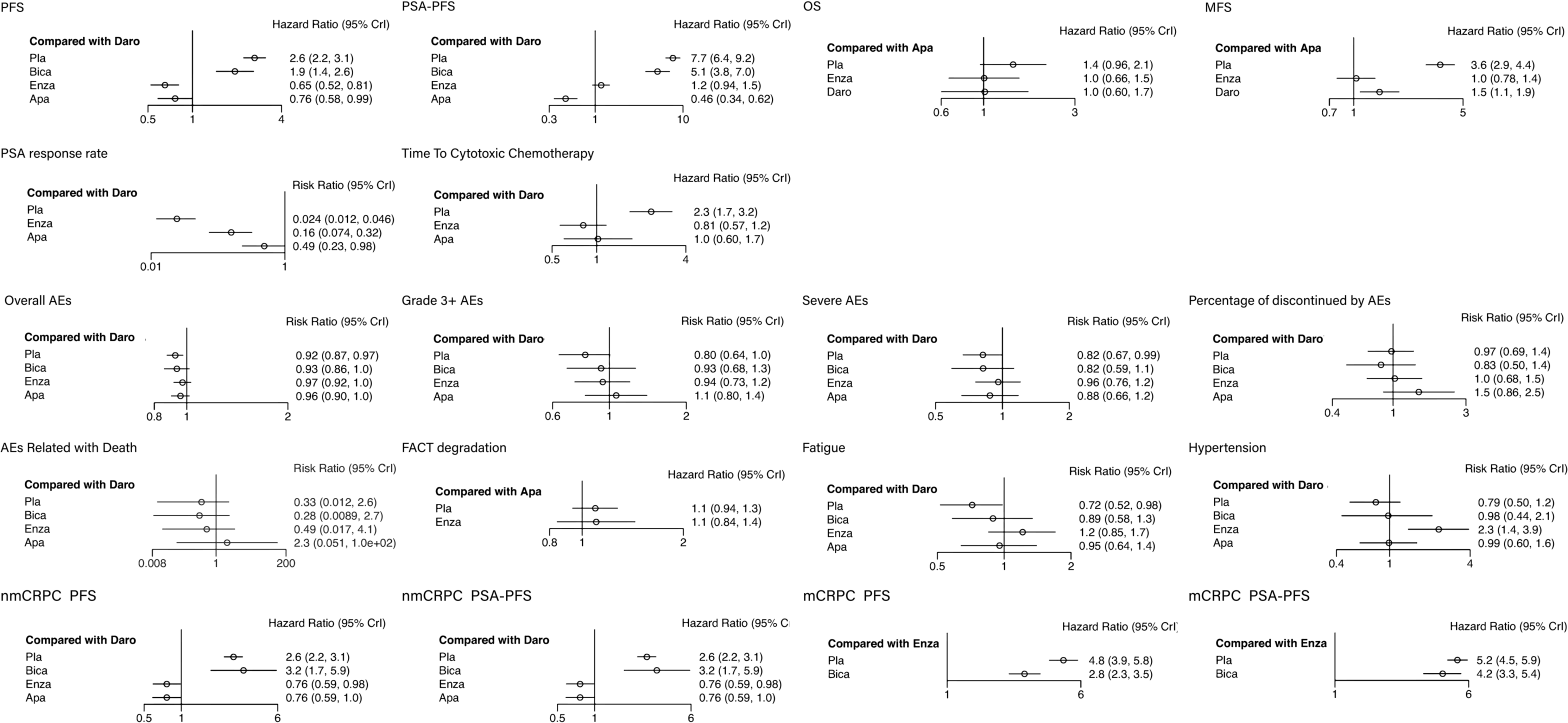
The forest plots of outcomes. The vertical line in the middle is an invalid line, OR=1, indicating that there is no statistical significance between the interventions and the outcomes; The dot is the point estimate of the OR value of each study included; The horizontal line of represents the 95% confidence interval of the OR value.

**Figure 3:**
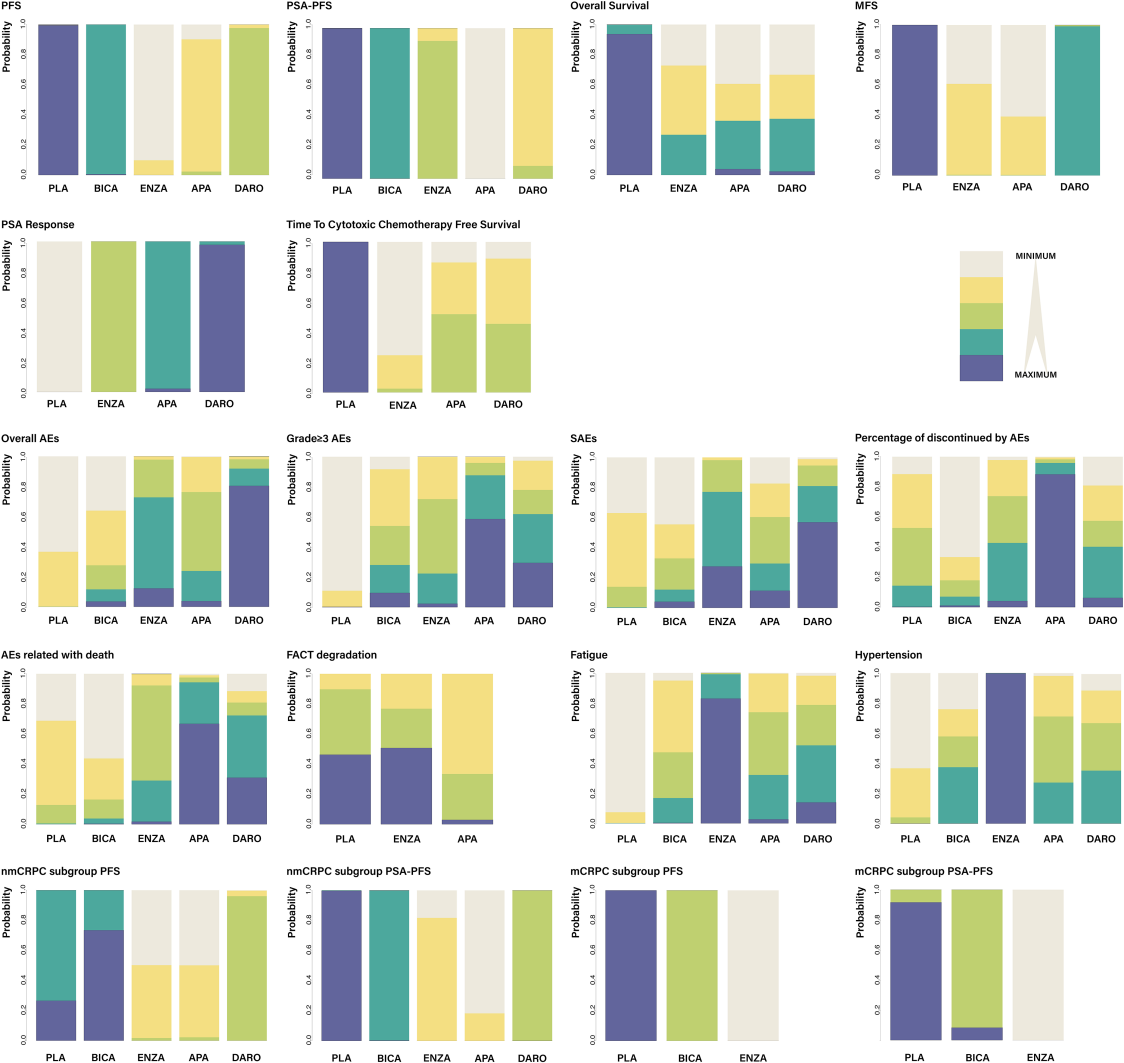
The probability ranking plot of outcomes. The probability ranking plot is the visualized display of rank probability **Supplementary Table** showed in additional file 2. The color depth represents the rank of probabilities.

Among all the included patients, 2,458 patients were treated with placebo, 389 with bicalutamide, 2385 with enzalutamide, 806 with apalutamide and 955 with darolutamide. Characteristics of the included studies are summarized in **Table 1**. Three to five intervention nodes were compared. The network of outcomes is shown in **Supplementary Figure 1** in Additional file 2. The size and edge thickness of each node was weighted according to the number of participants in each comparison. The league table is shown in **Supplementary Table 1** in Additional file 2. The forest plots of outcomes are shown in **Figure 2**. The rank probability table is displayed in **Supplementary Table 2** in Additional file 2. The probability ranking plot of outcomes is shown in **Figure 3**. Results of risk of bias assessment are presented in **Supplementary Figure 2** in Additional file 2.

**Table:** Table 1 Characteristics of included studies.

Study	Year	Country	Patients	Sample size of each arm				
				PLA (n = 2458)	BICA (n = 389)	ENZA (n = 2385)	APA (n = 806)	DARO (n = 955)
Beer et al.	2014	USA	mCRPC	845	–	872	–	–
Fizazi et al.	2019	France	nmCRPC	554	–	–	–	955
Hussain et al.	2018	USA	nmCRPC	468	–	933	–	–
Penson et al.	2016	USA	CRPC	–	198	198	–	–
Pu et al.	2022	China	mCRPC	190	–	198	–	–
Shore et al.	2016	USA	mCRPC	–	191	184	–	–
Smith et al.	2018	USA	nmCRPC	401	–	–	806	–

PLA stands for placebo; BICA stands for bicalutamide; ENZA stands for enzalutamide; APA stands for apalutamide; DARO stands for darolutamide.

### 3.2 Network meta-analysis of therapeutic efficacy

#### 3.2.1 PFS

Meta-analysis showed that compared with placebo, bicalutamide (HR: 0.74, 95% CI: 0.58–0.93) improved the PFS, while enzalutamide, apalutamide and darolutamide (HR: 0.25, 95% CI: 0.22–0.28; HR: 0.29, 95% CI: 0.24–0.36; HR: 0.38, 95% CI: 0.32–0.45) significantly improved the PFS. Among the second-generation ARIs, the enzalutamide and apalutamide (HR: 1.54, 95% CI: 1.24–1.91; HR: 1.31, 95% CI: 1.01–1.71) showed more benefits compared with darolutamide.

#### 3.2.2 PSA-PFS

Meta-analysis showed that compared with placebo, bicalutamide (HR: 0.67, 95% CI: 0.52–0.86) improved PSA-PFS, while enzalutamide, apalutamide and darolutamide (HR: 0.15, 95% CI: 0.14–0.17; HR: 0.06, 95% CI: 0.05–0.08; HR: 0.13, 95% CI: 0.11–0.16) significantly improved PSA-PFS. Among the second-generation ARIs, apalutamide (HR: 2.17, 95% CI: 1.61–2.93) showed more benefits compared with enzalutamide and darolutamide.

#### 3.2.3 OS

The OS in bicalutamide group was not included in the analysis. Enzalutamide, apalutamide and darolutamide (HR: 0.71, 95% CI: 0.61–0.82; HR: 0.70, 95% CI: 0.47–1.04; HR: 0.71, 95% CI: 0.51–1.00) showed similar effects in improving the OS.

#### 3.2.4 MFS

The MFS in bicalutamide group was not included in the analysis. Enzalutamide, apalutamide and darolutamide (HR: 0.29, 95% CI: 0.24–0.35; HR: 0.28, 95% CI: 0.23–0.24; HR: 0.41, 95% CI: 0.34–0.50) significantly elevated MFS. Besides, enzalutamide and apalutamide showed better performance in increasing MFS compared with darolutamide (HR: 1.41, 95% CI: 1.08–1.85; HR: 1.47, 95% CI: 1.10–1.95).

#### 3.2.5 PSA response rate

The PSA response rate in bicalutamide group was not included in the analysis. Enzalutamide, apalutamide and darolutamide (HR: 6.44, 95% CI: 4.74–9.04; HR: 20.11 95% CI: 15.69–27.04; HR: 41.17, 95% CI: 21.86–82.94) significantly improved PSA response. Darolutamide showed better performance in improving PSA response compared withenzalutamide and apalutamide (HR: 0.16, 95% CI: 0.07–0.32; HR: 0.49, 95% CI: 0.23–0.98).

#### 3.2.6 Time to cytotoxic chemotherapy free survival

The time to cytotoxic chemotherapy free survival in bicalutamide group was not included in the analysis. Enzalutamide, apalutamide and darolutamide (HR: 0.35, 95% CI: 0.30–0.40; HR: 0.44, 95% CI: 0.29–0.66; HR: 0.43, 95% CI: 0.31–0.60) significantly prolonged the time to cytotoxic chemotherapy free survival. Enzalutamide showed better effects compared with apalutamide and darolutamide, however, the difference was not statistically significant. (HR: 1.26, 95% CI: 0.81–1.95; HR: 1.23, 95% CI: 0.86–1.77).

### 3.3 Network meta-analysis of adverse effects

#### 3.3.1 Overall AEs

The overall AEs in bicalutamide group was not included in the analysis. Enzalutamide, apalutamide and darolutamide (HR: 1.42, 95% CI: 1.22–1.64; HR: 1.43, 95% CI: 0.96–2.12; HR: 1.41, 95% CI: 1.00–1.98) increased the overall AE rate, and there was no significant difference among the three agents.

#### 3.3.2 Grade 3+ AEs

Meta-analysis showed that compared with placebo, all ARIs increased the rate of grade3+ AEs, while there was no significant difference between bicalutamide and darolutamide groups (HR: 1.16, 95% CI: 0.94–1.42; HR: 1.24, 95% CI: 1.00–1.57). Enzalutamide and apalutamide (HR: 1.17, 95% CI: 1.07–1.29; HR: 1.32, 95% CI: 1.13–1.55) slightly increased AEs rate with statistically significant difference.

#### 3.3.3 SAE

Meta-analysis showed that compared with placebo, bicalutamide and apalutamide did not lead to SAEs (HR: 1.00, 95% CI: 0.78–1.29; HR: 1.07, 95% CI: 0.87–1.34). Enzalutamide and darolutamide slightly elevated SAE risk (HR: 1.17, 95% CI: 1.04–1.32; HR: 1.22, 95% CI: 1.01–1.50).

#### 3.3.4 Percentage of discontinued by AEs

Meta-analysis showed that compared with placebo, bicalutamide, enzalutamide, and darolutamide (HR: 0.86, 95% CI: 0.58–1.28; HR: 1.05, 95% CI: 0.84–1.33; HR: 1.03, 95% CI: 0.74–1.46) did not reduce the risk of AE-related drug withdrawal, while apalutamide elevated the risk (HR: 1.51, 95% CI: 1.02–2.32).

#### 3.3.5 AE-related mortality

Meta-analysis showed that there was no statistical difference in AE-related mortality between placebo and bicalutamide as well as darolutamide (HR: 0.86, 95% CI: 0.34–2.11; HR: 3.03, 95% CI: 0.38–85.13). Apalutamide and enzalutamide (HR: 6.73, 95% CI: 1.1–183.14; HR: 1.49, 95% CI: 1.03–2.20) increased the risk of AE-related mortality with statistical significance compared with placebo, and the risk in apalutamide group was the highest.

#### 3.3.6 FACT degradation

Only the data in placebo, enzalutamide and apalutamide groups were analyzed for this outcome. There was no significant difference between placebo and enzalutamide as well as apalutamide (HR: 1.01, 95% CI: 0.74–1.37; HR: 0.91, 95% CI: 0.79–1.07).

#### 3.3.7 Fatigue

Meta-analysis showed that compared with placebo, bicalutamide (HR: 1.24, 95% CI: 0.96–1.61) did not reduce fatigue risk. Enzalutamide, apalutamide and darolutamide (HR: 1.69, 95% CI: 1.50–1.90; HR: 1.33, 95% CI: 1.07–1.66; HR: 1.39, 95% CI: 1.02–1.94) increased the risk of fatigue compared with placebo, and there was no statistical difference among them.

#### 3.3.8 Hypertension

Meta-analysis showed that compared with placebo, bicalutamide and darolutamide (HR: 1.16, 95% CI: 0.94–1.42; HR: 1.24, 95% CI: 1.00–1.57) did not reduce hypertension risk. Enzalutamide and apalutamide (HR: 1.17, 95% CI: 1.07–1.29; HR: 1.32, 95% CI: 1.13–1.55) slightly increased the risk of hypertension compared with placebo.

#### 3.3.9 Subgroup analysis

All five interventions were evaluated for nmCRPC group. Only placebo, bicalutamide and enzalutamide were evaluated for mCRPC subgroup. Two major oncology outcomes, PFS and PSA-PFS, were analyzed.

#### 3.3.10 nmCRPC

Meta-analysis showed that compared with placebo, bicalutamide (HR: 1.20, 95% CI: 0.68–2.18) had no benefits in improving PFS. Enzalutamide, apalutamide and darolutamide (HR: 0.29, 95% CI: 0.24–0.35; HR: 0.29, 95% CI: 0.24–0.36; HR: 0.38, 95% CI: 0.32–0.45) significantly improved PFS. Moreover, enzalutamide and darolutamide (HR: 1.31, 95% CI: 1.02–1.69; HR: 1.31, 95% CI: 1.00–1.71) showed better effects compared with darolutamide in nmCRPC patients.

For PSA-PFS, bicalutamide, enzalutamide, apalutamide and darolutamide (HR: 0.39, 95% CI: 0.20–0.75; HR: 0.07, 95% CI: 0.06–0.09; HR: 0.06, 95% CI: 0.05–0.08; HR: 0.13, 95% CI: 0.11–0.16) were all beneficial for nmCRPC patients. The second-generation ARIs had a significant elevation in PSA-PFS compared with bicalutamide. Similar as PFS, there was no significant difference in increasing PSA-PFS between darolutamide and enzalutamide as well as apalutamide (HR: 1.86, 95% CI: 1.38–2.50; HR: 2.17, 95% CI: 1.61–2.93).

#### 3.3.11 mCRPC

Meta-analysis showed that compared with placebo, bicalutamide and enzalutamide (HR: 0.59, 95% CI: 0.45–0.78; HR: 0.21, 95% CI: 0.17–0.25) both improved PFS and enzalutamide showed better effects compared with bicalutamide. For PSA-PFS, bicalutamide did not show significantly better therapeutic effect (HR: 0.82, 95% CI: 0.61–1.08), while enzalutamide (HR:0.19, 95% CI: 0.17–0.22) greatly improved PSA-PFS.

## 4 Discussion

We performed a network meta-analysis comparing the conventional ARI (bicalutamide) and second-generation ARIs (enzalutamide, apalutamide and darolutamide). The conventional ARIs, represented by bicalutamide and flutamide, prevent downstream signaling following AR translocation to the nucleus. They have equivalent anti-prostate cancer activity compared with castration and are well-tolerated by patients (16). However, as the therapy time prolongs, there is a growing risk of drug resistance to conventional ARIs. Besides, a previous study found that the bicalutamide might acquire agonistic properties during long-term ADT (17). For patients at CRPC stage, the second-generation ARIs might be a better option. The second-generation antiandrogens could be divided into androgen biosynthesis inhibitor (Abiraterone Acetate) targeting at the upstream of androgen synthesis, and ARIs (enzalutamide, apalutamide and daroludamide) targeting at the downstream of AR signaling. In this study, we evaluated the oncology-related outcomes and adverse events of the conventional and second-generation ARIs. According to the inclusion criteria, only pre-chemotherapy patients with CRPC receiving ADT background treatment were included. We demonstrated that the second-generation ARIs showed significant benefits for CRPC patients compared with conventional ARI. However, the equivalence among three second-generation ARIs was not precise.

For all the CRPC patients, the therapeutic effect of bicalutamide was not remarkable. Enzalutamide, apalutamide and darolutamide all significantly improved PFS and PSA-PFS compared with bicalutamide. Among the three second-generation ARIs, enzalutamide and apalutamide showed slightly better effects on improving the three vital therapeutic indicators (PFS, PSA-PFS and MFS) compared with darolutamide. In the pre-clinical study, darolutamide had a remarkably lower inhibition constant value compared with enzalutamide in competitive AR binding assays. This indicated that darolutamide had a higher inhibition capacity, while the inhibition effects on nuclear translocation of AR between darolutamide and enzalutamide was similar (18). The results of this pre-clinical study seemed inconsistent with the results of indirect comparison between enzalutamide and darolutamide. This might be attributed to the fact that the enzalutamide group included both nmCRPC and mCRPC patients while the darolutamide group included only nmCRPC patients. When the participants were restricted into nmCRPC patients only, compared with darolutamide, the relative risk of PFS in enzalutamide group was higher than that in the overall group (RR=0.76, 0.59-0.98 versus RR=0.65, 0.52-0.81). In nmCRPC group, patients with pelvic lymph nodes of less than 2cm in diameter in short axis below the aortic bifurcation were included in darolutamide group in ARAMIS trial while excluded in enzalutamide group in PROSPER trial, which might also affect the results of the comparison.

The meta-analysis showed that PSA-PFS preformed best in apalutamide group compared with other second-generation ARIs. Apalutamide could retain full antagonist-activity in the setting of increased AR expression (19). The affinity of apalutamide was significantly stronger than that of bicalutamide *in vitro*. PFS and MFS results in apalutamide group were similar as enzalutamide group. Only nmCRPC patients were included in apalutamide group, and this might also contribute to the slightly better PSA-PFS in apalutamide group compared with enzalutamide group. After the bias was adjusted, in the nmCRPC subgroup, apalutamide showed similar therapeutic effects on PFS and PSA-PFS as enzalutamide.

OS and MFS were not evaluated in the studies on bicalutamide. Previous study suggested that the first-generation ARIs (bicalutamide or flutamide) or estrogens could not improve OS (20). In mCRPC subgroup, our results also suggested that bicalutamide basically had no benefit in PFS and PSA-PFS. The meta-analysis revealed that the three second-generation ARIs had similar benefit in the OS. The aim of treating nmCRPC is to prolong the time to metastasis, which are manifested by pain, pathological fracture or nerve root compression (21). Darolutamide showed weak effect on improving MFS compared with enzalutamide and apalutamide. MFS was evaluated only in patients with nmCRPC. This network meta-analysis reported the equivalence of second-generation ARIs. Furthermore, darolutamide was found to perform slightly worse in three main measurements (PFS, PSA-PFS and MFS), and relative equivalence was observed in enzalutamide and apalutamide.

Previous meta-analysis concluded that darolutamide had the lowest incidence of grade 3+ AEs and toxicity leading to drug discontinuation compared with other agents, suggesting that darolutamide was more well-tolerated by patients (22, 23). In previous studies, only patients with nmCRPC were included in enzalutamide and apalutamide groups. Our study also included mCRPC patients in these two groups and distinct outcomes were observed. For the overall AEs, we found that all ARIs increased AE risk and darolutamide showed a higher tendency, although there was no statistically significant difference among the four interventions. The second-generation ARIs all had higher SAE incidence compared with bicalutamide. Notably, the incidence of grade 3+ AEs, AE-related drug withdrawal, and AE-related mortality were relatively the highest in apalutamide group, although there was no statistically significant difference among these ARIs. Reducing the risk of seizures stemming induced by γ-aminobutyric acid receptor inhibition was emphasized during development of apalutamide. Besides, the preclinical study found only a small amount of darolutamide could penetrate through the blood–brain barrier, which led to theoretically reduction of central nervous system related AEs (24). Enzalutamide had the highest incidence of the most representative and common AEs (fatigue and hypertension) among all interventions. There was a statistically significant increase in the incidence of hypertension in the enzalutamide group. As the first acknowledged second-generation antiandrogen, enzalutamide is generally well-tolerated and the risk of AEs is basically under control.

To our knowledge, our study is the first network meta-analysis comprehensively comparing the conventional and second generation ARIs. Previous meta-analysis only included nmCRPC patients in AEs evaluation, and we also included mCRPC patients to acquire more reliable results. There were also some limitations of this network meta-analysis. Although the studies we included were generally rated as low risk, the quality of our outcomes was still influenced by unclear bias risk of the original reports, such as the outcome bias. The follow-up time was not sufficient in which the overall survival was not observed in most studies. Therefore, the follow-up time in further studies should be prolonged to acquire more reliable results. Besides, because of the ‘ring’ of interventions could not be established, the consistency failed to be test.

## 5 Conclusion

This network meta-analysis revealed that the second-generation ARIs were statistically superior to the conventional ARI (bicalutamide). Among the second generation ARIs enzalutamide, apalutamide and darolutamide, the therapeutic effects were not completely equivalent. Enzalutamide and apalutamide had similar therapeutic effects while darolutamide seemed to be slightly less beneficial. Although darolutamide had more overall AEs, apalutamide resulted in higher incidence of three vital AEs. No statistically significant difference in these vital AEs was observed among these ARIs. All ARIs are generally well-tolerated. This network meta-analysis indirectly compared the second-generation ARIs with placebo. Direct comparison study is required to supplement the results of indirect comparison study.

## Data availability statement

The original contributions presented in the study are included in the article/supplementary material. Further inquiries can be directed to the corresponding author.

## Author contributions

XZ: Protocol/project development, Data management, Data analysis, Manuscript writing/editing; GZ: Data management, Data collection, Manuscript writing/editing; JW: Data collection, Manuscript writing/editing; JB: Protocol/project development, Data analysis, Manuscript writing/editing. All authors contributed to the article and approved the submitted version.
