# Supplementary material for: The efficacy and adverse events of conventional and second-generation androgen receptor inhibitors for castration-resistant prostate cancer: A network meta-analysis
Source: Front Endocrinol (Lausanne). 2023 Feb 10;14:1131033. doi: 10.3389/fendo.2023.1131033 (PMC9950258; doi:10.3389/fendo.2023.1131033)
Supplement: Supplementary file 1 [file Table_1.docx]

Supplementary Material

The efficacy and adverse events of conventional and second-generation androgen receptor inhibitors for castration-resistant prostate cancer: a network meta-analysis

Xianlu Zhang^1^, Gejun Zhang^1^, Jianfeng Wang^1^, Jianbin Bi^1*^

*** Correspondence:** Jianbin Bi: jianbinbi@cmu.edu.cn

# Table S1 Search strategy

| Pubmed | | | | |
| --- | --- | --- | --- | --- |
| Search number | Query | Search details | Results | Time |
|  |  |  |  |  |
| 9 | #7 AND #8 | ("prostatic neoplasms, castration resistant"[MeSH Terms] OR ((("castration resistant prostatic neoplasm"[Title/Abstract] OR (("neoplasm s"[All Fields] OR "Neoplasms"[MeSH Terms] OR "Neoplasms"[All Fields] OR "neoplasm"[All Fields]) AND "castration resistant prostatic"[Title/Abstract]) OR (("neoplasm s"[All Fields] OR "Neoplasms"[MeSH Terms] OR "Neoplasms"[All Fields] OR "neoplasm"[All Fields]) AND "castration resistant prostatic"[Title/Abstract]) OR (("prostatic neoplasms"[MeSH Terms] OR ("Prostatic"[All Fields] AND "Neoplasms"[All Fields]) OR "prostatic neoplasms"[All Fields] OR ("Prostatic"[All Fields] AND "neoplasm"[All Fields]) OR "prostatic neoplasm"[All Fields]) AND "Castration-Resistant"[Title/Abstract]) OR "prostatic neoplasms castration resistant"[Title/Abstract] OR ("Androgen-Insensitive"[All Fields] AND "prostatic neoplasms"[Title/Abstract]) OR ((("androgen s"[All Fields] OR "androgene"[All Fields] OR "androgenes"[All Fields] OR "androgenic"[All Fields] OR "androgenicity"[All Fields] OR "androgenized"[All Fields] OR "androgenizing"[All Fields] OR "androgenous"[All Fields] OR "androgens"[Pharmacological Action] OR "androgens"[MeSH Terms] OR "androgens"[All Fields] OR "Androgen"[All Fields] OR "virilism"[MeSH Terms] OR "virilism"[All Fields] OR "androgenization"[All Fields]) AND ("Insensitive"[All Fields] OR "insensitivities"[All Fields] OR "insensitivity"[All Fields])) AND "prostatic neoplasms"[Title/Abstract]) OR ("Androgen-Resistant"[All Fields] AND "prostatic neoplasms"[Title/Abstract]) OR (("androgen s"[All Fields] OR "androgene"[All Fields] OR "androgenes"[All Fields] OR "androgenic"[All Fields] OR "androgenicity"[All Fields] OR "androgenized"[All Fields] OR "androgenizing"[All Fields] OR "androgenous"[All Fields] OR "androgens"[Pharmacological Action] OR "androgens"[MeSH Terms] OR "androgens"[All Fields] OR "Androgen"[All Fields] OR "virilism"[MeSH Terms] OR "virilism"[All Fields] OR "androgenization"[All Fields]) AND "resistant prostatic neoplasms"[Title/Abstract]) OR (("prostatic neoplasms"[MeSH Terms] OR ("Prostatic"[All Fields] AND "Neoplasms"[All Fields]) OR "prostatic neoplasms"[All Fields]) AND "hormone refractory"[Title/Abstract]) OR ((("hormon"[All Fields] OR "hormonal"[All Fields] OR "hormonally"[All Fields] OR "hormonals"[All Fields] OR "hormone s"[All Fields] OR "hormones"[Pharmacological Action] OR "hormones"[MeSH Terms] OR "hormones"[All Fields] OR "Hormone"[All Fields] OR "hormons"[All Fields]) AND ("refractories"[All Fields] OR "refractoriness"[All Fields] OR "Refractory"[All Fields])) AND "prostatic neoplasms"[Title/Abstract]) OR (("prostatic neoplasms"[MeSH Terms] OR ("Prostatic"[All Fields] AND "Neoplasms"[All Fields]) OR "prostatic neoplasms"[All Fields]) AND "Androgen-Independent"[Title/Abstract]) OR ("Androgen-Independent"[All Fields] AND "prostatic neoplasm"[Title/Abstract]) OR (("neoplasm s"[All Fields] OR "Neoplasms"[MeSH Terms] OR "Neoplasms"[All Fields] OR "neoplasm"[All Fields]) AND "androgen independent prostatic"[Title/Abstract]) OR (("neoplasm s"[All Fields] OR "Neoplasms"[MeSH Terms] OR "Neoplasms"[All Fields] OR "neoplasm"[All Fields]) AND "androgen independent prostatic"[Title/Abstract]) OR (("prostatic neoplasms"[MeSH Terms] OR ("Prostatic"[All Fields] AND "Neoplasms"[All Fields]) OR "prostatic neoplasms"[All Fields] OR ("Prostatic"[All Fields] AND "neoplasm"[All Fields]) OR "prostatic neoplasm"[All Fields]) AND "Androgen-Independent"[Title/Abstract]) OR (("prostatic neoplasms"[MeSH Terms] OR ("Prostatic"[All Fields] AND "Neoplasms"[All Fields]) OR "prostatic neoplasms"[All Fields]) AND "Androgen-Independent"[Title/Abstract]) OR (("prostatic neoplasms"[MeSH Terms] OR ("Prostatic"[All Fields] AND "Neoplasms"[All Fields]) OR "prostatic neoplasms"[All Fields]) AND "Androgen-Insensitive"[Title/Abstract]) OR ("Androgen-Insensitive"[All Fields] AND "prostatic neoplasm"[Title/Abstract]) OR (("neoplasm s"[All Fields] OR "Neoplasms"[MeSH Terms] OR "Neoplasms"[All Fields] OR "neoplasm"[All Fields]) AND "androgen insensitive prostatic"[Title/Abstract]) OR (("neoplasm s"[All Fields] OR "Neoplasms"[MeSH Terms] OR "Neoplasms"[All Fields] OR "neoplasm"[All Fields]) AND "androgen insensitive prostatic"[Title/Abstract]) OR (("prostatic neoplasms"[MeSH Terms] OR ("Prostatic"[All Fields] AND "Neoplasms"[All Fields]) OR "prostatic neoplasms"[All Fields] OR ("Prostatic"[All Fields] AND "neoplasm"[All Fields]) OR "prostatic neoplasm"[All Fields]) AND "Androgen-Insensitive"[Title/Abstract]) OR (("prostatic neoplasms"[MeSH Terms] OR ("Prostatic"[All Fields] AND "Neoplasms"[All Fields]) OR "prostatic neoplasms"[All Fields]) AND "Androgen-Insensitive"[Title/Abstract]) OR (("prostatic neoplasms"[MeSH Terms] OR ("Prostatic"[All Fields] AND "Neoplasms"[All Fields]) OR "prostatic neoplasms"[All Fields]) AND "Androgen-Resistant"[Title/Abstract]) OR ("Androgen-Resistant"[All Fields] AND "prostatic neoplasm"[Title/Abstract]) OR (("neoplasm s"[All Fields] OR "Neoplasms"[MeSH Terms] OR "Neoplasms"[All Fields] OR "neoplasm"[All Fields]) AND "androgen resistant prostatic"[Title/Abstract]) OR (("neoplasm s"[All Fields] OR "Neoplasms"[MeSH Terms] OR "Neoplasms"[All Fields] OR "neoplasm"[All Fields]) AND "androgen resistant prostatic"[Title/Abstract]) OR (("prostatic neoplasms"[MeSH Terms] OR ("Prostatic"[All Fields] AND "Neoplasms"[All Fields]) OR "prostatic neoplasms"[All Fields] OR ("Prostatic"[All Fields] AND "neoplasm"[All Fields]) OR "prostatic neoplasm"[All Fields]) AND "Androgen-Resistant"[Title/Abstract]) OR (("prostatic neoplasms"[MeSH Terms] OR ("Prostatic"[All Fields] AND "Neoplasms"[All Fields]) OR "prostatic neoplasms"[All Fields]) AND "Androgen-Resistant"[Title/Abstract]) OR "androgen independent prostatic neoplasms"[Title/Abstract] OR "androgen independent prostatic neoplasms"[Title/Abstract] OR "castration resistant prostatic neoplasms"[Title/Abstract] OR "castration resistant prostatic neoplasms"[Title/Abstract] OR "prostatic cancer castration resistant"[Title/Abstract] OR (("cancer s"[All Fields] OR "cancerated"[All Fields] OR "canceration"[All Fields] OR "cancerization"[All Fields] OR "cancerized"[All Fields] OR "cancerous"[All Fields] OR "Neoplasms"[MeSH Terms] OR "Neoplasms"[All Fields] OR "Cancer"[All Fields] OR "Cancers"[All Fields]) AND "castration resistant prostatic"[Title/Abstract]) OR (("cancer s"[All Fields] OR "cancerated"[All Fields] OR "canceration"[All Fields] OR "cancerization"[All Fields] OR "cancerized"[All Fields] OR "cancerous"[All Fields] OR "Neoplasms"[MeSH Terms] OR "Neoplasms"[All Fields] OR "Cancer"[All Fields] OR "Cancers"[All Fields]) AND "castration resistant prostatic"[Title/Abstract]) OR ("Castration-Resistant"[All Fields] AND "prostatic cancers"[Title/Abstract]) OR "prostatic cancer castration resistant"[Title/Abstract] OR (("prostatic neoplasms"[MeSH Terms] OR ("Prostatic"[All Fields] AND "Neoplasms"[All Fields]) OR "prostatic neoplasms"[All Fields] OR ("Prostatic"[All Fields] AND "Cancers"[All Fields]) OR "prostatic cancers"[All Fields]) AND "Castration-Resistant"[Title/Abstract]) OR "androgen insensitive prostatic cancer"[Title/Abstract] OR "androgen insensitive prostatic cancer"[Title/Abstract] OR "androgen resistant prostatic cancer"[Title/Abstract] OR "androgen resistant prostatic cancer"[Title/Abstract] OR (("prostatic neoplasms"[MeSH Terms] OR ("Prostatic"[All Fields] AND "Neoplasms"[All Fields]) OR "prostatic neoplasms"[All Fields] OR ("Prostatic"[All Fields] AND "Cancer"[All Fields]) OR "prostatic cancer"[All Fields]) AND "hormone refractory"[Title/Abstract]) OR "hormone refractory prostatic cancer"[Title/Abstract] OR (("prostat"[All Fields] OR "prostate"[MeSH Terms] OR "prostate"[All Fields] OR "prostates"[All Fields] OR "Prostatic"[All Fields] OR "prostatism"[MeSH Terms] OR "prostatism"[All Fields] OR "prostatitis"[MeSH Terms] OR "prostatitis"[All Fields]) AND "cancer androgen independent"[Title/Abstract]) OR "androgen independent prostatic cancers"[Title/Abstract] OR (("cancer s"[All Fields] OR "cancerated"[All Fields] OR "canceration"[All Fields] OR "cancerization"[All Fields] OR "cancerized"[All Fields] OR "cancerous"[All Fields] OR "Neoplasms"[MeSH Terms] OR "Neoplasms"[All Fields] OR "Cancer"[All Fields] OR "Cancers"[All Fields]) AND "androgen independent prostatic"[Title/Abstract]) OR (("cancer s"[All Fields] OR "cancerated"[All Fields] OR "canceration"[All Fields] OR "cancerization"[All Fields] OR "cancerized"[All Fields] OR "cancerous"[All Fields] OR "Neoplasms"[MeSH Terms] OR "Neoplasms"[All Fields] OR "Cancer"[All Fields] OR "Cancers"[All Fields]) AND "androgen independent prostatic"[Title/Abstract]) OR (("prostat"[All Fields] OR "prostate"[MeSH Terms] OR "prostate"[All Fields] OR "prostates"[All Fields] OR "Prostatic"[All Fields] OR "prostatism"[MeSH Terms] OR "prostatism"[All Fields] OR "prostatitis"[MeSH Terms] OR "prostatitis"[All Fields]) AND "cancer androgen independent"[Title/Abstract]) OR (("prostat"[All Fields] OR "prostate"[MeSH Terms] OR "prostate"[All Fields] OR "prostates"[All Fields] OR "Prostatic"[All Fields] OR "prostatism"[MeSH Terms] OR "prostatism"[All Fields] OR "prostatitis"[MeSH Terms] OR "prostatitis"[All Fields]) AND "cancers androgen independent"[Title/Abstract]) OR (("prostat"[All Fields] OR "prostate"[MeSH Terms] OR "prostate"[All Fields] OR "prostates"[All Fields] OR "Prostatic"[All Fields] OR "prostatism"[MeSH Terms] OR "prostatism"[All Fields] OR "prostatitis"[MeSH Terms] OR "prostatitis"[All Fields]) AND "cancer androgen insensitive"[Title/Abstract]) OR ("Androgen-Insensitive"[All Fields] AND "prostatic cancers"[Title/Abstract]) OR (("cancer s"[All Fields] OR "cancerated"[All Fields] OR "canceration"[All Fields] OR "cancerization"[All Fields] OR "cancerized"[All Fields] OR "cancerous"[All Fields] OR "Neoplasms"[MeSH Terms] OR "Neoplasms"[All Fields] OR "Cancer"[All Fields] OR "Cancers"[All Fields]) AND "androgen insensitive prostatic"[Title/Abstract]) OR (("cancer s"[All Fields] OR "cancerated"[All Fields] OR "canceration"[All Fields] OR "cancerization"[All Fields] OR "cancerized"[All Fields] OR "cancerous"[All Fields] OR "Neoplasms"[MeSH Terms] OR "Neoplasms"[All Fields] OR "Cancer"[All Fields] OR "Cancers"[All Fields]) AND "androgen insensitive prostatic"[Title/Abstract]) OR (("prostat"[All Fields] OR "prostate"[MeSH Terms] OR "prostate"[All Fields] OR "prostates"[All Fields] OR "Prostatic"[All Fields] OR "prostatism"[MeSH Terms] OR "prostatism"[All Fields] OR "prostatitis"[MeSH Terms] OR "prostatitis"[All Fields]) AND "cancer androgen insensitive"[Title/Abstract]) OR (("prostatic neoplasms"[MeSH Terms] OR ("Prostatic"[All Fields] AND "Neoplasms"[All Fields]) OR "prostatic neoplasms"[All Fields] OR ("Prostatic"[All Fields] AND "Cancers"[All Fields]) OR "prostatic cancers"[All Fields]) AND "Androgen-Insensitive"[Title/Abstract]) OR (("prostat"[All Fields] OR "prostate"[MeSH Terms] OR "prostate"[All Fields] OR "prostates"[All Fields] OR "Prostatic"[All Fields] OR "prostatism"[MeSH Terms] OR "prostatism"[All Fields] OR "prostatitis"[MeSH Terms] OR "prostatitis"[All Fields]) AND "cancer androgen resistant"[Title/Abstract]) OR ("Androgen-Resistant"[All Fields] AND "prostatic cancers"[Title/Abstract]) OR (("cancer s"[All Fields] OR "cancerated"[All Fields] OR "canceration"[All Fields] OR "cancerization"[All Fields] OR "cancerized"[All Fields] OR "cancerous"[All Fields] OR "Neoplasms"[MeSH Terms] OR "Neoplasms"[All Fields] OR "Cancer"[All Fields] OR "Cancers"[All Fields]) AND "androgen resistant prostatic"[Title/Abstract]) OR (("cancer s"[All Fields] OR "cancerated"[All Fields] OR "canceration"[All Fields] OR "cancerization"[All Fields] OR "cancerized"[All Fields] OR "cancerous"[All Fields] OR "Neoplasms"[MeSH Terms] OR "Neoplasms"[All Fields] OR "Cancer"[All Fields] OR "Cancers"[All Fields]) AND "androgen resistant prostatic"[Title/Abstract]) OR (("prostat"[All Fields] OR "prostate"[MeSH Terms] OR "prostate"[All Fields] OR "prostates"[All Fields] OR "Prostatic"[All Fields] OR "prostatism"[MeSH Terms] OR "prostatism"[All Fields] OR "prostatitis"[MeSH Terms] OR "prostatitis"[All Fields]) AND "cancer androgen resistant"[Title/Abstract]) OR (("prostatic neoplasms"[MeSH Terms] OR ("Prostatic"[All Fields] AND "Neoplasms"[All Fields]) OR "prostatic neoplasms"[All Fields] OR ("Prostatic"[All Fields] AND "Cancers"[All Fields]) OR "prostatic cancers"[All Fields]) AND "Androgen-Resistant"[Title/Abstract]) OR "androgen independent prostatic cancer"[Title/Abstract] OR "androgen independent prostatic cancer"[Title/Abstract] OR "castration resistant prostatic cancer"[Title/Abstract] OR "castration resistant prostatic cancer"[Title/Abstract] OR "androgen independent pc"[Title/Abstract] OR "androgen independent pca"[Title/Abstract] OR "androgen independent prostate cancer"[Title/Abstract] OR "androgen independent prostatic cancer"[Title/Abstract] OR "androgen insensitive pc"[Title/Abstract] OR "androgen insensitive pca"[Title/Abstract] OR "androgen insensitive prostate cancer"[Title/Abstract] OR "androgen insensitive prostatic cancer"[Title/Abstract] OR ("castrate refractory"[All Fields] AND "pc"[Title/Abstract]) OR ("castrate refractory"[All Fields] AND "pca"[Title/Abstract]) OR "castrate refractory prostate cancer"[Title/Abstract] OR ("castrate refractory"[All Fields] AND "prostatic cancer"[Title/Abstract]) OR "castrate resistant pc"[Title/Abstract] OR "castrate resistant pca"[Title/Abstract] OR "castrate resistant prostate cancer"[Title/Abstract] OR ("castration refractory"[All Fields] AND "pc"[Title/Abstract]) OR ("castration refractory"[All Fields] AND "pca"[Title/Abstract]) OR "castration refractory prostate cancer"[Title/Abstract] OR ("castration refractory"[All Fields] AND "prostatic cancer"[Title/Abstract]) OR "castration resistant pc"[Title/Abstract] OR "castration resistant pca"[Title/Abstract] OR "castration resistant prostatic cancer"[Title/Abstract] OR "castration resistant prostatic neoplasms"[Title/Abstract] OR "crpc"[All Fields]) AND (("castrate"[All Fields] OR "castrated"[All Fields] OR "castrates"[All Fields] OR "castrating"[All Fields] OR "Castration"[MeSH Terms] OR "Castration"[All Fields] OR "castrations"[All Fields] OR "castrator"[All Fields] OR "castrators"[All Fields] OR "orchiectomy"[MeSH Terms] OR "orchiectomy"[All Fields]) AND ("resist"[All Fields] OR "resistance"[All Fields] OR "resistances"[All Fields] OR "Resistant"[All Fields] OR "resistants"[All Fields] OR "resisted"[All Fields] OR "resistence"[All Fields] OR "resistences"[All Fields] OR "resistent"[All Fields] OR "resistibility"[All Fields] OR "resisting"[All Fields] OR "resistive"[All Fields] OR "resistively"[All Fields] OR "resistivities"[All Fields] OR "resistivity"[All Fields] OR "resists"[All Fields]) AND ("prostatic neoplasms"[MeSH Terms] OR ("Prostatic"[All Fields] AND "Neoplasms"[All Fields]) OR "prostatic neoplasms"[All Fields] OR ("prostate"[All Fields] AND "Cancer"[All Fields]) OR "prostate cancer"[All Fields]))) OR "hormone refractory prostate cancer"[Title/Abstract] OR "hormone refractory pc"[Title/Abstract] OR "hormone refractory pca"[Title/Abstract] OR "hormone refractory prostatic cancer"[Title/Abstract] OR ("hormone resistant"[All Fields] AND "pc"[Title/Abstract]) OR "hormone resistant pca"[Title/Abstract] OR "hormone resistant prostate cancer"[Title/Abstract] OR "hormone resistant prostatic cancer"[Title/Abstract] OR "prostatic neoplasms castration resistant"[Title/Abstract])) AND ("androgen antagonists"[MeSH Terms] OR ("antagonists androgen"[Title/Abstract] OR "antiandrogens"[Title/Abstract] OR "androgen antagonist"[Title/Abstract] OR "antagonist androgen"[Title/Abstract] OR "antiandrogen"[Title/Abstract] OR "anti androgen effect"[Title/Abstract] OR "anti androgen effect"[Title/Abstract] OR (("effect"[All Fields] OR "effecting"[All Fields] OR "effective"[All Fields] OR "effectively"[All Fields] OR "effectiveness"[All Fields] OR "effectivenesses"[All Fields] OR "effectives"[All Fields] OR "effectivities"[All Fields] OR "effectivity"[All Fields] OR "effects"[All Fields]) AND "anti androgen"[Title/Abstract]) OR "antiandrogen effect"[Title/Abstract] OR (("effect"[All Fields] OR "effecting"[All Fields] OR "effective"[All Fields] OR "effectively"[All Fields] OR "effectiveness"[All Fields] OR "effectivenesses"[All Fields] OR "effectives"[All Fields] OR "effectivities"[All Fields] OR "effectivity"[All Fields] OR "effects"[All Fields]) AND "antiandrogen"[Title/Abstract]) OR "antiandrogen effects"[Title/Abstract] OR "effects antiandrogen"[Title/Abstract] OR "anti androgen effects"[Title/Abstract] OR "anti androgen effects"[Title/Abstract] OR (("effect"[All Fields] OR "effecting"[All Fields] OR "effective"[All Fields] OR "effectively"[All Fields] OR "effectiveness"[All Fields] OR "effectivenesses"[All Fields] OR "effectives"[All Fields] OR "effectivities"[All Fields] OR "effectivity"[All Fields] OR "effects"[All Fields]) AND "anti androgen"[Title/Abstract]) OR "androgen antagonist"[Title/Abstract] OR "androgen antagonists"[Title/Abstract] OR "anti androgen"[Title/Abstract] OR "antiandrogen agent"[Title/Abstract] OR "antiandrogenic agent"[Title/Abstract] OR "antiandrogenic drug"[Title/Abstract] OR "antiandrogens"[Title/Abstract] OR "nonsteroidal anti androgen"[Title/Abstract] OR "nonsteroidal anti androgens"[Title/Abstract] OR "nonsteroidal anti androgen"[Title/Abstract] OR "nonsteroidal anti androgens"[Title/Abstract] OR "nonsteroidal antiandrogen"[Title/Abstract] OR "nonsteroidal antiandrogens"[Title/Abstract])) AND "random*"[Title/Abstract] | 283 | 5:24:51 |
| 8 | random*[Title/Abstract] | "random*"[Title/Abstract] | 1,360,835 | 5:24:36 |
| 7 | #5 AND #6 | ("prostatic neoplasms, castration resistant"[MeSH Terms] OR ((("castration resistant prostatic neoplasm"[Title/Abstract] OR (("neoplasm s"[All Fields] OR "Neoplasms"[MeSH Terms] OR "Neoplasms"[All Fields] OR "neoplasm"[All Fields]) AND "castration resistant prostatic"[Title/Abstract]) OR (("neoplasm s"[All Fields] OR "Neoplasms"[MeSH Terms] OR "Neoplasms"[All Fields] OR "neoplasm"[All Fields]) AND "castration resistant prostatic"[Title/Abstract]) OR (("prostatic neoplasms"[MeSH Terms] OR ("Prostatic"[All Fields] AND "Neoplasms"[All Fields]) OR "prostatic neoplasms"[All Fields] OR ("Prostatic"[All Fields] AND "neoplasm"[All Fields]) OR "prostatic neoplasm"[All Fields]) AND "Castration-Resistant"[Title/Abstract]) OR "prostatic neoplasms castration resistant"[Title/Abstract] OR ("Androgen-Insensitive"[All Fields] AND "prostatic neoplasms"[Title/Abstract]) OR ((("androgen s"[All Fields] OR "androgene"[All Fields] OR "androgenes"[All Fields] OR "androgenic"[All Fields] OR "androgenicity"[All Fields] OR "androgenized"[All Fields] OR "androgenizing"[All Fields] OR "androgenous"[All Fields] OR "androgens"[Pharmacological Action] OR "androgens"[MeSH Terms] OR "androgens"[All Fields] OR "Androgen"[All Fields] OR "virilism"[MeSH Terms] OR "virilism"[All Fields] OR "androgenization"[All Fields]) AND ("Insensitive"[All Fields] OR "insensitivities"[All Fields] OR "insensitivity"[All Fields])) AND "prostatic neoplasms"[Title/Abstract]) OR ("Androgen-Resistant"[All Fields] AND "prostatic neoplasms"[Title/Abstract]) OR (("androgen s"[All Fields] OR "androgene"[All Fields] OR "androgenes"[All Fields] OR "androgenic"[All Fields] OR "androgenicity"[All Fields] OR "androgenized"[All Fields] OR "androgenizing"[All Fields] OR "androgenous"[All Fields] OR "androgens"[Pharmacological Action] OR "androgens"[MeSH Terms] OR "androgens"[All Fields] OR "Androgen"[All Fields] OR "virilism"[MeSH Terms] OR "virilism"[All Fields] OR "androgenization"[All Fields]) AND "resistant prostatic neoplasms"[Title/Abstract]) OR (("prostatic neoplasms"[MeSH Terms] OR ("Prostatic"[All Fields] AND "Neoplasms"[All Fields]) OR "prostatic neoplasms"[All Fields]) AND "hormone refractory"[Title/Abstract]) OR ((("hormon"[All Fields] OR "hormonal"[All Fields] OR "hormonally"[All Fields] OR "hormonals"[All Fields] OR "hormone s"[All Fields] OR "hormones"[Pharmacological Action] OR "hormones"[MeSH Terms] OR "hormones"[All Fields] OR "Hormone"[All Fields] OR "hormons"[All Fields]) AND ("refractories"[All Fields] OR "refractoriness"[All Fields] OR "Refractory"[All Fields])) AND "prostatic neoplasms"[Title/Abstract]) OR (("prostatic neoplasms"[MeSH Terms] OR ("Prostatic"[All Fields] AND "Neoplasms"[All Fields]) OR "prostatic neoplasms"[All Fields]) AND "Androgen-Independent"[Title/Abstract]) OR ("Androgen-Independent"[All Fields] AND "prostatic neoplasm"[Title/Abstract]) OR (("neoplasm s"[All Fields] OR "Neoplasms"[MeSH Terms] OR "Neoplasms"[All Fields] OR "neoplasm"[All Fields]) AND "androgen independent prostatic"[Title/Abstract]) OR (("neoplasm s"[All Fields] OR "Neoplasms"[MeSH Terms] OR "Neoplasms"[All Fields] OR "neoplasm"[All Fields]) AND "androgen independent prostatic"[Title/Abstract]) OR (("prostatic neoplasms"[MeSH Terms] OR ("Prostatic"[All Fields] AND "Neoplasms"[All Fields]) OR "prostatic neoplasms"[All Fields] OR ("Prostatic"[All Fields] AND "neoplasm"[All Fields]) OR "prostatic neoplasm"[All Fields]) AND "Androgen-Independent"[Title/Abstract]) OR (("prostatic neoplasms"[MeSH Terms] OR ("Prostatic"[All Fields] AND "Neoplasms"[All Fields]) OR "prostatic neoplasms"[All Fields]) AND "Androgen-Independent"[Title/Abstract]) OR (("prostatic neoplasms"[MeSH Terms] OR ("Prostatic"[All Fields] AND "Neoplasms"[All Fields]) OR "prostatic neoplasms"[All Fields]) AND "Androgen-Insensitive"[Title/Abstract]) OR ("Androgen-Insensitive"[All Fields] AND "prostatic neoplasm"[Title/Abstract]) OR (("neoplasm s"[All Fields] OR "Neoplasms"[MeSH Terms] OR "Neoplasms"[All Fields] OR "neoplasm"[All Fields]) AND "androgen insensitive prostatic"[Title/Abstract]) OR (("neoplasm s"[All Fields] OR "Neoplasms"[MeSH Terms] OR "Neoplasms"[All Fields] OR "neoplasm"[All Fields]) AND "androgen insensitive prostatic"[Title/Abstract]) OR (("prostatic neoplasms"[MeSH Terms] OR ("Prostatic"[All Fields] AND "Neoplasms"[All Fields]) OR "prostatic neoplasms"[All Fields] OR ("Prostatic"[All Fields] AND "neoplasm"[All Fields]) OR "prostatic neoplasm"[All Fields]) AND "Androgen-Insensitive"[Title/Abstract]) OR (("prostatic neoplasms"[MeSH Terms] OR ("Prostatic"[All Fields] AND "Neoplasms"[All Fields]) OR "prostatic neoplasms"[All Fields]) AND "Androgen-Insensitive"[Title/Abstract]) OR (("prostatic neoplasms"[MeSH Terms] OR ("Prostatic"[All Fields] AND "Neoplasms"[All Fields]) OR "prostatic neoplasms"[All Fields]) AND "Androgen-Resistant"[Title/Abstract]) OR ("Androgen-Resistant"[All Fields] AND "prostatic neoplasm"[Title/Abstract]) OR (("neoplasm s"[All Fields] OR "Neoplasms"[MeSH Terms] OR "Neoplasms"[All Fields] OR "neoplasm"[All Fields]) AND "androgen resistant prostatic"[Title/Abstract]) OR (("neoplasm s"[All Fields] OR "Neoplasms"[MeSH Terms] OR "Neoplasms"[All Fields] OR "neoplasm"[All Fields]) AND "androgen resistant prostatic"[Title/Abstract]) OR (("prostatic neoplasms"[MeSH Terms] OR ("Prostatic"[All Fields] AND "Neoplasms"[All Fields]) OR "prostatic neoplasms"[All Fields] OR ("Prostatic"[All Fields] AND "neoplasm"[All Fields]) OR "prostatic neoplasm"[All Fields]) AND "Androgen-Resistant"[Title/Abstract]) OR (("prostatic neoplasms"[MeSH Terms] OR ("Prostatic"[All Fields] AND "Neoplasms"[All Fields]) OR "prostatic neoplasms"[All Fields]) AND "Androgen-Resistant"[Title/Abstract]) OR "androgen independent prostatic neoplasms"[Title/Abstract] OR "androgen independent prostatic neoplasms"[Title/Abstract] OR "castration resistant prostatic neoplasms"[Title/Abstract] OR "castration resistant prostatic neoplasms"[Title/Abstract] OR "prostatic cancer castration resistant"[Title/Abstract] OR (("cancer s"[All Fields] OR "cancerated"[All Fields] OR "canceration"[All Fields] OR "cancerization"[All Fields] OR "cancerized"[All Fields] OR "cancerous"[All Fields] OR "Neoplasms"[MeSH Terms] OR "Neoplasms"[All Fields] OR "Cancer"[All Fields] OR "Cancers"[All Fields]) AND "castration resistant prostatic"[Title/Abstract]) OR (("cancer s"[All Fields] OR "cancerated"[All Fields] OR "canceration"[All Fields] OR "cancerization"[All Fields] OR "cancerized"[All Fields] OR "cancerous"[All Fields] OR "Neoplasms"[MeSH Terms] OR "Neoplasms"[All Fields] OR "Cancer"[All Fields] OR "Cancers"[All Fields]) AND "castration resistant prostatic"[Title/Abstract]) OR ("Castration-Resistant"[All Fields] AND "prostatic cancers"[Title/Abstract]) OR "prostatic cancer castration resistant"[Title/Abstract] OR (("prostatic neoplasms"[MeSH Terms] OR ("Prostatic"[All Fields] AND "Neoplasms"[All Fields]) OR "prostatic neoplasms"[All Fields] OR ("Prostatic"[All Fields] AND "Cancers"[All Fields]) OR "prostatic cancers"[All Fields]) AND "Castration-Resistant"[Title/Abstract]) OR "androgen insensitive prostatic cancer"[Title/Abstract] OR "androgen insensitive prostatic cancer"[Title/Abstract] OR "androgen resistant prostatic cancer"[Title/Abstract] OR "androgen resistant prostatic cancer"[Title/Abstract] OR (("prostatic neoplasms"[MeSH Terms] OR ("Prostatic"[All Fields] AND "Neoplasms"[All Fields]) OR "prostatic neoplasms"[All Fields] OR ("Prostatic"[All Fields] AND "Cancer"[All Fields]) OR "prostatic cancer"[All Fields]) AND "hormone refractory"[Title/Abstract]) OR "hormone refractory prostatic cancer"[Title/Abstract] OR (("prostat"[All Fields] OR "prostate"[MeSH Terms] OR "prostate"[All Fields] OR "prostates"[All Fields] OR "Prostatic"[All Fields] OR "prostatism"[MeSH Terms] OR "prostatism"[All Fields] OR "prostatitis"[MeSH Terms] OR "prostatitis"[All Fields]) AND "cancer androgen independent"[Title/Abstract]) OR "androgen independent prostatic cancers"[Title/Abstract] OR (("cancer s"[All Fields] OR "cancerated"[All Fields] OR "canceration"[All Fields] OR "cancerization"[All Fields] OR "cancerized"[All Fields] OR "cancerous"[All Fields] OR "Neoplasms"[MeSH Terms] OR "Neoplasms"[All Fields] OR "Cancer"[All Fields] OR "Cancers"[All Fields]) AND "androgen independent prostatic"[Title/Abstract]) OR (("cancer s"[All Fields] OR "cancerated"[All Fields] OR "canceration"[All Fields] OR "cancerization"[All Fields] OR "cancerized"[All Fields] OR "cancerous"[All Fields] OR "Neoplasms"[MeSH Terms] OR "Neoplasms"[All Fields] OR "Cancer"[All Fields] OR "Cancers"[All Fields]) AND "androgen independent prostatic"[Title/Abstract]) OR (("prostat"[All Fields] OR "prostate"[MeSH Terms] OR "prostate"[All Fields] OR "prostates"[All Fields] OR "Prostatic"[All Fields] OR "prostatism"[MeSH Terms] OR "prostatism"[All Fields] OR "prostatitis"[MeSH Terms] OR "prostatitis"[All Fields]) AND "cancer androgen independent"[Title/Abstract]) OR (("prostat"[All Fields] OR "prostate"[MeSH Terms] OR "prostate"[All Fields] OR "prostates"[All Fields] OR "Prostatic"[All Fields] OR "prostatism"[MeSH Terms] OR "prostatism"[All Fields] OR "prostatitis"[MeSH Terms] OR "prostatitis"[All Fields]) AND "cancers androgen independent"[Title/Abstract]) OR (("prostat"[All Fields] OR "prostate"[MeSH Terms] OR "prostate"[All Fields] OR "prostates"[All Fields] OR "Prostatic"[All Fields] OR "prostatism"[MeSH Terms] OR "prostatism"[All Fields] OR "prostatitis"[MeSH Terms] OR "prostatitis"[All Fields]) AND "cancer androgen insensitive"[Title/Abstract]) OR ("Androgen-Insensitive"[All Fields] AND "prostatic cancers"[Title/Abstract]) OR (("cancer s"[All Fields] OR "cancerated"[All Fields] OR "canceration"[All Fields] OR "cancerization"[All Fields] OR "cancerized"[All Fields] OR "cancerous"[All Fields] OR "Neoplasms"[MeSH Terms] OR "Neoplasms"[All Fields] OR "Cancer"[All Fields] OR "Cancers"[All Fields]) AND "androgen insensitive prostatic"[Title/Abstract]) OR (("cancer s"[All Fields] OR "cancerated"[All Fields] OR "canceration"[All Fields] OR "cancerization"[All Fields] OR "cancerized"[All Fields] OR "cancerous"[All Fields] OR "Neoplasms"[MeSH Terms] OR "Neoplasms"[All Fields] OR "Cancer"[All Fields] OR "Cancers"[All Fields]) AND "androgen insensitive prostatic"[Title/Abstract]) OR (("prostat"[All Fields] OR "prostate"[MeSH Terms] OR "prostate"[All Fields] OR "prostates"[All Fields] OR "Prostatic"[All Fields] OR "prostatism"[MeSH Terms] OR "prostatism"[All Fields] OR "prostatitis"[MeSH Terms] OR "prostatitis"[All Fields]) AND "cancer androgen insensitive"[Title/Abstract]) OR (("prostatic neoplasms"[MeSH Terms] OR ("Prostatic"[All Fields] AND "Neoplasms"[All Fields]) OR "prostatic neoplasms"[All Fields] OR ("Prostatic"[All Fields] AND "Cancers"[All Fields]) OR "prostatic cancers"[All Fields]) AND "Androgen-Insensitive"[Title/Abstract]) OR (("prostat"[All Fields] OR "prostate"[MeSH Terms] OR "prostate"[All Fields] OR "prostates"[All Fields] OR "Prostatic"[All Fields] OR "prostatism"[MeSH Terms] OR "prostatism"[All Fields] OR "prostatitis"[MeSH Terms] OR "prostatitis"[All Fields]) AND "cancer androgen resistant"[Title/Abstract]) OR ("Androgen-Resistant"[All Fields] AND "prostatic cancers"[Title/Abstract]) OR (("cancer s"[All Fields] OR "cancerated"[All Fields] OR "canceration"[All Fields] OR "cancerization"[All Fields] OR "cancerized"[All Fields] OR "cancerous"[All Fields] OR "Neoplasms"[MeSH Terms] OR "Neoplasms"[All Fields] OR "Cancer"[All Fields] OR "Cancers"[All Fields]) AND "androgen resistant prostatic"[Title/Abstract]) OR (("cancer s"[All Fields] OR "cancerated"[All Fields] OR "canceration"[All Fields] OR "cancerization"[All Fields] OR "cancerized"[All Fields] OR "cancerous"[All Fields] OR "Neoplasms"[MeSH Terms] OR "Neoplasms"[All Fields] OR "Cancer"[All Fields] OR "Cancers"[All Fields]) AND "androgen resistant prostatic"[Title/Abstract]) OR (("prostat"[All Fields] OR "prostate"[MeSH Terms] OR "prostate"[All Fields] OR "prostates"[All Fields] OR "Prostatic"[All Fields] OR "prostatism"[MeSH Terms] OR "prostatism"[All Fields] OR "prostatitis"[MeSH Terms] OR "prostatitis"[All Fields]) AND "cancer androgen resistant"[Title/Abstract]) OR (("prostatic neoplasms"[MeSH Terms] OR ("Prostatic"[All Fields] AND "Neoplasms"[All Fields]) OR "prostatic neoplasms"[All Fields] OR ("Prostatic"[All Fields] AND "Cancers"[All Fields]) OR "prostatic cancers"[All Fields]) AND "Androgen-Resistant"[Title/Abstract]) OR "androgen independent prostatic cancer"[Title/Abstract] OR "androgen independent prostatic cancer"[Title/Abstract] OR "castration resistant prostatic cancer"[Title/Abstract] OR "castration resistant prostatic cancer"[Title/Abstract] OR "androgen independent pc"[Title/Abstract] OR "androgen independent pca"[Title/Abstract] OR "androgen independent prostate cancer"[Title/Abstract] OR "androgen independent prostatic cancer"[Title/Abstract] OR "androgen insensitive pc"[Title/Abstract] OR "androgen insensitive pca"[Title/Abstract] OR "androgen insensitive prostate cancer"[Title/Abstract] OR "androgen insensitive prostatic cancer"[Title/Abstract] OR ("castrate refractory"[All Fields] AND "pc"[Title/Abstract]) OR ("castrate refractory"[All Fields] AND "pca"[Title/Abstract]) OR "castrate refractory prostate cancer"[Title/Abstract] OR ("castrate refractory"[All Fields] AND "prostatic cancer"[Title/Abstract]) OR "castrate resistant pc"[Title/Abstract] OR "castrate resistant pca"[Title/Abstract] OR "castrate resistant prostate cancer"[Title/Abstract] OR ("castration refractory"[All Fields] AND "pc"[Title/Abstract]) OR ("castration refractory"[All Fields] AND "pca"[Title/Abstract]) OR "castration refractory prostate cancer"[Title/Abstract] OR ("castration refractory"[All Fields] AND "prostatic cancer"[Title/Abstract]) OR "castration resistant pc"[Title/Abstract] OR "castration resistant pca"[Title/Abstract] OR "castration resistant prostatic cancer"[Title/Abstract] OR "castration resistant prostatic neoplasms"[Title/Abstract] OR "crpc"[All Fields]) AND (("castrate"[All Fields] OR "castrated"[All Fields] OR "castrates"[All Fields] OR "castrating"[All Fields] OR "Castration"[MeSH Terms] OR "Castration"[All Fields] OR "castrations"[All Fields] OR "castrator"[All Fields] OR "castrators"[All Fields] OR "orchiectomy"[MeSH Terms] OR "orchiectomy"[All Fields]) AND ("resist"[All Fields] OR "resistance"[All Fields] OR "resistances"[All Fields] OR "Resistant"[All Fields] OR "resistants"[All Fields] OR "resisted"[All Fields] OR "resistence"[All Fields] OR "resistences"[All Fields] OR "resistent"[All Fields] OR "resistibility"[All Fields] OR "resisting"[All Fields] OR "resistive"[All Fields] OR "resistively"[All Fields] OR "resistivities"[All Fields] OR "resistivity"[All Fields] OR "resists"[All Fields]) AND ("prostatic neoplasms"[MeSH Terms] OR ("Prostatic"[All Fields] AND "Neoplasms"[All Fields]) OR "prostatic neoplasms"[All Fields] OR ("prostate"[All Fields] AND "Cancer"[All Fields]) OR "prostate cancer"[All Fields]))) OR "hormone refractory prostate cancer"[Title/Abstract] OR "hormone refractory pc"[Title/Abstract] OR "hormone refractory pca"[Title/Abstract] OR "hormone refractory prostatic cancer"[Title/Abstract] OR ("hormone resistant"[All Fields] AND "pc"[Title/Abstract]) OR "hormone resistant pca"[Title/Abstract] OR "hormone resistant prostate cancer"[Title/Abstract] OR "hormone resistant prostatic cancer"[Title/Abstract] OR "prostatic neoplasms castration resistant"[Title/Abstract])) AND ("androgen antagonists"[MeSH Terms] OR ("antagonists androgen"[Title/Abstract] OR "antiandrogens"[Title/Abstract] OR "androgen antagonist"[Title/Abstract] OR "antagonist androgen"[Title/Abstract] OR "antiandrogen"[Title/Abstract] OR "anti androgen effect"[Title/Abstract] OR "anti androgen effect"[Title/Abstract] OR (("effect"[All Fields] OR "effecting"[All Fields] OR "effective"[All Fields] OR "effectively"[All Fields] OR "effectiveness"[All Fields] OR "effectivenesses"[All Fields] OR "effectives"[All Fields] OR "effectivities"[All Fields] OR "effectivity"[All Fields] OR "effects"[All Fields]) AND "anti androgen"[Title/Abstract]) OR "antiandrogen effect"[Title/Abstract] OR (("effect"[All Fields] OR "effecting"[All Fields] OR "effective"[All Fields] OR "effectively"[All Fields] OR "effectiveness"[All Fields] OR "effectivenesses"[All Fields] OR "effectives"[All Fields] OR "effectivities"[All Fields] OR "effectivity"[All Fields] OR "effects"[All Fields]) AND "antiandrogen"[Title/Abstract]) OR "antiandrogen effects"[Title/Abstract] OR "effects antiandrogen"[Title/Abstract] OR "anti androgen effects"[Title/Abstract] OR "anti androgen effects"[Title/Abstract] OR (("effect"[All Fields] OR "effecting"[All Fields] OR "effective"[All Fields] OR "effectively"[All Fields] OR "effectiveness"[All Fields] OR "effectivenesses"[All Fields] OR "effectives"[All Fields] OR "effectivities"[All Fields] OR "effectivity"[All Fields] OR "effects"[All Fields]) AND "anti androgen"[Title/Abstract]) OR "androgen antagonist"[Title/Abstract] OR "androgen antagonists"[Title/Abstract] OR "anti androgen"[Title/Abstract] OR "antiandrogen agent"[Title/Abstract] OR "antiandrogenic agent"[Title/Abstract] OR "antiandrogenic drug"[Title/Abstract] OR "antiandrogens"[Title/Abstract] OR "nonsteroidal anti androgen"[Title/Abstract] OR "nonsteroidal anti androgens"[Title/Abstract] OR "nonsteroidal anti androgen"[Title/Abstract] OR "nonsteroidal anti androgens"[Title/Abstract] OR "nonsteroidal antiandrogen"[Title/Abstract] OR "nonsteroidal antiandrogens"[Title/Abstract])) | 2,531 | 5:24:23 |
| 6 | (Androgen Antagonists[MeSH Terms]) OR (‘Antagonists, Androgen’[Title/Abstract] OR ‘Antiandrogens’[Title/Abstract] OR ‘Androgen Antagonist’[Title/Abstract] OR ‘Antagonist, Androgen’[Title/Abstract] OR ‘Antiandrogen’[Title/Abstract] OR ‘Anti-Androgen Effect’[Title/Abstract] OR ‘Anti Androgen Effect’[Title/Abstract] OR ‘Effect, Anti-Androgen’[Title/Abstract] OR ‘Antiandrogen Effect’[Title/Abstract] OR ‘Effect, Antiandrogen’[Title/Abstract] OR ‘Antiandrogen Effects’[Title/Abstract] OR ‘Effects, Antiandrogen’[Title/Abstract] OR ‘Anti-Androgen Effects’[Title/Abstract] OR ‘Anti Androgen Effects’[Title/Abstract] OR ‘Effects, Anti-Androgen’[Title/Abstract] OR ‘androgen antagonist’[Title/Abstract] OR ‘androgen antagonists’[Title/Abstract] OR ‘anti androgen’[Title/Abstract] OR ‘antiandrogen agent’[Title/Abstract] OR ‘antiandrogenic agent’[Title/Abstract] OR ‘antiandrogenic drug’[Title/Abstract] OR ‘antiandrogens’[Title/Abstract] OR ‘nonsteroidal anti androgen’[Title/Abstract] OR ‘nonsteroidal anti androgens’[Title/Abstract] OR ‘nonsteroidal anti-androgen’[Title/Abstract] OR ‘nonsteroidal anti-androgens’[Title/Abstract] OR ‘nonsteroidal antiandrogen’[Title/Abstract] OR ‘nonsteroidal antiandrogens’[Title/Abstract]) | "androgen antagonists"[MeSH Terms] OR ("antagonists androgen"[Title/Abstract] OR "antiandrogens"[Title/Abstract] OR "androgen antagonist"[Title/Abstract] OR "antagonist androgen"[Title/Abstract] OR "antiandrogen"[Title/Abstract] OR "anti androgen effect"[Title/Abstract] OR "anti androgen effect"[Title/Abstract] OR (("effect"[All Fields] OR "effecting"[All Fields] OR "effective"[All Fields] OR "effectively"[All Fields] OR "effectiveness"[All Fields] OR "effectivenesses"[All Fields] OR "effectives"[All Fields] OR "effectivities"[All Fields] OR "effectivity"[All Fields] OR "effects"[All Fields]) AND "anti androgen"[Title/Abstract]) OR "antiandrogen effect"[Title/Abstract] OR (("effect"[All Fields] OR "effecting"[All Fields] OR "effective"[All Fields] OR "effectively"[All Fields] OR "effectiveness"[All Fields] OR "effectivenesses"[All Fields] OR "effectives"[All Fields] OR "effectivities"[All Fields] OR "effectivity"[All Fields] OR "effects"[All Fields]) AND "antiandrogen"[Title/Abstract]) OR "antiandrogen effects"[Title/Abstract] OR "effects antiandrogen"[Title/Abstract] OR "anti androgen effects"[Title/Abstract] OR "anti androgen effects"[Title/Abstract] OR (("effect"[All Fields] OR "effecting"[All Fields] OR "effective"[All Fields] OR "effectively"[All Fields] OR "effectiveness"[All Fields] OR "effectivenesses"[All Fields] OR "effectives"[All Fields] OR "effectivities"[All Fields] OR "effectivity"[All Fields] OR "effects"[All Fields]) AND "anti androgen"[Title/Abstract]) OR "androgen antagonist"[Title/Abstract] OR "androgen antagonists"[Title/Abstract] OR "anti androgen"[Title/Abstract] OR "antiandrogen agent"[Title/Abstract] OR "antiandrogenic agent"[Title/Abstract] OR "antiandrogenic drug"[Title/Abstract] OR "antiandrogens"[Title/Abstract] OR "nonsteroidal anti androgen"[Title/Abstract] OR "nonsteroidal anti androgens"[Title/Abstract] OR "nonsteroidal anti androgen"[Title/Abstract] OR "nonsteroidal anti androgens"[Title/Abstract] OR "nonsteroidal antiandrogen"[Title/Abstract] OR "nonsteroidal antiandrogens"[Title/Abstract]) | 17,656 | 5:24:00 |
| 5 | (Prostatic Neoplasms, Castration-Resistant[MeSH Terms]) OR (‘Castration-Resistant Prostatic Neoplasm’[Title/Abstract] OR ‘Neoplasm, Castration-Resistant Prostatic’[Title/Abstract] OR ‘Neoplasms, Castration-Resistant Prostatic’[Title/Abstract] OR ‘Prostatic Neoplasm, Castration-Resistant’[Title/Abstract] OR ‘Prostatic Neoplasms, Castration Resistant’[Title/Abstract] OR ‘Androgen-Insensitive Prostatic Neoplasms’[Title/Abstract] OR ‘Androgen Insensitive Prostatic Neoplasms’[Title/Abstract] OR ‘Androgen-Resistant Prostatic Neoplasms’[Title/Abstract] OR ‘Androgen Resistant Prostatic Neoplasms’[Title/Abstract] OR ‘Prostatic Neoplasms, Hormone Refractory’[Title/Abstract] OR ‘Hormone Refractory Prostatic Neoplasms’[Title/Abstract] OR ‘Prostatic Neoplasms, Androgen-Independent’[Title/Abstract] OR ‘Androgen-Independent Prostatic Neoplasm’[Title/Abstract] OR ‘Neoplasm, Androgen-Independent Prostatic’[Title/Abstract] OR ‘Neoplasms, Androgen-Independent Prostatic’[Title/Abstract] OR ‘Prostatic Neoplasm, Androgen-Independent’[Title/Abstract] OR ‘Prostatic Neoplasms, Androgen Independent’[Title/Abstract] OR ‘Prostatic Neoplasms, Androgen-Insensitive’[Title/Abstract] OR ‘Androgen-Insensitive Prostatic Neoplasm’[Title/Abstract] OR ‘Neoplasm, Androgen-Insensitive Prostatic’[Title/Abstract] OR ‘Neoplasms, Androgen-Insensitive Prostatic’[Title/Abstract] OR ‘Prostatic Neoplasm, Androgen-Insensitive’[Title/Abstract] OR ‘Prostatic Neoplasms, Androgen Insensitive’[Title/Abstract] OR ‘Prostatic Neoplasms, Androgen-Resistant’[Title/Abstract] OR ‘Androgen-Resistant Prostatic Neoplasm’[Title/Abstract] OR ‘Neoplasm, Androgen-Resistant Prostatic’[Title/Abstract] OR ‘Neoplasms, Androgen-Resistant Prostatic’[Title/Abstract] OR ‘Prostatic Neoplasm, Androgen-Resistant’[Title/Abstract] OR ‘Prostatic Neoplasms, Androgen Resistant’[Title/Abstract] OR ‘Androgen-Independent Prostatic Neoplasms’[Title/Abstract] OR ‘Androgen Independent Prostatic Neoplasms’[Title/Abstract] OR ‘Castration-Resistant Prostatic Neoplasms’[Title/Abstract] OR ‘Castration Resistant Prostatic Neoplasms’[Title/Abstract] OR ‘Prostatic Cancer, Castration-Resistant’[Title/Abstract] OR ‘Cancer, Castration-Resistant Prostatic’[Title/Abstract] OR ‘Cancers, Castration-Resistant Prostatic’[Title/Abstract] OR ‘Castration-Resistant Prostatic Cancers’[Title/Abstract] OR ‘Prostatic Cancer, Castration Resistant’[Title/Abstract] OR ‘Prostatic Cancers, Castration-Resistant’[Title/Abstract] OR ‘Androgen-Insensitive Prostatic Cancer’[Title/Abstract] OR ‘Androgen Insensitive Prostatic Cancer’[Title/Abstract] OR ‘Androgen-Resistant Prostatic Cancer’[Title/Abstract] OR ‘Androgen Resistant Prostatic Cancer’[Title/Abstract] OR ‘Prostatic Cancer, Hormone Refractory’[Title/Abstract] OR ‘Hormone Refractory Prostatic Cancer’[Title/Abstract] OR ‘Prostatic Cancer, Androgen-Independent’[Title/Abstract] OR ‘Androgen-Independent Prostatic Cancers’[Title/Abstract] OR ‘Cancer, Androgen-Independent Prostatic’[Title/Abstract] OR ‘Cancers, Androgen-Independent Prostatic’[Title/Abstract] OR ‘Prostatic Cancer, Androgen Independent’[Title/Abstract] OR ‘Prostatic Cancers, Androgen-Independent’[Title/Abstract] OR ‘Prostatic Cancer, Androgen-Insensitive’[Title/Abstract] OR ‘Androgen-Insensitive Prostatic Cancers’[Title/Abstract] OR ‘Cancer, Androgen-Insensitive Prostatic’[Title/Abstract] OR ‘Cancers, Androgen-Insensitive Prostatic’[Title/Abstract] OR ‘Prostatic Cancer, Androgen Insensitive’[Title/Abstract] OR ‘Prostatic Cancers, Androgen-Insensitive’[Title/Abstract] OR ‘Prostatic Cancer, Androgen-Resistant’[Title/Abstract] OR ‘Androgen-Resistant Prostatic Cancers’[Title/Abstract] OR ‘Cancer, Androgen-Resistant Prostatic’[Title/Abstract] OR ‘Cancers, Androgen-Resistant Prostatic’[Title/Abstract] OR ‘Prostatic Cancer, Androgen Resistant’[Title/Abstract] OR ‘Prostatic Cancers, Androgen-Resistant’[Title/Abstract] OR ‘Androgen-Independent Prostatic Cancer’[Title/Abstract] OR ‘Androgen Independent Prostatic Cancer’[Title/Abstract] OR ‘Castration-Resistant Prostatic Cancer’[Title/Abstract] OR ‘Castration Resistant Prostatic Cancer’[Title/Abstract] OR ‘androgen-independent PC’[Title/Abstract] OR ‘androgen-independent PCa’[Title/Abstract] OR ‘androgen-independent prostate cancer’[Title/Abstract] OR ‘androgen-independent prostatic cancer’[Title/Abstract] OR ‘androgen-insensitive PC’[Title/Abstract] OR ‘androgen-insensitive PCa’[Title/Abstract] OR ‘androgen-insensitive prostate cancer’[Title/Abstract] OR ‘androgen-insensitive prostatic cancer’[Title/Abstract] OR ‘castrate-refractory PC’[Title/Abstract] OR ‘castrate-refractory PCa’[Title/Abstract] OR ‘castrate-refractory prostate cancer’[Title/Abstract] OR ‘castrate-refractory prostatic cancer’[Title/Abstract] OR ‘castrate-resistant PC’[Title/Abstract] OR ‘castrate-resistant PCa’[Title/Abstract] OR ‘castrate-resistant prostate cancer’[Title/Abstract] OR ‘castration-refractory PC’[Title/Abstract] OR ‘castration-refractory PCa’[Title/Abstract] OR ‘castration-refractory prostate cancer’[Title/Abstract] OR ‘castration-refractory prostatic cancer’[Title/Abstract] OR ‘castration-resistant PC’[Title/Abstract] OR ‘castration-resistant PCa’[Title/Abstract] OR ‘castration-resistant prostatic cancer’[Title/Abstract] OR ‘castration-resistant prostatic neoplasms’[Title/Abstract] OR ‘CRPC (castration resistant prostate cancer)’[Title/Abstract] OR ‘hormone refractory prostate cancer’[Title/Abstract] OR ‘hormone-refractory PC’[Title/Abstract] OR ‘hormone-refractory PCa’[Title/Abstract] OR ‘hormone-refractory prostatic cancer’[Title/Abstract] OR ‘hormone-resistant PC’[Title/Abstract] OR ‘hormone-resistant PCa’[Title/Abstract] OR ‘hormone-resistant prostate cancer’[Title/Abstract] OR ‘hormone-resistant prostatic cancer’[Title/Abstract] OR ‘prostatic neoplasms, castration-resistant’[Title/Abstract]) | "prostatic neoplasms, castration resistant"[MeSH Terms] OR ((("castration resistant prostatic neoplasm"[Title/Abstract] OR (("neoplasm s"[All Fields] OR "Neoplasms"[MeSH Terms] OR "Neoplasms"[All Fields] OR "neoplasm"[All Fields]) AND "castration resistant prostatic"[Title/Abstract]) OR (("neoplasm s"[All Fields] OR "Neoplasms"[MeSH Terms] OR "Neoplasms"[All Fields] OR "neoplasm"[All Fields]) AND "castration resistant prostatic"[Title/Abstract]) OR (("prostatic neoplasms"[MeSH Terms] OR ("Prostatic"[All Fields] AND "Neoplasms"[All Fields]) OR "prostatic neoplasms"[All Fields] OR ("Prostatic"[All Fields] AND "neoplasm"[All Fields]) OR "prostatic neoplasm"[All Fields]) AND "Castration-Resistant"[Title/Abstract]) OR "prostatic neoplasms castration resistant"[Title/Abstract] OR ("Androgen-Insensitive"[All Fields] AND "prostatic neoplasms"[Title/Abstract]) OR ((("androgen s"[All Fields] OR "androgene"[All Fields] OR "androgenes"[All Fields] OR "androgenic"[All Fields] OR "androgenicity"[All Fields] OR "androgenized"[All Fields] OR "androgenizing"[All Fields] OR "androgenous"[All Fields] OR "androgens"[Pharmacological Action] OR "androgens"[MeSH Terms] OR "androgens"[All Fields] OR "Androgen"[All Fields] OR "virilism"[MeSH Terms] OR "virilism"[All Fields] OR "androgenization"[All Fields]) AND ("Insensitive"[All Fields] OR "insensitivities"[All Fields] OR "insensitivity"[All Fields])) AND "prostatic neoplasms"[Title/Abstract]) OR ("Androgen-Resistant"[All Fields] AND "prostatic neoplasms"[Title/Abstract]) OR (("androgen s"[All Fields] OR "androgene"[All Fields] OR "androgenes"[All Fields] OR "androgenic"[All Fields] OR "androgenicity"[All Fields] OR "androgenized"[All Fields] OR "androgenizing"[All Fields] OR "androgenous"[All Fields] OR "androgens"[Pharmacological Action] OR "androgens"[MeSH Terms] OR "androgens"[All Fields] OR "Androgen"[All Fields] OR "virilism"[MeSH Terms] OR "virilism"[All Fields] OR "androgenization"[All Fields]) AND "resistant prostatic neoplasms"[Title/Abstract]) OR (("prostatic neoplasms"[MeSH Terms] OR ("Prostatic"[All Fields] AND "Neoplasms"[All Fields]) OR "prostatic neoplasms"[All Fields]) AND "hormone refractory"[Title/Abstract]) OR ((("hormon"[All Fields] OR "hormonal"[All Fields] OR "hormonally"[All Fields] OR "hormonals"[All Fields] OR "hormone s"[All Fields] OR "hormones"[Pharmacological Action] OR "hormones"[MeSH Terms] OR "hormones"[All Fields] OR "Hormone"[All Fields] OR "hormons"[All Fields]) AND ("refractories"[All Fields] OR "refractoriness"[All Fields] OR "Refractory"[All Fields])) AND "prostatic neoplasms"[Title/Abstract]) OR (("prostatic neoplasms"[MeSH Terms] OR ("Prostatic"[All Fields] AND "Neoplasms"[All Fields]) OR "prostatic neoplasms"[All Fields]) AND "Androgen-Independent"[Title/Abstract]) OR ("Androgen-Independent"[All Fields] AND "prostatic neoplasm"[Title/Abstract]) OR (("neoplasm s"[All Fields] OR "Neoplasms"[MeSH Terms] OR "Neoplasms"[All Fields] OR "neoplasm"[All Fields]) AND "androgen independent prostatic"[Title/Abstract]) OR (("neoplasm s"[All Fields] OR "Neoplasms"[MeSH Terms] OR "Neoplasms"[All Fields] OR "neoplasm"[All Fields]) AND "androgen independent prostatic"[Title/Abstract]) OR (("prostatic neoplasms"[MeSH Terms] OR ("Prostatic"[All Fields] AND "Neoplasms"[All Fields]) OR "prostatic neoplasms"[All Fields] OR ("Prostatic"[All Fields] AND "neoplasm"[All Fields]) OR "prostatic neoplasm"[All Fields]) AND "Androgen-Independent"[Title/Abstract]) OR (("prostatic neoplasms"[MeSH Terms] OR ("Prostatic"[All Fields] AND "Neoplasms"[All Fields]) OR "prostatic neoplasms"[All Fields]) AND "Androgen-Independent"[Title/Abstract]) OR (("prostatic neoplasms"[MeSH Terms] OR ("Prostatic"[All Fields] AND "Neoplasms"[All Fields]) OR "prostatic neoplasms"[All Fields]) AND "Androgen-Insensitive"[Title/Abstract]) OR ("Androgen-Insensitive"[All Fields] AND "prostatic neoplasm"[Title/Abstract]) OR (("neoplasm s"[All Fields] OR "Neoplasms"[MeSH Terms] OR "Neoplasms"[All Fields] OR "neoplasm"[All Fields]) AND "androgen insensitive prostatic"[Title/Abstract]) OR (("neoplasm s"[All Fields] OR "Neoplasms"[MeSH Terms] OR "Neoplasms"[All Fields] OR "neoplasm"[All Fields]) AND "androgen insensitive prostatic"[Title/Abstract]) OR (("prostatic neoplasms"[MeSH Terms] OR ("Prostatic"[All Fields] AND "Neoplasms"[All Fields]) OR "prostatic neoplasms"[All Fields] OR ("Prostatic"[All Fields] AND "neoplasm"[All Fields]) OR "prostatic neoplasm"[All Fields]) AND "Androgen-Insensitive"[Title/Abstract]) OR (("prostatic neoplasms"[MeSH Terms] OR ("Prostatic"[All Fields] AND "Neoplasms"[All Fields]) OR "prostatic neoplasms"[All Fields]) AND "Androgen-Insensitive"[Title/Abstract]) OR (("prostatic neoplasms"[MeSH Terms] OR ("Prostatic"[All Fields] AND "Neoplasms"[All Fields]) OR "prostatic neoplasms"[All Fields]) AND "Androgen-Resistant"[Title/Abstract]) OR ("Androgen-Resistant"[All Fields] AND "prostatic neoplasm"[Title/Abstract]) OR (("neoplasm s"[All Fields] OR "Neoplasms"[MeSH Terms] OR "Neoplasms"[All Fields] OR "neoplasm"[All Fields]) AND "androgen resistant prostatic"[Title/Abstract]) OR (("neoplasm s"[All Fields] OR "Neoplasms"[MeSH Terms] OR "Neoplasms"[All Fields] OR "neoplasm"[All Fields]) AND "androgen resistant prostatic"[Title/Abstract]) OR (("prostatic neoplasms"[MeSH Terms] OR ("Prostatic"[All Fields] AND "Neoplasms"[All Fields]) OR "prostatic neoplasms"[All Fields] OR ("Prostatic"[All Fields] AND "neoplasm"[All Fields]) OR "prostatic neoplasm"[All Fields]) AND "Androgen-Resistant"[Title/Abstract]) OR (("prostatic neoplasms"[MeSH Terms] OR ("Prostatic"[All Fields] AND "Neoplasms"[All Fields]) OR "prostatic neoplasms"[All Fields]) AND "Androgen-Resistant"[Title/Abstract]) OR "androgen independent prostatic neoplasms"[Title/Abstract] OR "androgen independent prostatic neoplasms"[Title/Abstract] OR "castration resistant prostatic neoplasms"[Title/Abstract] OR "castration resistant prostatic neoplasms"[Title/Abstract] OR "prostatic cancer castration resistant"[Title/Abstract] OR (("cancer s"[All Fields] OR "cancerated"[All Fields] OR "canceration"[All Fields] OR "cancerization"[All Fields] OR "cancerized"[All Fields] OR "cancerous"[All Fields] OR "Neoplasms"[MeSH Terms] OR "Neoplasms"[All Fields] OR "Cancer"[All Fields] OR "Cancers"[All Fields]) AND "castration resistant prostatic"[Title/Abstract]) OR (("cancer s"[All Fields] OR "cancerated"[All Fields] OR "canceration"[All Fields] OR "cancerization"[All Fields] OR "cancerized"[All Fields] OR "cancerous"[All Fields] OR "Neoplasms"[MeSH Terms] OR "Neoplasms"[All Fields] OR "Cancer"[All Fields] OR "Cancers"[All Fields]) AND "castration resistant prostatic"[Title/Abstract]) OR ("Castration-Resistant"[All Fields] AND "prostatic cancers"[Title/Abstract]) OR "prostatic cancer castration resistant"[Title/Abstract] OR (("prostatic neoplasms"[MeSH Terms] OR ("Prostatic"[All Fields] AND "Neoplasms"[All Fields]) OR "prostatic neoplasms"[All Fields] OR ("Prostatic"[All Fields] AND "Cancers"[All Fields]) OR "prostatic cancers"[All Fields]) AND "Castration-Resistant"[Title/Abstract]) OR "androgen insensitive prostatic cancer"[Title/Abstract] OR "androgen insensitive prostatic cancer"[Title/Abstract] OR "androgen resistant prostatic cancer"[Title/Abstract] OR "androgen resistant prostatic cancer"[Title/Abstract] OR (("prostatic neoplasms"[MeSH Terms] OR ("Prostatic"[All Fields] AND "Neoplasms"[All Fields]) OR "prostatic neoplasms"[All Fields] OR ("Prostatic"[All Fields] AND "Cancer"[All Fields]) OR "prostatic cancer"[All Fields]) AND "hormone refractory"[Title/Abstract]) OR "hormone refractory prostatic cancer"[Title/Abstract] OR (("prostat"[All Fields] OR "prostate"[MeSH Terms] OR "prostate"[All Fields] OR "prostates"[All Fields] OR "Prostatic"[All Fields] OR "prostatism"[MeSH Terms] OR "prostatism"[All Fields] OR "prostatitis"[MeSH Terms] OR "prostatitis"[All Fields]) AND "cancer androgen independent"[Title/Abstract]) OR "androgen independent prostatic cancers"[Title/Abstract] OR (("cancer s"[All Fields] OR "cancerated"[All Fields] OR "canceration"[All Fields] OR "cancerization"[All Fields] OR "cancerized"[All Fields] OR "cancerous"[All Fields] OR "Neoplasms"[MeSH Terms] OR "Neoplasms"[All Fields] OR "Cancer"[All Fields] OR "Cancers"[All Fields]) AND "androgen independent prostatic"[Title/Abstract]) OR (("cancer s"[All Fields] OR "cancerated"[All Fields] OR "canceration"[All Fields] OR "cancerization"[All Fields] OR "cancerized"[All Fields] OR "cancerous"[All Fields] OR "Neoplasms"[MeSH Terms] OR "Neoplasms"[All Fields] OR "Cancer"[All Fields] OR "Cancers"[All Fields]) AND "androgen independent prostatic"[Title/Abstract]) OR (("prostat"[All Fields] OR "prostate"[MeSH Terms] OR "prostate"[All Fields] OR "prostates"[All Fields] OR "Prostatic"[All Fields] OR "prostatism"[MeSH Terms] OR "prostatism"[All Fields] OR "prostatitis"[MeSH Terms] OR "prostatitis"[All Fields]) AND "cancer androgen independent"[Title/Abstract]) OR (("prostat"[All Fields] OR "prostate"[MeSH Terms] OR "prostate"[All Fields] OR "prostates"[All Fields] OR "Prostatic"[All Fields] OR "prostatism"[MeSH Terms] OR "prostatism"[All Fields] OR "prostatitis"[MeSH Terms] OR "prostatitis"[All Fields]) AND "cancers androgen independent"[Title/Abstract]) OR (("prostat"[All Fields] OR "prostate"[MeSH Terms] OR "prostate"[All Fields] OR "prostates"[All Fields] OR "Prostatic"[All Fields] OR "prostatism"[MeSH Terms] OR "prostatism"[All Fields] OR "prostatitis"[MeSH Terms] OR "prostatitis"[All Fields]) AND "cancer androgen insensitive"[Title/Abstract]) OR ("Androgen-Insensitive"[All Fields] AND "prostatic cancers"[Title/Abstract]) OR (("cancer s"[All Fields] OR "cancerated"[All Fields] OR "canceration"[All Fields] OR "cancerization"[All Fields] OR "cancerized"[All Fields] OR "cancerous"[All Fields] OR "Neoplasms"[MeSH Terms] OR "Neoplasms"[All Fields] OR "Cancer"[All Fields] OR "Cancers"[All Fields]) AND "androgen insensitive prostatic"[Title/Abstract]) OR (("cancer s"[All Fields] OR "cancerated"[All Fields] OR "canceration"[All Fields] OR "cancerization"[All Fields] OR "cancerized"[All Fields] OR "cancerous"[All Fields] OR "Neoplasms"[MeSH Terms] OR "Neoplasms"[All Fields] OR "Cancer"[All Fields] OR "Cancers"[All Fields]) AND "androgen insensitive prostatic"[Title/Abstract]) OR (("prostat"[All Fields] OR "prostate"[MeSH Terms] OR "prostate"[All Fields] OR "prostates"[All Fields] OR "Prostatic"[All Fields] OR "prostatism"[MeSH Terms] OR "prostatism"[All Fields] OR "prostatitis"[MeSH Terms] OR "prostatitis"[All Fields]) AND "cancer androgen insensitive"[Title/Abstract]) OR (("prostatic neoplasms"[MeSH Terms] OR ("Prostatic"[All Fields] AND "Neoplasms"[All Fields]) OR "prostatic neoplasms"[All Fields] OR ("Prostatic"[All Fields] AND "Cancers"[All Fields]) OR "prostatic cancers"[All Fields]) AND "Androgen-Insensitive"[Title/Abstract]) OR (("prostat"[All Fields] OR "prostate"[MeSH Terms] OR "prostate"[All Fields] OR "prostates"[All Fields] OR "Prostatic"[All Fields] OR "prostatism"[MeSH Terms] OR "prostatism"[All Fields] OR "prostatitis"[MeSH Terms] OR "prostatitis"[All Fields]) AND "cancer androgen resistant"[Title/Abstract]) OR ("Androgen-Resistant"[All Fields] AND "prostatic cancers"[Title/Abstract]) OR (("cancer s"[All Fields] OR "cancerated"[All Fields] OR "canceration"[All Fields] OR "cancerization"[All Fields] OR "cancerized"[All Fields] OR "cancerous"[All Fields] OR "Neoplasms"[MeSH Terms] OR "Neoplasms"[All Fields] OR "Cancer"[All Fields] OR "Cancers"[All Fields]) AND "androgen resistant prostatic"[Title/Abstract]) OR (("cancer s"[All Fields] OR "cancerated"[All Fields] OR "canceration"[All Fields] OR "cancerization"[All Fields] OR "cancerized"[All Fields] OR "cancerous"[All Fields] OR "Neoplasms"[MeSH Terms] OR "Neoplasms"[All Fields] OR "Cancer"[All Fields] OR "Cancers"[All Fields]) AND "androgen resistant prostatic"[Title/Abstract]) OR (("prostat"[All Fields] OR "prostate"[MeSH Terms] OR "prostate"[All Fields] OR "prostates"[All Fields] OR "Prostatic"[All Fields] OR "prostatism"[MeSH Terms] OR "prostatism"[All Fields] OR "prostatitis"[MeSH Terms] OR "prostatitis"[All Fields]) AND "cancer androgen resistant"[Title/Abstract]) OR (("prostatic neoplasms"[MeSH Terms] OR ("Prostatic"[All Fields] AND "Neoplasms"[All Fields]) OR "prostatic neoplasms"[All Fields] OR ("Prostatic"[All Fields] AND "Cancers"[All Fields]) OR "prostatic cancers"[All Fields]) AND "Androgen-Resistant"[Title/Abstract]) OR "androgen independent prostatic cancer"[Title/Abstract] OR "androgen independent prostatic cancer"[Title/Abstract] OR "castration resistant prostatic cancer"[Title/Abstract] OR "castration resistant prostatic cancer"[Title/Abstract] OR "androgen independent pc"[Title/Abstract] OR "androgen independent pca"[Title/Abstract] OR "androgen independent prostate cancer"[Title/Abstract] OR "androgen independent prostatic cancer"[Title/Abstract] OR "androgen insensitive pc"[Title/Abstract] OR "androgen insensitive pca"[Title/Abstract] OR "androgen insensitive prostate cancer"[Title/Abstract] OR "androgen insensitive prostatic cancer"[Title/Abstract] OR ("castrate refractory"[All Fields] AND "pc"[Title/Abstract]) OR ("castrate refractory"[All Fields] AND "pca"[Title/Abstract]) OR "castrate refractory prostate cancer"[Title/Abstract] OR ("castrate refractory"[All Fields] AND "prostatic cancer"[Title/Abstract]) OR "castrate resistant pc"[Title/Abstract] OR "castrate resistant pca"[Title/Abstract] OR "castrate resistant prostate cancer"[Title/Abstract] OR ("castration refractory"[All Fields] AND "pc"[Title/Abstract]) OR ("castration refractory"[All Fields] AND "pca"[Title/Abstract]) OR "castration refractory prostate cancer"[Title/Abstract] OR ("castration refractory"[All Fields] AND "prostatic cancer"[Title/Abstract]) OR "castration resistant pc"[Title/Abstract] OR "castration resistant pca"[Title/Abstract] OR "castration resistant prostatic cancer"[Title/Abstract] OR "castration resistant prostatic neoplasms"[Title/Abstract] OR "crpc"[All Fields]) AND (("castrate"[All Fields] OR "castrated"[All Fields] OR "castrates"[All Fields] OR "castrating"[All Fields] OR "Castration"[MeSH Terms] OR "Castration"[All Fields] OR "castrations"[All Fields] OR "castrator"[All Fields] OR "castrators"[All Fields] OR "orchiectomy"[MeSH Terms] OR "orchiectomy"[All Fields]) AND ("resist"[All Fields] OR "resistance"[All Fields] OR "resistances"[All Fields] OR "Resistant"[All Fields] OR "resistants"[All Fields] OR "resisted"[All Fields] OR "resistence"[All Fields] OR "resistences"[All Fields] OR "resistent"[All Fields] OR "resistibility"[All Fields] OR "resisting"[All Fields] OR "resistive"[All Fields] OR "resistively"[All Fields] OR "resistivities"[All Fields] OR "resistivity"[All Fields] OR "resists"[All Fields]) AND ("prostatic neoplasms"[MeSH Terms] OR ("Prostatic"[All Fields] AND "Neoplasms"[All Fields]) OR "prostatic neoplasms"[All Fields] OR ("prostate"[All Fields] AND "Cancer"[All Fields]) OR "prostate cancer"[All Fields]))) OR "hormone refractory prostate cancer"[Title/Abstract] OR "hormone refractory pc"[Title/Abstract] OR "hormone refractory pca"[Title/Abstract] OR "hormone refractory prostatic cancer"[Title/Abstract] OR ("hormone resistant"[All Fields] AND "pc"[Title/Abstract]) OR "hormone resistant pca"[Title/Abstract] OR "hormone resistant prostate cancer"[Title/Abstract] OR "hormone resistant prostatic cancer"[Title/Abstract] OR "prostatic neoplasms castration resistant"[Title/Abstract]) | 12,325 | 5:23:24 |
| 4 | ‘Antagonists, Androgen’[Title/Abstract] OR ‘Antiandrogens’[Title/Abstract] OR ‘Androgen Antagonist’[Title/Abstract] OR ‘Antagonist, Androgen’[Title/Abstract] OR ‘Antiandrogen’[Title/Abstract] OR ‘Anti-Androgen Effect’[Title/Abstract] OR ‘Anti Androgen Effect’[Title/Abstract] OR ‘Effect, Anti-Androgen’[Title/Abstract] OR ‘Antiandrogen Effect’[Title/Abstract] OR ‘Effect, Antiandrogen’[Title/Abstract] OR ‘Antiandrogen Effects’[Title/Abstract] OR ‘Effects, Antiandrogen’[Title/Abstract] OR ‘Anti-Androgen Effects’[Title/Abstract] OR ‘Anti Androgen Effects’[Title/Abstract] OR ‘Effects, Anti-Androgen’[Title/Abstract] OR ‘androgen antagonist’[Title/Abstract] OR ‘androgen antagonists’[Title/Abstract] OR ‘anti androgen’[Title/Abstract] OR ‘antiandrogen agent’[Title/Abstract] OR ‘antiandrogenic agent’[Title/Abstract] OR ‘antiandrogenic drug’[Title/Abstract] OR ‘antiandrogens’[Title/Abstract] OR ‘nonsteroidal anti androgen’[Title/Abstract] OR ‘nonsteroidal anti androgens’[Title/Abstract] OR ‘nonsteroidal anti-androgen’[Title/Abstract] OR ‘nonsteroidal anti-androgens’[Title/Abstract] OR ‘nonsteroidal antiandrogen’[Title/Abstract] OR ‘nonsteroidal antiandrogens’[Title/Abstract] | "antagonists androgen"[Title/Abstract] OR "antiandrogens"[Title/Abstract] OR "androgen antagonist"[Title/Abstract] OR "antagonist androgen"[Title/Abstract] OR "antiandrogen"[Title/Abstract] OR "anti androgen effect"[Title/Abstract] OR "anti androgen effect"[Title/Abstract] OR (("effect"[All Fields] OR "effecting"[All Fields] OR "effective"[All Fields] OR "effectively"[All Fields] OR "effectiveness"[All Fields] OR "effectivenesses"[All Fields] OR "effectives"[All Fields] OR "effectivities"[All Fields] OR "effectivity"[All Fields] OR "effects"[All Fields]) AND "anti androgen"[Title/Abstract]) OR "antiandrogen effect"[Title/Abstract] OR (("effect"[All Fields] OR "effecting"[All Fields] OR "effective"[All Fields] OR "effectively"[All Fields] OR "effectiveness"[All Fields] OR "effectivenesses"[All Fields] OR "effectives"[All Fields] OR "effectivities"[All Fields] OR "effectivity"[All Fields] OR "effects"[All Fields]) AND "antiandrogen"[Title/Abstract]) OR "antiandrogen effects"[Title/Abstract] OR "effects antiandrogen"[Title/Abstract] OR "anti androgen effects"[Title/Abstract] OR "anti androgen effects"[Title/Abstract] OR (("effect"[All Fields] OR "effecting"[All Fields] OR "effective"[All Fields] OR "effectively"[All Fields] OR "effectiveness"[All Fields] OR "effectivenesses"[All Fields] OR "effectives"[All Fields] OR "effectivities"[All Fields] OR "effectivity"[All Fields] OR "effects"[All Fields]) AND "anti androgen"[Title/Abstract]) OR "androgen antagonist"[Title/Abstract] OR "androgen antagonists"[Title/Abstract] OR "anti androgen"[Title/Abstract] OR "antiandrogen agent"[Title/Abstract] OR "antiandrogenic agent"[Title/Abstract] OR "antiandrogenic drug"[Title/Abstract] OR "antiandrogens"[Title/Abstract] OR "nonsteroidal anti androgen"[Title/Abstract] OR "nonsteroidal anti androgens"[Title/Abstract] OR "nonsteroidal anti androgen"[Title/Abstract] OR "nonsteroidal anti androgens"[Title/Abstract] OR "nonsteroidal antiandrogen"[Title/Abstract] OR "nonsteroidal antiandrogens"[Title/Abstract] | 7,117 | 5:23:08 |
| 3 | Androgen Antagonists[MeSH Terms] | "androgen antagonists"[MeSH Terms] | 14,020 | 5:22:54 |
| 2 | ‘Castration-Resistant Prostatic Neoplasm’[Title/Abstract] OR ‘Neoplasm, Castration-Resistant Prostatic’[Title/Abstract] OR ‘Neoplasms, Castration-Resistant Prostatic’[Title/Abstract] OR ‘Prostatic Neoplasm, Castration-Resistant’[Title/Abstract] OR ‘Prostatic Neoplasms, Castration Resistant’[Title/Abstract] OR ‘Androgen-Insensitive Prostatic Neoplasms’[Title/Abstract] OR ‘Androgen Insensitive Prostatic Neoplasms’[Title/Abstract] OR ‘Androgen-Resistant Prostatic Neoplasms’[Title/Abstract] OR ‘Androgen Resistant Prostatic Neoplasms’[Title/Abstract] OR ‘Prostatic Neoplasms, Hormone Refractory’[Title/Abstract] OR ‘Hormone Refractory Prostatic Neoplasms’[Title/Abstract] OR ‘Prostatic Neoplasms, Androgen-Independent’[Title/Abstract] OR ‘Androgen-Independent Prostatic Neoplasm’[Title/Abstract] OR ‘Neoplasm, Androgen-Independent Prostatic’[Title/Abstract] OR ‘Neoplasms, Androgen-Independent Prostatic’[Title/Abstract] OR ‘Prostatic Neoplasm, Androgen-Independent’[Title/Abstract] OR ‘Prostatic Neoplasms, Androgen Independent’[Title/Abstract] OR ‘Prostatic Neoplasms, Androgen-Insensitive’[Title/Abstract] OR ‘Androgen-Insensitive Prostatic Neoplasm’[Title/Abstract] OR ‘Neoplasm, Androgen-Insensitive Prostatic’[Title/Abstract] OR ‘Neoplasms, Androgen-Insensitive Prostatic’[Title/Abstract] OR ‘Prostatic Neoplasm, Androgen-Insensitive’[Title/Abstract] OR ‘Prostatic Neoplasms, Androgen Insensitive’[Title/Abstract] OR ‘Prostatic Neoplasms, Androgen-Resistant’[Title/Abstract] OR ‘Androgen-Resistant Prostatic Neoplasm’[Title/Abstract] OR ‘Neoplasm, Androgen-Resistant Prostatic’[Title/Abstract] OR ‘Neoplasms, Androgen-Resistant Prostatic’[Title/Abstract] OR ‘Prostatic Neoplasm, Androgen-Resistant’[Title/Abstract] OR ‘Prostatic Neoplasms, Androgen Resistant’[Title/Abstract] OR ‘Androgen-Independent Prostatic Neoplasms’[Title/Abstract] OR ‘Androgen Independent Prostatic Neoplasms’[Title/Abstract] OR ‘Castration-Resistant Prostatic Neoplasms’[Title/Abstract] OR ‘Castration Resistant Prostatic Neoplasms’[Title/Abstract] OR ‘Prostatic Cancer, Castration-Resistant’[Title/Abstract] OR ‘Cancer, Castration-Resistant Prostatic’[Title/Abstract] OR ‘Cancers, Castration-Resistant Prostatic’[Title/Abstract] OR ‘Castration-Resistant Prostatic Cancers’[Title/Abstract] OR ‘Prostatic Cancer, Castration Resistant’[Title/Abstract] OR ‘Prostatic Cancers, Castration-Resistant’[Title/Abstract] OR ‘Androgen-Insensitive Prostatic Cancer’[Title/Abstract] OR ‘Androgen Insensitive Prostatic Cancer’[Title/Abstract] OR ‘Androgen-Resistant Prostatic Cancer’[Title/Abstract] OR ‘Androgen Resistant Prostatic Cancer’[Title/Abstract] OR ‘Prostatic Cancer, Hormone Refractory’[Title/Abstract] OR ‘Hormone Refractory Prostatic Cancer’[Title/Abstract] OR ‘Prostatic Cancer, Androgen-Independent’[Title/Abstract] OR ‘Androgen-Independent Prostatic Cancers’[Title/Abstract] OR ‘Cancer, Androgen-Independent Prostatic’[Title/Abstract] OR ‘Cancers, Androgen-Independent Prostatic’[Title/Abstract] OR ‘Prostatic Cancer, Androgen Independent’[Title/Abstract] OR ‘Prostatic Cancers, Androgen-Independent’[Title/Abstract] OR ‘Prostatic Cancer, Androgen-Insensitive’[Title/Abstract] OR ‘Androgen-Insensitive Prostatic Cancers’[Title/Abstract] OR ‘Cancer, Androgen-Insensitive Prostatic’[Title/Abstract] OR ‘Cancers, Androgen-Insensitive Prostatic’[Title/Abstract] OR ‘Prostatic Cancer, Androgen Insensitive’[Title/Abstract] OR ‘Prostatic Cancers, Androgen-Insensitive’[Title/Abstract] OR ‘Prostatic Cancer, Androgen-Resistant’[Title/Abstract] OR ‘Androgen-Resistant Prostatic Cancers’[Title/Abstract] OR ‘Cancer, Androgen-Resistant Prostatic’[Title/Abstract] OR ‘Cancers, Androgen-Resistant Prostatic’[Title/Abstract] OR ‘Prostatic Cancer, Androgen Resistant’[Title/Abstract] OR ‘Prostatic Cancers, Androgen-Resistant’[Title/Abstract] OR ‘Androgen-Independent Prostatic Cancer’[Title/Abstract] OR ‘Androgen Independent Prostatic Cancer’[Title/Abstract] OR ‘Castration-Resistant Prostatic Cancer’[Title/Abstract] OR ‘Castration Resistant Prostatic Cancer’[Title/Abstract] OR ‘androgen-independent PC’[Title/Abstract] OR ‘androgen-independent PCa’[Title/Abstract] OR ‘androgen-independent prostate cancer’[Title/Abstract] OR ‘androgen-independent prostatic cancer’[Title/Abstract] OR ‘androgen-insensitive PC’[Title/Abstract] OR ‘androgen-insensitive PCa’[Title/Abstract] OR ‘androgen-insensitive prostate cancer’[Title/Abstract] OR ‘androgen-insensitive prostatic cancer’[Title/Abstract] OR ‘castrate-refractory PC’[Title/Abstract] OR ‘castrate-refractory PCa’[Title/Abstract] OR ‘castrate-refractory prostate cancer’[Title/Abstract] OR ‘castrate-refractory prostatic cancer’[Title/Abstract] OR ‘castrate-resistant PC’[Title/Abstract] OR ‘castrate-resistant PCa’[Title/Abstract] OR ‘castrate-resistant prostate cancer’[Title/Abstract] OR ‘castration-refractory PC’[Title/Abstract] OR ‘castration-refractory PCa’[Title/Abstract] OR ‘castration-refractory prostate cancer’[Title/Abstract] OR ‘castration-refractory prostatic cancer’[Title/Abstract] OR ‘castration-resistant PC’[Title/Abstract] OR ‘castration-resistant PCa’[Title/Abstract] OR ‘castration-resistant prostatic cancer’[Title/Abstract] OR ‘castration-resistant prostatic neoplasms’[Title/Abstract] OR ‘CRPC (castration resistant prostate cancer)’[Title/Abstract] OR ‘hormone refractory prostate cancer’[Title/Abstract] OR ‘hormone-refractory PC’[Title/Abstract] OR ‘hormone-refractory PCa’[Title/Abstract] OR ‘hormone-refractory prostatic cancer’[Title/Abstract] OR ‘hormone-resistant PC’[Title/Abstract] OR ‘hormone-resistant PCa’[Title/Abstract] OR ‘hormone-resistant prostate cancer’[Title/Abstract] OR ‘hormone-resistant prostatic cancer’[Title/Abstract] OR ‘prostatic neoplasms, castration-resistant’[Title/Abstract] | "castration resistant prostatic neoplasm"[Title/Abstract] OR (("neoplasm s"[All Fields] OR "Neoplasms"[MeSH Terms] OR "Neoplasms"[All Fields] OR "neoplasm"[All Fields]) AND "castration resistant prostatic"[Title/Abstract]) OR (("neoplasm s"[All Fields] OR "Neoplasms"[MeSH Terms] OR "Neoplasms"[All Fields] OR "neoplasm"[All Fields]) AND "castration resistant prostatic"[Title/Abstract]) OR (("prostatic neoplasms"[MeSH Terms] OR ("Prostatic"[All Fields] AND "Neoplasms"[All Fields]) OR "prostatic neoplasms"[All Fields] OR ("Prostatic"[All Fields] AND "neoplasm"[All Fields]) OR "prostatic neoplasm"[All Fields]) AND "Castration-Resistant"[Title/Abstract]) OR "prostatic neoplasms castration resistant"[Title/Abstract] OR ("Androgen-Insensitive"[All Fields] AND "prostatic neoplasms"[Title/Abstract]) OR ((("androgen s"[All Fields] OR "androgene"[All Fields] OR "androgenes"[All Fields] OR "androgenic"[All Fields] OR "androgenicity"[All Fields] OR "androgenized"[All Fields] OR "androgenizing"[All Fields] OR "androgenous"[All Fields] OR "androgens"[Pharmacological Action] OR "androgens"[MeSH Terms] OR "androgens"[All Fields] OR "Androgen"[All Fields] OR "virilism"[MeSH Terms] OR "virilism"[All Fields] OR "androgenization"[All Fields]) AND ("Insensitive"[All Fields] OR "insensitivities"[All Fields] OR "insensitivity"[All Fields])) AND "prostatic neoplasms"[Title/Abstract]) OR ("Androgen-Resistant"[All Fields] AND "prostatic neoplasms"[Title/Abstract]) OR (("androgen s"[All Fields] OR "androgene"[All Fields] OR "androgenes"[All Fields] OR "androgenic"[All Fields] OR "androgenicity"[All Fields] OR "androgenized"[All Fields] OR "androgenizing"[All Fields] OR "androgenous"[All Fields] OR "androgens"[Pharmacological Action] OR "androgens"[MeSH Terms] OR "androgens"[All Fields] OR "Androgen"[All Fields] OR "virilism"[MeSH Terms] OR "virilism"[All Fields] OR "androgenization"[All Fields]) AND "resistant prostatic neoplasms"[Title/Abstract]) OR (("prostatic neoplasms"[MeSH Terms] OR ("Prostatic"[All Fields] AND "Neoplasms"[All Fields]) OR "prostatic neoplasms"[All Fields]) AND "hormone refractory"[Title/Abstract]) OR ((("hormon"[All Fields] OR "hormonal"[All Fields] OR "hormonally"[All Fields] OR "hormonals"[All Fields] OR "hormone s"[All Fields] OR "hormones"[Pharmacological Action] OR "hormones"[MeSH Terms] OR "hormones"[All Fields] OR "Hormone"[All Fields] OR "hormons"[All Fields]) AND ("refractories"[All Fields] OR "refractoriness"[All Fields] OR "Refractory"[All Fields])) AND "prostatic neoplasms"[Title/Abstract]) OR (("prostatic neoplasms"[MeSH Terms] OR ("Prostatic"[All Fields] AND "Neoplasms"[All Fields]) OR "prostatic neoplasms"[All Fields]) AND "Androgen-Independent"[Title/Abstract]) OR ("Androgen-Independent"[All Fields] AND "prostatic neoplasm"[Title/Abstract]) OR (("neoplasm s"[All Fields] OR "Neoplasms"[MeSH Terms] OR "Neoplasms"[All Fields] OR "neoplasm"[All Fields]) AND "androgen independent prostatic"[Title/Abstract]) OR (("neoplasm s"[All Fields] OR "Neoplasms"[MeSH Terms] OR "Neoplasms"[All Fields] OR "neoplasm"[All Fields]) AND "androgen independent prostatic"[Title/Abstract]) OR (("prostatic neoplasms"[MeSH Terms] OR ("Prostatic"[All Fields] AND "Neoplasms"[All Fields]) OR "prostatic neoplasms"[All Fields] OR ("Prostatic"[All Fields] AND "neoplasm"[All Fields]) OR "prostatic neoplasm"[All Fields]) AND "Androgen-Independent"[Title/Abstract]) OR (("prostatic neoplasms"[MeSH Terms] OR ("Prostatic"[All Fields] AND "Neoplasms"[All Fields]) OR "prostatic neoplasms"[All Fields]) AND "Androgen-Independent"[Title/Abstract]) OR (("prostatic neoplasms"[MeSH Terms] OR ("Prostatic"[All Fields] AND "Neoplasms"[All Fields]) OR "prostatic neoplasms"[All Fields]) AND "Androgen-Insensitive"[Title/Abstract]) OR ("Androgen-Insensitive"[All Fields] AND "prostatic neoplasm"[Title/Abstract]) OR (("neoplasm s"[All Fields] OR "Neoplasms"[MeSH Terms] OR "Neoplasms"[All Fields] OR "neoplasm"[All Fields]) AND "androgen insensitive prostatic"[Title/Abstract]) OR (("neoplasm s"[All Fields] OR "Neoplasms"[MeSH Terms] OR "Neoplasms"[All Fields] OR "neoplasm"[All Fields]) AND "androgen insensitive prostatic"[Title/Abstract]) OR (("prostatic neoplasms"[MeSH Terms] OR ("Prostatic"[All Fields] AND "Neoplasms"[All Fields]) OR "prostatic neoplasms"[All Fields] OR ("Prostatic"[All Fields] AND "neoplasm"[All Fields]) OR "prostatic neoplasm"[All Fields]) AND "Androgen-Insensitive"[Title/Abstract]) OR (("prostatic neoplasms"[MeSH Terms] OR ("Prostatic"[All Fields] AND "Neoplasms"[All Fields]) OR "prostatic neoplasms"[All Fields]) AND "Androgen-Insensitive"[Title/Abstract]) OR (("prostatic neoplasms"[MeSH Terms] OR ("Prostatic"[All Fields] AND "Neoplasms"[All Fields]) OR "prostatic neoplasms"[All Fields]) AND "Androgen-Resistant"[Title/Abstract]) OR ("Androgen-Resistant"[All Fields] AND "prostatic neoplasm"[Title/Abstract]) OR (("neoplasm s"[All Fields] OR "Neoplasms"[MeSH Terms] OR "Neoplasms"[All Fields] OR "neoplasm"[All Fields]) AND "androgen resistant prostatic"[Title/Abstract]) OR (("neoplasm s"[All Fields] OR "Neoplasms"[MeSH Terms] OR "Neoplasms"[All Fields] OR "neoplasm"[All Fields]) AND "androgen resistant prostatic"[Title/Abstract]) OR (("prostatic neoplasms"[MeSH Terms] OR ("Prostatic"[All Fields] AND "Neoplasms"[All Fields]) OR "prostatic neoplasms"[All Fields] OR ("Prostatic"[All Fields] AND "neoplasm"[All Fields]) OR "prostatic neoplasm"[All Fields]) AND "Androgen-Resistant"[Title/Abstract]) OR (("prostatic neoplasms"[MeSH Terms] OR ("Prostatic"[All Fields] AND "Neoplasms"[All Fields]) OR "prostatic neoplasms"[All Fields]) AND "Androgen-Resistant"[Title/Abstract]) OR "androgen independent prostatic neoplasms"[Title/Abstract] OR "androgen independent prostatic neoplasms"[Title/Abstract] OR "castration resistant prostatic neoplasms"[Title/Abstract] OR "castration resistant prostatic neoplasms"[Title/Abstract] OR "prostatic cancer castration resistant"[Title/Abstract] OR (("cancer s"[All Fields] OR "cancerated"[All Fields] OR "canceration"[All Fields] OR "cancerization"[All Fields] OR "cancerized"[All Fields] OR "cancerous"[All Fields] OR "Neoplasms"[MeSH Terms] OR "Neoplasms"[All Fields] OR "Cancer"[All Fields] OR "Cancers"[All Fields]) AND "castration resistant prostatic"[Title/Abstract]) OR (("cancer s"[All Fields] OR "cancerated"[All Fields] OR "canceration"[All Fields] OR "cancerization"[All Fields] OR "cancerized"[All Fields] OR "cancerous"[All Fields] OR "Neoplasms"[MeSH Terms] OR "Neoplasms"[All Fields] OR "Cancer"[All Fields] OR "Cancers"[All Fields]) AND "castration resistant prostatic"[Title/Abstract]) OR ("Castration-Resistant"[All Fields] AND "prostatic cancers"[Title/Abstract]) OR "prostatic cancer castration resistant"[Title/Abstract] OR (("prostatic neoplasms"[MeSH Terms] OR ("Prostatic"[All Fields] AND "Neoplasms"[All Fields]) OR "prostatic neoplasms"[All Fields] OR ("Prostatic"[All Fields] AND "Cancers"[All Fields]) OR "prostatic cancers"[All Fields]) AND "Castration-Resistant"[Title/Abstract]) OR "androgen insensitive prostatic cancer"[Title/Abstract] OR "androgen insensitive prostatic cancer"[Title/Abstract] OR "androgen resistant prostatic cancer"[Title/Abstract] OR "androgen resistant prostatic cancer"[Title/Abstract] OR (("prostatic neoplasms"[MeSH Terms] OR ("Prostatic"[All Fields] AND "Neoplasms"[All Fields]) OR "prostatic neoplasms"[All Fields] OR ("Prostatic"[All Fields] AND "Cancer"[All Fields]) OR "prostatic cancer"[All Fields]) AND "hormone refractory"[Title/Abstract]) OR "hormone refractory prostatic cancer"[Title/Abstract] OR (("prostat"[All Fields] OR "prostate"[MeSH Terms] OR "prostate"[All Fields] OR "prostates"[All Fields] OR "Prostatic"[All Fields] OR "prostatism"[MeSH Terms] OR "prostatism"[All Fields] OR "prostatitis"[MeSH Terms] OR "prostatitis"[All Fields]) AND "cancer androgen independent"[Title/Abstract]) OR "androgen independent prostatic cancers"[Title/Abstract] OR (("cancer s"[All Fields] OR "cancerated"[All Fields] OR "canceration"[All Fields] OR "cancerization"[All Fields] OR "cancerized"[All Fields] OR "cancerous"[All Fields] OR "Neoplasms"[MeSH Terms] OR "Neoplasms"[All Fields] OR "Cancer"[All Fields] OR "Cancers"[All Fields]) AND "androgen independent prostatic"[Title/Abstract]) OR (("cancer s"[All Fields] OR "cancerated"[All Fields] OR "canceration"[All Fields] OR "cancerization"[All Fields] OR "cancerized"[All Fields] OR "cancerous"[All Fields] OR "Neoplasms"[MeSH Terms] OR "Neoplasms"[All Fields] OR "Cancer"[All Fields] OR "Cancers"[All Fields]) AND "androgen independent prostatic"[Title/Abstract]) OR (("prostat"[All Fields] OR "prostate"[MeSH Terms] OR "prostate"[All Fields] OR "prostates"[All Fields] OR "Prostatic"[All Fields] OR "prostatism"[MeSH Terms] OR "prostatism"[All Fields] OR "prostatitis"[MeSH Terms] OR "prostatitis"[All Fields]) AND "cancer androgen independent"[Title/Abstract]) OR (("prostat"[All Fields] OR "prostate"[MeSH Terms] OR "prostate"[All Fields] OR "prostates"[All Fields] OR "Prostatic"[All Fields] OR "prostatism"[MeSH Terms] OR "prostatism"[All Fields] OR "prostatitis"[MeSH Terms] OR "prostatitis"[All Fields]) AND "cancers androgen independent"[Title/Abstract]) OR (("prostat"[All Fields] OR "prostate"[MeSH Terms] OR "prostate"[All Fields] OR "prostates"[All Fields] OR "Prostatic"[All Fields] OR "prostatism"[MeSH Terms] OR "prostatism"[All Fields] OR "prostatitis"[MeSH Terms] OR "prostatitis"[All Fields]) AND "cancer androgen insensitive"[Title/Abstract]) OR ("Androgen-Insensitive"[All Fields] AND "prostatic cancers"[Title/Abstract]) OR (("cancer s"[All Fields] OR "cancerated"[All Fields] OR "canceration"[All Fields] OR "cancerization"[All Fields] OR "cancerized"[All Fields] OR "cancerous"[All Fields] OR "Neoplasms"[MeSH Terms] OR "Neoplasms"[All Fields] OR "Cancer"[All Fields] OR "Cancers"[All Fields]) AND "androgen insensitive prostatic"[Title/Abstract]) OR (("cancer s"[All Fields] OR "cancerated"[All Fields] OR "canceration"[All Fields] OR "cancerization"[All Fields] OR "cancerized"[All Fields] OR "cancerous"[All Fields] OR "Neoplasms"[MeSH Terms] OR "Neoplasms"[All Fields] OR "Cancer"[All Fields] OR "Cancers"[All Fields]) AND "androgen insensitive prostatic"[Title/Abstract]) OR (("prostat"[All Fields] OR "prostate"[MeSH Terms] OR "prostate"[All Fields] OR "prostates"[All Fields] OR "Prostatic"[All Fields] OR "prostatism"[MeSH Terms] OR "prostatism"[All Fields] OR "prostatitis"[MeSH Terms] OR "prostatitis"[All Fields]) AND "cancer androgen insensitive"[Title/Abstract]) OR (("prostatic neoplasms"[MeSH Terms] OR ("Prostatic"[All Fields] AND "Neoplasms"[All Fields]) OR "prostatic neoplasms"[All Fields] OR ("Prostatic"[All Fields] AND "Cancers"[All Fields]) OR "prostatic cancers"[All Fields]) AND "Androgen-Insensitive"[Title/Abstract]) OR (("prostat"[All Fields] OR "prostate"[MeSH Terms] OR "prostate"[All Fields] OR "prostates"[All Fields] OR "Prostatic"[All Fields] OR "prostatism"[MeSH Terms] OR "prostatism"[All Fields] OR "prostatitis"[MeSH Terms] OR "prostatitis"[All Fields]) AND "cancer androgen resistant"[Title/Abstract]) OR ("Androgen-Resistant"[All Fields] AND "prostatic cancers"[Title/Abstract]) OR (("cancer s"[All Fields] OR "cancerated"[All Fields] OR "canceration"[All Fields] OR "cancerization"[All Fields] OR "cancerized"[All Fields] OR "cancerous"[All Fields] OR "Neoplasms"[MeSH Terms] OR "Neoplasms"[All Fields] OR "Cancer"[All Fields] OR "Cancers"[All Fields]) AND "androgen resistant prostatic"[Title/Abstract]) OR (("cancer s"[All Fields] OR "cancerated"[All Fields] OR "canceration"[All Fields] OR "cancerization"[All Fields] OR "cancerized"[All Fields] OR "cancerous"[All Fields] OR "Neoplasms"[MeSH Terms] OR "Neoplasms"[All Fields] OR "Cancer"[All Fields] OR "Cancers"[All Fields]) AND "androgen resistant prostatic"[Title/Abstract]) OR (("prostat"[All Fields] OR "prostate"[MeSH Terms] OR "prostate"[All Fields] OR "prostates"[All Fields] OR "Prostatic"[All Fields] OR "prostatism"[MeSH Terms] OR "prostatism"[All Fields] OR "prostatitis"[MeSH Terms] OR "prostatitis"[All Fields]) AND "cancer androgen resistant"[Title/Abstract]) OR (("prostatic neoplasms"[MeSH Terms] OR ("Prostatic"[All Fields] AND "Neoplasms"[All Fields]) OR "prostatic neoplasms"[All Fields] OR ("Prostatic"[All Fields] AND "Cancers"[All Fields]) OR "prostatic cancers"[All Fields]) AND "Androgen-Resistant"[Title/Abstract]) OR "androgen independent prostatic cancer"[Title/Abstract] OR "androgen independent prostatic cancer"[Title/Abstract] OR "castration resistant prostatic cancer"[Title/Abstract] OR "castration resistant prostatic cancer"[Title/Abstract] OR "androgen independent pc"[Title/Abstract] OR "androgen independent pca"[Title/Abstract] OR "androgen independent prostate cancer"[Title/Abstract] OR "androgen independent prostatic cancer"[Title/Abstract] OR "androgen insensitive pc"[Title/Abstract] OR "androgen insensitive pca"[Title/Abstract] OR "androgen insensitive prostate cancer"[Title/Abstract] OR "androgen insensitive prostatic cancer"[Title/Abstract] OR ("castrate refractory"[All Fields] AND "pc"[Title/Abstract]) OR ("castrate refractory"[All Fields] AND "pca"[Title/Abstract]) OR "castrate refractory prostate cancer"[Title/Abstract] OR ("castrate refractory"[All Fields] AND "prostatic cancer"[Title/Abstract]) OR "castrate resistant pc"[Title/Abstract] OR "castrate resistant pca"[Title/Abstract] OR "castrate resistant prostate cancer"[Title/Abstract] OR ("castration refractory"[All Fields] AND "pc"[Title/Abstract]) OR ("castration refractory"[All Fields] AND "pca"[Title/Abstract]) OR "castration refractory prostate cancer"[Title/Abstract] OR ("castration refractory"[All Fields] AND "prostatic cancer"[Title/Abstract]) OR "castration resistant pc"[Title/Abstract] OR "castration resistant pca"[Title/Abstract] OR "castration resistant prostatic cancer"[Title/Abstract] OR "castration resistant prostatic neoplasms"[Title/Abstract] OR "crpc castration resistant prostate cancer"[Title/Abstract] OR "hormone refractory prostate cancer"[Title/Abstract] OR "hormone refractory pc"[Title/Abstract] OR "hormone refractory pca"[Title/Abstract] OR "hormone refractory prostatic cancer"[Title/Abstract] OR ("hormone resistant"[All Fields] AND "pc"[Title/Abstract]) OR "hormone resistant pca"[Title/Abstract] OR "hormone resistant prostate cancer"[Title/Abstract] OR "hormone resistant prostatic cancer"[Title/Abstract] OR "prostatic neoplasms castration resistant"[Title/Abstract] | 14,582 | 5:22:35 |
| 1 | Prostatic Neoplasms, Castration-Resistant[MeSH Terms] | "prostatic neoplasms, castration resistant"[MeSH Terms] | 5,813 | 5:22:11 |

| Embase |  |  |  |
| --- | --- | --- | --- |
| No. | Query | Results | Date |
| #9 | #7 AND #8 | 1438 | 17-Oct-22 |
| #8 | 'random*':ab,ti,kw | 1852117 | 17-Oct-22 |
| #7 | #5 AND #6 | 10158 | 17-Oct-22 |
| #6 | #3 OR #4 | 78303 | 17-Oct-22 |
| #5 | #1 OR #2 | 26662 | 17-Oct-22 |
| #4 | 'antagonists, androgen':ab,ti,kw OR 'antagonist, androgen':ab,ti,kw OR 'antiandrogen':ab,ti,kw OR 'anti-androgen effect':ab,ti,kw OR 'anti androgen effect':ab,ti,kw OR 'effect, anti-androgen':ab,ti,kw OR 'antiandrogen effect':ab,ti,kw OR 'effect, antiandrogen':ab,ti,kw OR 'antiandrogen effects':ab,ti,kw OR 'effects, antiandrogen':ab,ti,kw OR 'anti-androgen effects':ab,ti,kw OR 'anti androgen effects':ab,ti,kw OR 'effects, anti-androgen':ab,ti,kw OR 'androgen antagonist':ab,ti,kw OR 'androgen antagonists':ab,ti,kw OR 'anti androgen':ab,ti,kw OR 'antiandrogen agent':ab,ti,kw OR 'antiandrogenic agent':ab,ti,kw OR 'antiandrogenic drug':ab,ti,kw OR 'antiandrogens':ab,ti,kw OR 'nonsteroidal anti androgen':ab,ti,kw OR 'nonsteroidal anti androgens':ab,ti,kw OR 'nonsteroidal anti-androgen':ab,ti,kw OR 'nonsteroidal anti-androgens':ab,ti,kw OR 'nonsteroidal antiandrogen':ab,ti,kw OR 'nonsteroidal antiandrogens':ab,ti,kw | 10206 | 17-Oct-22 |
| #3 | 'antiandrogen'/exp | 76087 | 17-Oct-22 |
| #2 | 'castration-resistant prostatic neoplasm':ab,ti,kw OR 'neoplasm, castration-resistant prostatic':ab,ti,kw OR 'neoplasms, castration-resistant prostatic':ab,ti,kw OR 'prostatic neoplasm, castration-resistant':ab,ti,kw OR 'prostatic neoplasms, castration resistant':ab,ti,kw OR 'androgen-insensitive prostatic neoplasms':ab,ti,kw OR 'androgen insensitive prostatic neoplasms':ab,ti,kw OR 'androgen-resistant prostatic neoplasms':ab,ti,kw OR 'androgen resistant prostatic neoplasms':ab,ti,kw OR 'prostatic neoplasms, hormone refractory':ab,ti,kw OR 'hormone refractory prostatic neoplasms':ab,ti,kw OR 'prostatic neoplasms, androgen-independent':ab,ti,kw OR 'androgen-independent prostatic neoplasm':ab,ti,kw OR 'neoplasm, androgen-independent prostatic':ab,ti,kw OR 'neoplasms, androgen-independent prostatic':ab,ti,kw OR 'prostatic neoplasm, androgen-independent':ab,ti,kw OR 'prostatic neoplasms, androgen independent':ab,ti,kw OR 'prostatic neoplasms, androgen-insensitive':ab,ti,kw OR 'androgen-insensitive prostatic neoplasm':ab,ti,kw OR 'neoplasm, androgen-insensitive prostatic':ab,ti,kw OR 'neoplasms, androgen-insensitive prostatic':ab,ti,kw OR 'prostatic neoplasm, androgen-insensitive':ab,ti,kw OR 'prostatic neoplasms, androgen insensitive':ab,ti,kw OR 'prostatic neoplasms, androgen-resistant':ab,ti,kw OR 'androgen-resistant prostatic neoplasm':ab,ti,kw OR 'neoplasm, androgen-resistant prostatic':ab,ti,kw OR 'neoplasms, androgen-resistant prostatic':ab,ti,kw OR 'prostatic neoplasm, androgen-resistant':ab,ti,kw OR 'prostatic neoplasms, androgen resistant':ab,ti,kw OR 'androgen-independent prostatic neoplasms':ab,ti,kw OR 'androgen independent prostatic neoplasms':ab,ti,kw OR 'castration resistant prostatic neoplasms':ab,ti,kw OR 'prostatic cancer, castration-resistant':ab,ti,kw OR 'cancer, castration-resistant prostatic':ab,ti,kw OR 'cancers, castration-resistant prostatic':ab,ti,kw OR 'castration-resistant prostatic cancers':ab,ti,kw OR 'prostatic cancer, castration resistant':ab,ti,kw OR 'prostatic cancers, castration-resistant':ab,ti,kw OR 'androgen insensitive prostatic cancer':ab,ti,kw OR 'androgen-resistant prostatic cancer':ab,ti,kw OR 'androgen resistant prostatic cancer':ab,ti,kw OR 'prostatic cancer, hormone refractory':ab,ti,kw OR 'hormone refractory prostatic cancer':ab,ti,kw OR 'prostatic cancer, androgen-independent':ab,ti,kw OR 'androgen-independent prostatic cancers':ab,ti,kw OR 'cancer, androgen-independent prostatic':ab,ti,kw OR 'cancers, androgen-independent prostatic':ab,ti,kw OR 'prostatic cancer, androgen independent':ab,ti,kw OR 'prostatic cancers, androgen-independent':ab,ti,kw OR 'prostatic cancer, androgen-insensitive':ab,ti,kw OR 'androgen-insensitive prostatic cancers':ab,ti,kw OR 'cancer, androgen-insensitive prostatic':ab,ti,kw OR 'cancers, androgen-insensitive prostatic':ab,ti,kw OR 'prostatic cancer, androgen insensitive':ab,ti,kw OR 'prostatic cancers, androgen-insensitive':ab,ti,kw OR 'prostatic cancer, androgen-resistant':ab,ti,kw OR 'androgen-resistant prostatic cancers':ab,ti,kw OR 'cancer, androgen-resistant prostatic':ab,ti,kw OR 'cancers, androgen-resistant prostatic':ab,ti,kw OR 'prostatic cancer, androgen resistant':ab,ti,kw OR 'prostatic cancers, androgen-resistant':ab,ti,kw OR 'androgen independent prostatic cancer':ab,ti,kw OR 'castration resistant prostatic cancer':ab,ti,kw OR 'androgen-independent pc':ab,ti,kw OR 'androgen-independent pca':ab,ti,kw OR 'androgen-independent prostate cancer':ab,ti,kw OR 'androgen-independent prostatic cancer':ab,ti,kw OR 'androgen-insensitive pc':ab,ti,kw OR 'androgen-insensitive pca':ab,ti,kw OR 'androgen-insensitive prostate cancer':ab,ti,kw OR 'androgen-insensitive prostatic cancer':ab,ti,kw OR 'castrate-refractory pc':ab,ti,kw OR 'castrate-refractory pca':ab,ti,kw OR 'castrate-refractory prostate cancer':ab,ti,kw OR 'castrate-refractory prostatic cancer':ab,ti,kw OR 'castrate-resistant pc':ab,ti,kw OR 'castrate-resistant pca':ab,ti,kw OR 'castrate-resistant prostate cancer':ab,ti,kw OR 'castration-refractory pc':ab,ti,kw OR 'castration-refractory pca':ab,ti,kw OR 'castration-refractory prostate cancer':ab,ti,kw OR 'castration-refractory prostatic cancer':ab,ti,kw OR 'castration-resistant pc':ab,ti,kw OR 'castration-resistant pca':ab,ti,kw OR 'castration-resistant prostatic cancer':ab,ti,kw OR 'castration-resistant prostatic neoplasms':ab,ti,kw OR 'crpc (castration resistant prostate cancer)':ab,ti,kw OR 'hormone refractory prostate cancer':ab,ti,kw OR 'hormone-refractory pc':ab,ti,kw OR 'hormone-refractory pca':ab,ti,kw OR 'hormone-refractory prostatic cancer':ab,ti,kw OR 'hormone-resistant pc':ab,ti,kw OR 'hormone-resistant pca':ab,ti,kw OR 'hormone-resistant prostate cancer':ab,ti,kw OR 'hormone-resistant prostatic cancer':ab,ti,kw OR 'prostatic neoplasms, castration-resistant':ab,ti,kw | 8841 | 17-Oct-22 |
| #1 | 'castration resistant prostate cancer'/exp OR 'castration resistant prostate cancer' | 20418 | 17-Oct-22 |

| Cochrane Library | | |
| --- | --- | --- |
| Date Run: 17/10/2022 04:32:27 | | |
| ID | Search | Hits |
| #1 | MeSH descriptor: [Prostatic Neoplasms, Castration-Resistant] explode all trees | 352 |
| #2 | (‘Castration-Resistant Prostatic Neoplasm’ OR ‘Neoplasm, Castration-Resistant Prostatic’ OR ‘Neoplasms, Castration-Resistant Prostatic’ OR ‘Prostatic Neoplasm, Castration-Resistant’ OR ‘Prostatic Neoplasms, Castration Resistant’ OR ‘Androgen-Insensitive Prostatic Neoplasms’ OR ‘Androgen Insensitive Prostatic Neoplasms’ OR ‘Androgen-Resistant Prostatic Neoplasms’ OR ‘Androgen Resistant Prostatic Neoplasms’ OR ‘Prostatic Neoplasms, Hormone Refractory’ OR ‘Hormone Refractory Prostatic Neoplasms’ OR ‘Prostatic Neoplasms, Androgen-Independent’ OR ‘Androgen-Independent Prostatic Neoplasm’ OR ‘Neoplasm, Androgen-Independent Prostatic’ OR ‘Neoplasms, Androgen-Independent Prostatic’ OR ‘Prostatic Neoplasm, Androgen-Independent’ OR ‘Prostatic Neoplasms, Androgen Independent’ OR ‘Prostatic Neoplasms, Androgen-Insensitive’ OR ‘Androgen-Insensitive Prostatic Neoplasm’ OR ‘Neoplasm, Androgen-Insensitive Prostatic’ OR ‘Neoplasms, Androgen-Insensitive Prostatic’ OR ‘Prostatic Neoplasm, Androgen-Insensitive’ OR ‘Prostatic Neoplasms, Androgen Insensitive’ OR ‘Prostatic Neoplasms, Androgen-Resistant’ OR ‘Androgen-Resistant Prostatic Neoplasm’ OR ‘Neoplasm, Androgen-Resistant Prostatic’ OR ‘Neoplasms, Androgen-Resistant Prostatic’ OR ‘Prostatic Neoplasm, Androgen-Resistant’ OR ‘Prostatic Neoplasms, Androgen Resistant’ OR ‘Androgen-Independent Prostatic Neoplasms’ OR ‘Androgen Independent Prostatic Neoplasms’ OR ‘Castration-Resistant Prostatic Neoplasms’ OR ‘Castration Resistant Prostatic Neoplasms’ OR ‘Prostatic Cancer, Castration-Resistant’ OR ‘Cancer, Castration-Resistant Prostatic’ OR ‘Cancers, Castration-Resistant Prostatic’ OR ‘Castration-Resistant Prostatic Cancers’ OR ‘Prostatic Cancer, Castration Resistant’ OR ‘Prostatic Cancers, Castration-Resistant’ OR ‘Androgen-Insensitive Prostatic Cancer’ OR ‘Androgen Insensitive Prostatic Cancer’ OR ‘Androgen-Resistant Prostatic Cancer’ OR ‘Androgen Resistant Prostatic Cancer’ OR ‘Prostatic Cancer, Hormone Refractory’ OR ‘Hormone Refractory Prostatic Cancer’ OR ‘Prostatic Cancer, Androgen-Independent’ OR ‘Androgen-Independent Prostatic Cancers’ OR ‘Cancer, Androgen-Independent Prostatic’ OR ‘Cancers, Androgen-Independent Prostatic’ OR ‘Prostatic Cancer, Androgen Independent’ OR ‘Prostatic Cancers, Androgen-Independent’ OR ‘Prostatic Cancer, Androgen-Insensitive’ OR ‘Androgen-Insensitive Prostatic Cancers’ OR ‘Cancer, Androgen-Insensitive Prostatic’ OR ‘Cancers, Androgen-Insensitive Prostatic’ OR ‘Prostatic Cancer, Androgen Insensitive’ OR ‘Prostatic Cancers, Androgen-Insensitive’ OR ‘Prostatic Cancer, Androgen-Resistant’ OR ‘Androgen-Resistant Prostatic Cancers’ OR ‘Cancer, Androgen-Resistant Prostatic’ OR ‘Cancers, Androgen-Resistant Prostatic’ OR ‘Prostatic Cancer, Androgen Resistant’ OR ‘Prostatic Cancers, Androgen-Resistant’ OR ‘Androgen-Independent Prostatic Cancer’ OR ‘Androgen Independent Prostatic Cancer’ OR ‘Castration-Resistant Prostatic Cancer’ OR ‘Castration Resistant Prostatic Cancer’ OR ‘androgen-independent PC’ OR ‘androgen-independent PCa’ OR ‘androgen-independent prostate cancer’ OR ‘androgen-independent prostatic cancer’ OR ‘androgen-insensitive PC’ OR ‘androgen-insensitive PCa’ OR ‘androgen-insensitive prostate cancer’ OR ‘androgen-insensitive prostatic cancer’ OR ‘castrate-refractory PC’ OR ‘castrate-refractory PCa’ OR ‘castrate-refractory prostate cancer’ OR ‘castrate-refractory prostatic cancer’ OR ‘castrate-resistant PC’ OR ‘castrate-resistant PCa’ OR ‘castrate-resistant prostate cancer’ OR ‘castration-refractory PC’ OR ‘castration-refractory PCa’ OR ‘castration-refractory prostate cancer’ OR ‘castration-refractory prostatic cancer’ OR ‘castration-resistant PC’ OR ‘castration-resistant PCa’ OR ‘castration-resistant prostatic cancer’ OR ‘castration-resistant prostatic neoplasms’ OR ‘CRPC (castration resistant prostate cancer)’ OR ‘hormone refractory prostate cancer’ OR ‘hormone-refractory PC’ OR ‘hormone-refractory PCa’ OR ‘hormone-refractory prostatic cancer’ OR ‘hormone-resistant PC’ OR ‘hormone-resistant PCa’ OR ‘hormone-resistant prostate cancer’ OR ‘hormone-resistant prostatic cancer’ OR ‘prostatic neoplasms, castration-resistant’):ti,ab,kw | 2806 |
| #3 | MeSH descriptor: [Androgen Antagonists] explode all trees | 1094 |
| #4 | (‘Antagonists, Androgen’ OR ‘Antiandrogens’ OR ‘Androgen Antagonist’ OR ‘Antagonist, Androgen’ OR ‘Antiandrogen’ OR ‘Anti-Androgen Effect’ OR ‘Anti Androgen Effect’ OR ‘Effect, Anti-Androgen’ OR ‘Antiandrogen Effect’ OR ‘Effect, Antiandrogen’ OR ‘Antiandrogen Effects’ OR ‘Effects, Antiandrogen’ OR ‘Anti-Androgen Effects’ OR ‘Anti Androgen Effects’ OR ‘Effects, Anti-Androgen’ OR ‘androgen antagonist’ OR ‘androgen antagonists’ OR ‘anti androgen’ OR ‘antiandrogen agent’ OR ‘antiandrogenic agent’ OR ‘antiandrogenic drug’ OR ‘antiandrogens’ OR ‘nonsteroidal anti androgen’ OR ‘nonsteroidal anti androgens’ OR ‘nonsteroidal anti-androgen’ OR ‘nonsteroidal anti-androgens’ OR ‘nonsteroidal antiandrogen’ OR ‘nonsteroidal antiandrogens’):ti,ab,kw | 2391 |
| #5 | #1 OR #2 | 2806 |
| #6 | #3 OR #4 | 2391 |
| #7 | #5 AND #6 | 430 |
| #8 | ('random*'):ti,ab,kw | 1173563 |
| #9 | #7 AND #8 | 339 |
