# Supplementary material for: The efficacy and adverse events of conventional and second-generation androgen receptor inhibitors for castration-resistant prostate cancer: A network meta-analysis
Source: Front Endocrinol (Lausanne). 2023 Feb 10;14:1131033. doi: 10.3389/fendo.2023.1131033 (PMC9950258; doi:10.3389/fendo.2023.1131033)
Supplement: Supplementary file 2 [file Table_2.docx]

Supplementary Material

The efficacy and adverse events of conventional and second-generation androgen receptor inhibitors for castration-resistant prostate cancer: a network meta-analysis

Xianlu Zhang^1^, Gejun Zhang^1^, Jianfeng Wang^1^, Jianbin Bi^1*^

*** Correspondence:** Jianbin Bi: jianbinbi@cmu.edu.cn

# Fig. S1 Network plots for included studies


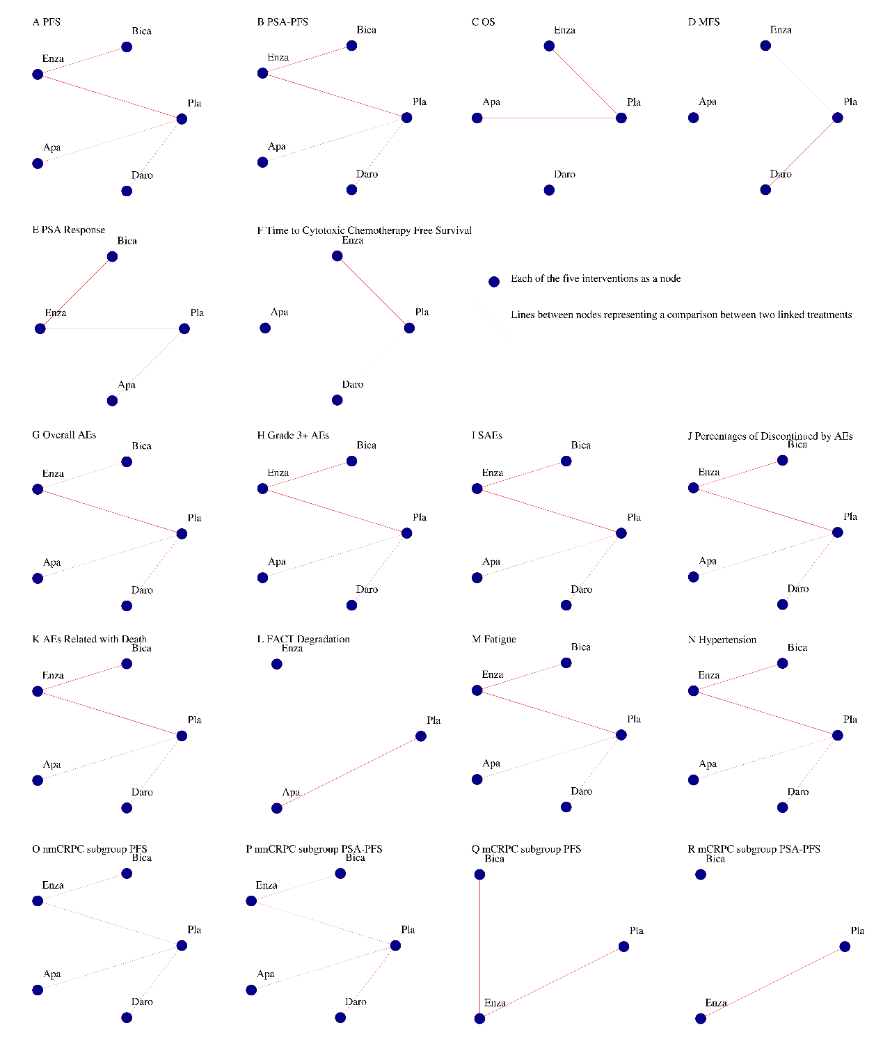


# Fig. S2 Quality assessments of the included studies


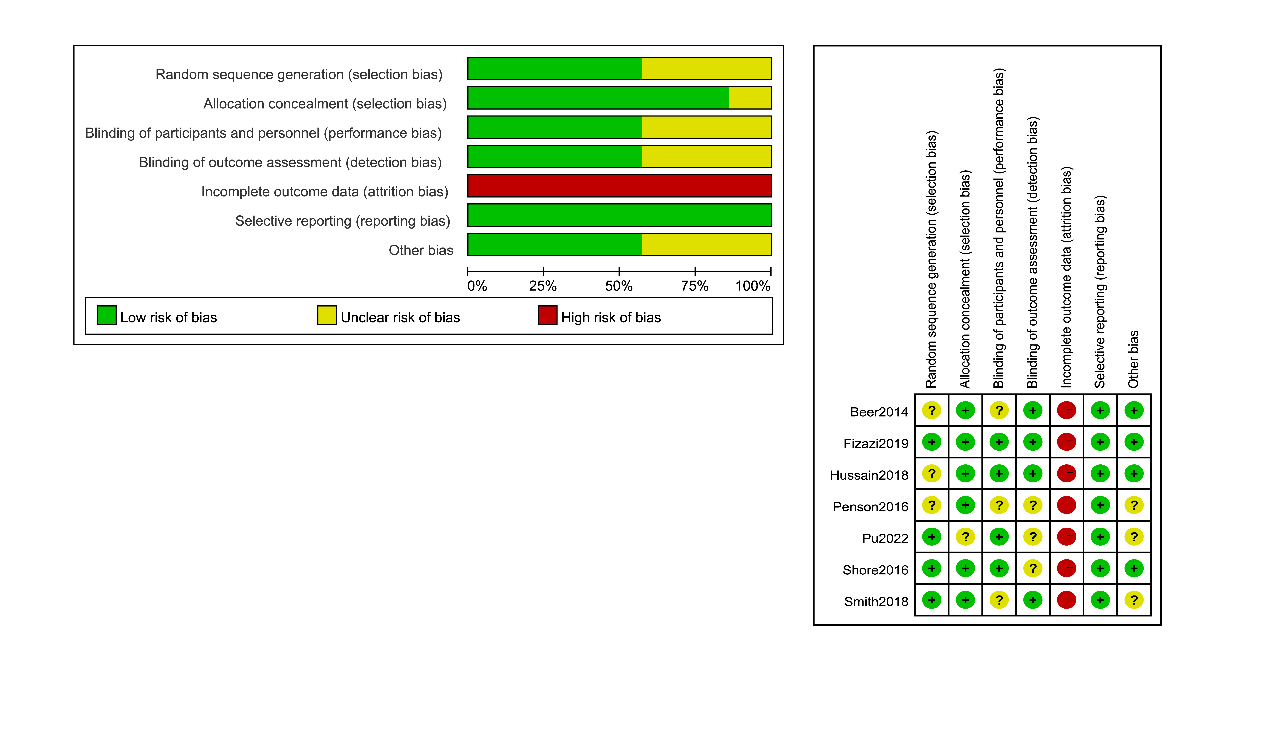


# Table S1 The league table of outcomes

| PFS |  |  |  |  |
| --- | --- | --- | --- | --- |
| PLA | 0.74 (0.58, 0.93) | 0.25 (0.22, 0.28) | 0.29 (0.24, 0.36) | 0.38 (0.32, 0.45) |
| 1.36 (1.07, 1.72) | BICA | 0.34 (0.28, 0.41) | 0.39 (0.29, 0.54) | 0.52 (0.39, 0.69) |
| 4.04 (3.54, 4.63) | 2.98 (2.46, 3.62) | ENZA | 1.17 (0.92, 1.5) | 1.54 (1.24, 1.91) |
| 3.44 (2.81, 4.22) | 2.54 (1.85, 3.47) | 0.85 (0.67, 1.09) | APA | 1.31 (1.01, 1.71) |
| 2.63 (2.21, 3.12) | 1.94 (1.45, 2.59) | 0.65 (0.52, 0.81) | 0.76 (0.58, 0.99) | DARO |
| PSA-PFS |  |  |  |  |
| PLA | 0.67 (0.52, 0.86) | 0.15 (0.14, 0.17) | 0.06 (0.05, 0.08) | 0.13 (0.11, 0.16) |
| 1.5 (1.16, 1.92) | BICA | 0.23 (0.18, 0.28) | 0.09 (0.06, 0.13) | 0.19 (0.14, 0.27) |
| 6.59 (5.87, 7.39) | 4.39 (3.52, 5.52) | ENZA | 0.4 (0.3, 0.51) | 0.86 (0.69, 1.07) |
| 16.65 (13.18, 21) | 11.13 (7.84, 15.68) | 2.53 (1.95, 3.28) | APA | 2.17 (1.61, 2.93) |
| 7.69 (6.38, 9.25) | 5.13 (3.76, 7.04) | 1.17 (0.94, 1.46) | 0.46 (0.34, 0.62) | DARO |
| OS |  |  |  |  |
| PLA | 0.71 (0.61, 0.82) | 0.7 (0.47, 1.04) | 0.71 (0.51, 1) |  |
| 1.42 (1.22, 1.64) | ENZA | 0.99 (0.65, 1.52) | 1.01 (0.7, 1.45) |  |
| 1.43 (0.96, 2.12) | 1.01 (0.66, 1.53) | APA | 1.01 (0.6, 1.71) |  |
| 1.41 (1, 1.98) | 0.99 (0.69, 1.43) | 0.99 (0.59, 1.66) | DARO |  |
| MFS |  |  |  |  |
| PLA | 0.29 (0.24, 0.35) | 0.28 (0.23, 0.34) | 0.41 (0.34, 0.5) |  |
| 3.44 (2.86, 4.16) | ENZA | 0.96 (0.73, 1.28) | 1.41 (1.08, 1.85) |  |
| 3.58 (2.9, 4.41) | 1.04 (0.78, 1.37) | APA | 1.47 (1.1, 1.95) |  |
| 2.44 (2, 2.95) | 0.71 (0.54, 0.93) | 0.68 (0.51, 0.91) | DARO |  |
| PSA Response Rate | | | | |
| PLA | 6.44 (4.74, 9.04) | 20.11 (15.69, 27.04) | 41.17 (21.86, 82.94) |  |
| 0.16 (0.11, 0.21) | ENZA | 3.11 (2.64, 3.73) | 6.35 (3.12, 13.53) |  |
| 0.05 (0.04, 0.06) | 0.32 (0.27, 0.38) | APA | 2.03 (1.02, 4.34) |  |
| 0.02 (0.01, 0.05) | 0.16 (0.07, 0.32) | 0.49 (0.23, 0.98) | DARO |  |
| Time to Cytotoxic Chemotherapy | | | | |
| PLA | 0.35 (0.3, 0.4) | 0.44 (0.29, 0.66) | 0.43 (0.31, 0.6) |  |
| 2.87 (2.5, 3.32) | ENZA | 1.26 (0.81, 1.95) | 1.23 (0.86, 1.77) |  |
| 2.27 (1.51, 3.44) | 0.79 (0.51, 1.23) | APA | 0.98 (0.58, 1.66) |  |
| 2.33 (1.67, 3.22) | 0.81 (0.57, 1.16) | 1.02 (0.6, 1.73) | DARO |  |
| Overall AEs |  |  |  |  |
| PLA | 1.01 (0.95, 1.08) | 1.05 (1.03, 1.07) | 1.04 (1.01, 1.07) | 1.08 (1.03, 1.14) |
| 0.99 (0.93, 1.06) | BICA | 1.04 (0.98, 1.11) | 1.02 (0.95, 1.11) | 1.07 (0.98, 1.17) |
| 0.95 (0.93, 0.97) | 0.96 (0.9, 1.02) | ENZA | 0.99 (0.95, 1.02) | 1.03 (0.97, 1.09) |
| 0.97 (0.93, 0.99) | 0.98 (0.9, 1.05) | 1.01 (0.98, 1.05) | APA | 1.04 (0.98, 1.11) |
| 0.92 (0.87, 0.97) | 0.93 (0.86, 1.02) | 0.97 (0.92, 1.03) | 0.96 (0.9, 1.02) | DARO |
| Geade3+ AEs |  |  |  |  |
| PLA | 1.16 (0.94, 1.42) | 1.17 (1.07, 1.29) | 1.32 (1.13, 1.55) | 1.24 (1, 1.57) |
| 0.86 (0.7, 1.07) | BICA | 1.01 (0.84, 1.22) | 1.14 (0.88, 1.48) | 1.08 (0.79, 1.46) |
| 0.85 (0.78, 0.94) | 0.99 (0.82, 1.18) | ENZA | 1.13 (0.94, 1.36) | 1.06 (0.83, 1.37) |
| 0.76 (0.64, 0.88) | 0.87 (0.67, 1.13) | 0.89 (0.73, 1.06) | APA | 0.94 (0.71, 1.24) |
| 0.8 (0.64, 1) | 0.93 (0.68, 1.27) | 0.94 (0.73, 1.2) | 1.06 (0.8, 1.4) | DARO |
| SAEs |  |  |  |  |
| PLA | 1 (0.78, 1.29) | 1.17 (1.04, 1.32) | 1.07 (0.87, 1.34) | 1.22 (1.01, 1.5) |
| 1 (0.77, 1.29) | BICA | 1.17 (0.93, 1.47) | 1.07 (0.77, 1.5) | 1.22 (0.89, 1.69) |
| 0.85 (0.76, 0.96) | 0.85 (0.68, 1.07) | ENZA | 0.92 (0.72, 1.18) | 1.04 (0.83, 1.32) |
| 0.93 (0.74, 1.15) | 0.93 (0.67, 1.29) | 1.09 (0.85, 1.39) | APA | 1.14 (0.85, 1.53) |
| 0.82 (0.67, 0.99) | 0.82 (0.59, 1.13) | 0.96 (0.76, 1.2) | 0.88 (0.66, 1.18) | DARO |
| Percentages of Discontinued by AEs | | | | |
| PLA | 0.86 (0.58, 1.28) | 1.05 (0.84, 1.33) | 1.51 (1.02, 2.32) | 1.03 (0.74, 1.46) |
| 1.16 (0.78, 1.72) | BICA | 1.23 (0.9, 1.68) | 1.76 (1.01, 3.13) | 1.2 (0.72, 2.01) |
| 0.95 (0.75, 1.19) | 0.82 (0.6, 1.12) | ENZA | 1.43 (0.91, 2.32) | 0.98 (0.65, 1.48) |
| 0.66 (0.43, 0.98) | 0.57 (0.32, 0.99) | 0.7 (0.43, 1.1) | APA | 0.68 (0.4, 1.16) |
| 0.97 (0.69, 1.36) | 0.83 (0.5, 1.4) | 1.03 (0.68, 1.54) | 1.47 (0.86, 2.52) | DARO |
| AEs Related with Death | | | | |
| PLA | 0.86 (0.34, 2.11) | 1.49 (1.03, 2.2) | 6.73 (1.1, 183.14) | 3.03 (0.38, 85.13) |
| 1.16 (0.47, 2.97) | BICA | 1.72 (0.77, 4.14) | 8.04 (1.02, 237.61) | 3.54 (0.37, 112.76) |
| 0.67 (0.45, 0.97) | 0.58 (0.24, 1.29) | ENZA | 4.52 (0.7, 122.88) | 2.03 (0.25, 57.59) |
| 0.15 (0.01, 0.91) | 0.12 (0, 0.98) | 0.22 (0.01, 1.42) | APA | 0.43 (0.01, 19.49) |
| 0.33 (0.01, 2.61) | 0.28 (0.01, 2.72) | 0.49 (0.02, 4.07) | 2.31 (0.05, 103.41) | DARO |
| FACT Degradation |  |  |  |  |
| PLA | 1.01 (0.74, 1.37) | 0.91 (0.79, 1.07) |  |  |
| 0.99 (0.73, 1.36) | ENZA | 0.91 (0.7, 1.19) |  |  |
| 1.09 (0.94, 1.27) | 1.1 (0.84, 1.44) | APA |  |  |
| Fatigue |  |  |  |  |
| PLA | 1.24 (0.96, 1.61) | 1.69 (1.5, 1.9) | 1.33 (1.07, 1.66) | 1.39 (1.02, 1.94) |
| 0.8 (0.62, 1.05) | BICA | 1.36 (1.08, 1.71) | 1.07 (0.76, 1.51) | 1.12 (0.75, 1.71) |
| 0.59 (0.53, 0.67) | 0.74 (0.58, 0.93) | ENZA | 0.79 (0.62, 1.01) | 0.83 (0.59, 1.18) |
| 0.75 (0.6, 0.94) | 0.94 (0.66, 1.31) | 1.27 (0.99, 1.63) | APA | 1.05 (0.72, 1.56) |
| 0.72 (0.52, 0.98) | 0.89 (0.58, 1.34) | 1.21 (0.85, 1.7) | 0.95 (0.64, 1.4) | DARO |
| Hypertension |  |  |  |  |
| PLA | 1.16 (0.94, 1.42) | 1.17 (1.07, 1.29) | 1.32 (1.13, 1.55) | 1.24 (1, 1.57) |
| 0.86 (0.7, 1.07) | BICA | 1.01 (0.84, 1.22) | 1.14 (0.88, 1.48) | 1.08 (0.79, 1.46) |
| 0.85 (0.78, 0.94) | 0.99 (0.82, 1.18) | ENZA | 1.13 (0.94, 1.36) | 1.06 (0.83, 1.37) |
| 0.76 (0.64, 0.88) | 0.87 (0.67, 1.13) | 0.89 (0.73, 1.06) | APA | 0.94 (0.71, 1.24) |
| 0.8 (0.64, 1) | 0.93 (0.68, 1.27) | 0.94 (0.73, 1.2) | 1.06 (0.8, 1.4) | DARO |
| nmCRPC PFS |  |  |  |  |
| PLA | 1.2 (0.68, 2.18) | 0.29 (0.24, 0.35) | 0.29 (0.24, 0.36) | 0.38 (0.32, 0.45) |
| 0.83 (0.46, 1.48) | BICA | 0.24 (0.14, 0.42) | 0.24 (0.13, 0.44) | 0.32 (0.17, 0.58) |
| 3.45 (2.85, 4.17) | 4.16 (2.41, 7.26) | ENZA | 1 (0.76, 1.32) | 1.31 (1.02, 1.69) |
| 3.45 (2.81, 4.22) | 4.16 (2.25, 7.76) | 1 (0.76, 1.32) | APA | 1.31 (1, 1.71) |
| 2.63 (2.22, 3.12) | 3.17 (1.73, 5.86) | 0.76 (0.59, 0.98) | 0.76 (0.59, 1) | DARO |
| nmCPRC PSA-PFS | | | | |
| PLA | 0.39 (0.2, 0.75) | 0.07 (0.06, 0.09) | 0.06 (0.05, 0.08) | 0.13 (0.11, 0.16) |
| 2.56 (1.34, 4.94) | BICA | 0.18 (0.1, 0.33) | 0.15 (0.08, 0.31) | 0.33 (0.17, 0.66) |
| 14.28 (11.31, 18.05) | 5.56 (3.03, 10.19) | ENZA | 0.86 (0.62, 1.19) | 1.86 (1.38, 2.5) |
| 16.68 (13.18, 21.02) | 6.5 (3.23, 12.96) | 1.17 (0.84, 1.62) | APA | 2.17 (1.61, 2.93) |
| 7.7 (6.37, 9.3) | 3 (1.51, 5.87) | 0.54 (0.4, 0.73) | 0.46 (0.34, 0.62) | DARO |
| mCRPC PFS |  |  |  |  |
| PLA | 0.59 (0.45, 0.78) | 0.21 (0.17, 0.25) |  |  |
| 1.68 (1.28, 2.24) | BICA | 0.35 (0.29, 0.44) |  |  |
| 4.75 (3.93, 5.75) | 2.82 (2.29, 3.48) | ENZA |  |  |
| mCRPC PSA-PFS |  |  |  |  |
| PLA | 0.82 (0.62, 1.09) | 0.19 (0.17, 0.22) |  |  |
| 1.22 (0.92, 1.62) | BICA | 0.24 (0.18, 0.31) |  |  |
| 5.16 (4.53, 5.9) | 4.23 (3.28, 5.41) | ENZA |  |  |

Numbers in the table are the Relative Risk value of different comparisons of interventions.

# Table S2 The rank probabilities

| PFS | | | | | |
| --- | --- | --- | --- | --- | --- |
| Intervention | RANK1 | RANK2 | RANK3 | RANK4 | RANK5 |
| PLA | 0.68715 | 0.2793 | 0.0316 | 0.00185 | 0.0001 |
| BICA | 0.25665 | 0.5755 | 0.12265 | 0.0382 | 0.007 |
| ENZA | 0.0003 | 0.0049 | 0.118 | 0.3719 | 0.5049 |
| APA | 0.0202 | 0.0475 | 0.2309 | 0.36905 | 0.33235 |
| DARO | 0.0357 | 0.0928 | 0.49685 | 0.219 | 0.15565 |
| PSA-PFS | | | | | |
| Intervention | RANK1 | RANK2 | RANK3 | RANK4 | RANK5 |
| PLA | 0.61948 | 0.3394 | 0.03696 | 0.00388 | 0.00028 |
| BICA | 0.33204 | 0.51316 | 0.108 | 0.03304 | 0.01376 |
| ENZA | 0.0012 | 0.02808 | 0.49716 | 0.3754 | 0.09816 |
| APA | 0.0148 | 0.03608 | 0.08888 | 0.20992 | 0.65032 |
| DARO | 0.03248 | 0.08328 | 0.269 | 0.37776 | 0.23748 |
| OS | | | | | |
| Intervention | RANK1 | RANK2 | RANK3 | RANK4 |  |
| PLA | 0.7862 | 0.1543 | 0.0184 | 0.0016 |  |
| ENZA | 0.0448 | 0.2498 | 0.2943 | 0.4021 |  |
| APA | 0.0854 | 0.3013 | 0.3128 | 0.3612 |  |
| DARO | 0.0836 | 0.2946 | 0.3745 | 0.2351 |  |
| MFS | | | | | |
| Intervention | RANK1 | RANK2 | RANK3 | RANK4 |  |
| PLA | 0.8267 | 0.1521 | 0.0203 | 0.0009 |  |
| ENZA | 0.0447 | 0.1866 | 0.37335 | 0.39535 |  |
| APA | 0.041 | 0.1655 | 0.3521 | 0.4414 |  |
| DARO | 0.0876 | 0.4958 | 0.25425 | 0.16235 |  |
| PSA response rate | | | | | |
| Intervention | RANK1 | RANK2 | RANK3 | RANK4 |  |
| PLA | 0.00035 | 0.00475 | 0.0614 | 0.9335 |  |
| ENZA | 0.03035 | 0.0918 | 0.8206 | 0.05725 |  |
| APA | 0.1943 | 0.7549 | 0.0499 | 0.0009 |  |
| DARO | 0.775 | 0.14855 | 0.0681 | 0.00835 |  |
| Time to Cytotoxic Chemotherapy | | | | | |
| Intervention | RANK1 | RANK2 | RANK3 | RANK4 |  |
| PLA | 0.8519 | 0.1299 | 0.0174 | 0.0008 |  |
| ENZA | 0.01205 | 0.12545 | 0.30915 | 0.55335 |  |
| APA | 0.0722 | 0.39075 | 0.31645 | 0.2206 |  |
| DARO | 0.06385 | 0.3539 | 0.357 | 0.22525 |  |
| Overall AEs | | | | | |
| Intervention | RANK1 | RANK2 | RANK3 | RANK4 | RANK5 |
| PLA | 0.00208 | 0.02372 | 0.12468 | 0.39316 | 0.45636 |
| BICA | 0.13764 | 0.15296 | 0.19896 | 0.21124 | 0.2992 |
| ENZA | 0.22556 | 0.432 | 0.2612 | 0.07144 | 0.0098 |
| APA | 0.14596 | 0.19316 | 0.25712 | 0.23288 | 0.17088 |
| DARO | 0.48876 | 0.19816 | 0.15804 | 0.09128 | 0.06376 |
| Geade3+ AEs | | | | | |
| Intervention | RANK1 | RANK2 | RANK3 | RANK4 | RANK5 |
| PLA | 0.00132 | 0.01708 | 0.08816 | 0.25308 | 0.64036 |
| BICA | 0.12772 | 0.20108 | 0.24788 | 0.2346 | 0.18872 |
| ENZA | 0.05136 | 0.22576 | 0.40804 | 0.27448 | 0.04036 |
| APA | 0.49176 | 0.27336 | 0.11472 | 0.08076 | 0.0394 |
| DARO | 0.32784 | 0.28272 | 0.1412 | 0.15708 | 0.09116 |
| SAEs | | | | | |
| Intervention | RANK1 | RANK2 | RANK3 | RANK4 | RANK5 |
| PLA | 0.01892 | 0.11752 | 0.28784 | 0.37444 | 0.20128 |
| BICA | 0.07084 | 0.11336 | 0.15076 | 0.1892 | 0.47584 |
| ENZA | 0.175 | 0.34908 | 0.27612 | 0.16632 | 0.03348 |
| APA | 0.2318 | 0.21716 | 0.16456 | 0.17752 | 0.20896 |
| DARO | 0.50344 | 0.20288 | 0.12072 | 0.09252 | 0.08044 |
| Percentages of Discontinued by AEs | | | | | |
| Intervention | RANK1 | RANK2 | RANK3 | RANK4 | RANK5 |
| PLA | 0.0184 | 0.18904 | 0.37228 | 0.30356 | 0.11672 |
| BICA | 0.04116 | 0.09584 | 0.1218 | 0.17732 | 0.56388 |
| ENZA | 0.07196 | 0.29616 | 0.2986 | 0.27808 | 0.0552 |
| APA | 0.73164 | 0.1302 | 0.06032 | 0.04376 | 0.03408 |
| DARO | 0.13684 | 0.28876 | 0.147 | 0.19728 | 0.23012 |
| AEs Related with Death | | | | | |
| Intervention | RANK1 | RANK2 | RANK3 | RANK4 | RANK5 |
| PLA | 0.00176 | 0.03832 | 0.18776 | 0.4242 | 0.34796 |
| BICA | 0.03212 | 0.1036 | 0.1884 | 0.24916 | 0.42672 |
| ENZA | 0.05992 | 0.28784 | 0.45656 | 0.17208 | 0.0236 |
| APA | 0.59108 | 0.24684 | 0.06828 | 0.0492 | 0.0446 |
| DARO | 0.31512 | 0.3234 | 0.099 | 0.10536 | 0.15712 |
| FACT Degradation | | | | | |
| Intervention | RANK1 | RANK2 | RANK3 |  |  |
| PLA | 0.467067 | 0.341867 | 0.191067 |  |  |
| ENZA | 0.4552 | 0.260867 | 0.283933 |  |  |
| APA | 0.077733 | 0.397267 | 0.525 |  |  |
| Fatigue | | | | | |
| Intervention | RANK1 | RANK2 | RANK3 | RANK4 | RANK5 |
| PLA | 0.00156 | 0.01848 | 0.11392 | 0.3592 | 0.50684 |
| BICA | 0.09976 | 0.27176 | 0.276 | 0.2052 | 0.14728 |
| ENZA | 0.53004 | 0.32832 | 0.11584 | 0.02308 | 0.00272 |
| APA | 0.16136 | 0.18148 | 0.25236 | 0.22136 | 0.18344 |
| DARO | 0.20728 | 0.19996 | 0.24188 | 0.19116 | 0.15972 |
| Hypertension | | | | | |
| Intervention | RANK1 | RANK2 | RANK3 | RANK4 | RANK5 |
| PLA | 0.00216 | 0.03248 | 0.18248 | 0.41584 | 0.36704 |
| BICA | 0.05604 | 0.34128 | 0.23132 | 0.1604 | 0.21096 |
| ENZA | 0.8012 | 0.14896 | 0.03884 | 0.00972 | 0.00128 |
| APA | 0.06664 | 0.22592 | 0.2918 | 0.21568 | 0.19996 |
| DARO | 0.07396 | 0.25136 | 0.25556 | 0.19836 | 0.22076 |
| nmCRPC PFS | | | | | |
| Intervention | RANK1 | RANK2 | RANK3 | RANK4 | RANK5 |
| PLA | 0.33528 | 0.53208 | 0.10872 | 0.02276 | 0.00116 |
| BICA | 0.57028 | 0.22868 | 0.10568 | 0.06736 | 0.028 |
| ENZA | 0.00584 | 0.06024 | 0.2032 | 0.34948 | 0.38124 |
| APA | 0.03308 | 0.07368 | 0.19168 | 0.3114 | 0.39016 |
| DARO | 0.05552 | 0.10532 | 0.39072 | 0.249 | 0.19944 |
| nmCRPC PPFS | | | | | |
| Intervention | RANK1 | RANK2 | RANK3 | RANK4 | RANK5 |
| PLA | 0.64696 | 0.2676 | 0.0722 | 0.01248 | 0.00076 |
| BICA | 0.2478 | 0.4162 | 0.16344 | 0.10836 | 0.0642 |
| ENZA | 0.00896 | 0.06256 | 0.20868 | 0.4028 | 0.317 |
| APA | 0.03096 | 0.08684 | 0.16404 | 0.26508 | 0.45308 |
| DARO | 0.06532 | 0.1668 | 0.39164 | 0.21128 | 0.16496 |
| mCRPC PFS | | | | | |
| Intervention | RANK1 | RANK2 | RANK3 |  |  |
| PLA | 0.724933 | 0.261867 | 0.0132 |  |  |
| BICA | 0.2734 | 0.6996 | 0.027 |  |  |
| ENZA | 0.001667 | 0.038533 | 0.9598 |  |  |
| mCRPC PPFS | | | | | |
| Intervention | RANK1 | RANK2 | RANK3 |  |  |
| PLA | 0.451133 | 0.524867 | 0.024 |  |  |
| BICA | 0.547733 | 0.435933 | 0.016333 |  |  |
| ENZA | 0.001133 | 0.0392 | 0.959667 |  |  |

The first line represents interventions, the first row represents probability rank. The statistics in the table represent the probability that the interventions are ranked in the corresponding row.
