# Supplementary material for: The efficacy and adverse events of conventional and second-generation androgen receptor inhibitors for castration-resistant prostate cancer: A network meta-analysis
Source: Front Endocrinol (Lausanne). 2023 Feb 10;14:1131033. doi: 10.3389/fendo.2023.1131033 (PMC9950258; doi:10.3389/fendo.2023.1131033)
Supplement: Supplementary file 3 [file Table_3.docx]

Supplementary Material

The efficacy and adverse events of conventional and second-generation androgen receptor inhibitors for castration-resistant prostate cancer: a network meta-analysis

Xianlu Zhang^1^, Gejun Zhang^1^, Jianfeng Wang^1^, Jianbin Bi^1*^

*** Correspondence:** Jianbin Bi: jianbinbi@cmu.edu.cn

# Original data

| PFS | | | | | | | |
| --- | --- | --- | --- | --- | --- | --- | --- |
| Study | treatment | n | HR | LCI | UCI | diff | std.err |
| ARAMIS 2019 | Daro | 955 | 0.38 | 0.32 | 0.45 | -0.96758 | 0.086971 |
| ARAMIS 2019 | Pla | 554 |  |  |  |  |  |
| PREVAIL 2014 | Enza | 872 | 0.19 | 0.15 | 0.23 | -1.66073 | 0.109042 |
| PREVAIL 2014 | Pla | 845 |  |  |  |  |  |
| Pu 2022 | Enza | 198 | 0.31 | 0.2 | 0.46 | -1.17118 | 0.212477 |
| Pu 2022 | Pla | 190 |  |  |  |  |  |
| SPARTAN 2018 | Apa | 806 | 0.29 | 0.24 | 0.36 | -1.23787 | 0.103435 |
| SPARTAN 2018 | Pla | 401 |  |  |  |  |  |
| STRIVE 2016 | Enza | 198 | 0.24 | 0.18 | 0.32 | -1.42712 | 0.146777 |
| STRIVE 2016 | Bica | 198 |  |  |  |  |  |
| TERRAIN 2016 | Enza | 184 | 0.44 | 0.34 | 0.57 | -0.82098 | 0.131809 |
| TERRAIN 2016 | Bica | 191 |  |  |  |  |  |
| TITAN 2019 | Apa | 525 | 0.48 | 0.39 | 0.6 | -0.73397 | 0.109894 |
| TITAN 2019 | Pla | 527 |  |  |  |  |  |
| PROSPER 2018 | Enza | 933 | 0.29 | 0.24 | 0.35 | -1.23787 | 0.096249 |
| PROSPER 2018 | Pla | 468 |  |  |  |  |  |
| PSA-PFS | | | | | | | |
| Study | treatment | n | HR | LCI | UCI | diff | std.err |
| ARAMIS 2019 | Daro | 955 | 0.13 | 0.11 | 0.16 | -2.04022 | 0.095585 |
| ARAMIS 2019 | Pla | 554 |  |  |  |  |  |
| PREVAIL 2014 | Enza | 872 | 0.17 | 0.15 | 0.2 | -1.77196 | 0.073388 |
| PREVAIL 2014 | Pla | 845 |  |  |  |  |  |
| PROSPER 2018 | Enza | 933 | 0.07 | 0.05 | 0.08 | -2.65926 | 0.119899 |
| PROSPER 2018 | Pla | 468 |  |  |  |  |  |
| Pu 2022 | Enza | 198 | 0.38 | 0.27 | 0.52 | -0.96758 | 0.167196 |
| Pu 2022 | Pla | 190 |  |  |  |  |  |
| SPARTAN 2018 | Apa | 806 | 0.06 | 0.05 | 0.08 | -2.81341 | 0.119899 |
| SPARTAN 2018 | Pla | 401 |  |  |  |  |  |
| STRIVE 2016 | Enza | 198 | 0.19 | 0.14 | 0.26 | -1.66073 | 0.157918 |
| STRIVE 2016 | Bica | 198 |  |  |  |  |  |
| TERRAIN 2016 | Enza | 184 | 0.28 | 0.2 | 0.39 | -1.27297 | 0.170365 |
| TERRAIN 2016 | Bica | 191 |  |  |  |  |  |
| TITAN 2019 | Apa | 525 | 0.26 | 0.21 | 0.32 | -1.34707 | 0.107452 |
| TITAN 2019 | Pla | 527 |  |  |  |  |  |
| OS | | | | | | | |
| Study | treatment | n | HR | LCI | UCI | diff | std.err |
| ARAMIS 2019 | Daro | 955 | 0.71 | 0.5 | 0.99 | -0.34249 | 0.174259 |
| ARAMIS 2019 | Pla | 554 |  |  |  |  |  |
| PREVAIL 2014 | Enza | 872 | 0.71 | 0.6 | 0.84 | -0.34249 | 0.085835 |
| PREVAIL 2014 | Pla | 845 |  |  |  |  |  |
| PROSPER 2018 | Enza | 933 | 0.8 | 0.58 | 1.09 | -0.22314 | 0.160945 |
| PROSPER 2018 | Pla | 468 |  |  |  |  |  |
| Pu 2022 | Enza | 198 | 0.33 | 0.16 | 0.67 | -1.10866 | 0.365333 |
| Pu 2022 | Pla | 190 |  |  |  |  |  |
| SPARTAN 2018 | Apa | 806 | 0.7 | 0.47 | 1.04 | -0.35667 | 0.202613 |
| SPARTAN 2018 | Pla | 401 |  |  |  |  |  |
| TITAN 2019 | Apa | 525 | 0.67 | 0.51 | 0.89 | -0.40048 | 0.142044 |
| TITAN 2019 | Pla | 527 |  |  |  |  |  |
| MFS | | | | | | | |
| Study | treatment | n | HR | LCI | UCI | diff | std.err |
| ARAMIS 2019 | Daro | 955 | 0.41 | 0.34 | 0.5 | -0.8916 | 0.098383 |
| ARAMIS 2019 | Pla | 554 |  |  |  |  |  |
| PROSPER 2018 | Enza | 933 | 0.29 | 0.24 | 0.35 | -1.23787 | 0.096249 |
| PROSPER 2018 | Pla | 468 |  |  |  |  |  |
| SPARTAN 2018 | Apa | 806 | 0.28 | 0.23 | 0.35 | -1.27297 | 0.107106 |
| SPARTAN 2018 | Pla | 401 |  |  |  |  |  |
| Time to cytotoxic chemotherapy free survival | | | | | | | |
| Study | treatment | n | HR | LCI | UCI | diff | std.err |
| ARAMIS 2019 | Daro | 955 | 0.43 | 0.31 | 0.6 | -0.84397 | 0.168459 |
| ARAMIS 2019 | Pla | 554 |  |  |  |  |  |
| PREVAIL 2014 | Enza | 872 | 0.35 | 0.3 | 0.4 | -1.04982 | 0.073388 |
| PREVAIL 2014 | Pla | 845 |  |  |  |  |  |
| Pu 2022 | Enza | 198 | 0.28 | 0.12 | 0.66 | -1.27297 | 0.434885 |
| Pu 2022 | Pla | 190 |  |  |  |  |  |
| SPARTAN 2018 | Apa | 806 | 0.44 | 0.29 | 0.66 | -0.82098 | 0.209785 |
| SPARTAN 2018 | Pla | 401 |  |  |  |  |  |
| TITAN 2019 | Apa | 525 | 0.39 | 0.27 | 0.56 | -0.94161 | 0.186101 |
| TITAN 2019 | Pla | 527 |  |  |  |  |  |
| FACT degradation | | | | | | | |
| Study | treatment | n | HR | LCI | UCI | diff | std.err |
| PROSPER 2018 | Enza | 933 | 0.92 | 0.79 | 1.08 | -0.08338 | 0.079766 |
| PROSPER 2018 | Pla | 468 |  |  |  |  |  |
| Pu 2022 | Enza | 198 | 0.73 | 0.27 | 1.99 | -0.31471 | 0.509558 |
| Pu 2022 | Pla | 190 |  |  |  |  |  |
| STRIVE 2016 | Enza | 198 | 0.91 | 0.7 | 1.19 | -0.09431 | 0.135364 |
| STRIVE 2016 | Bica | 198 |  |  |  |  |  |
| PSA response rate | | | |  |  |  |  |
| study | treatment | responders | sampleSize |  |  |  |  |
| PREVAIL 2014 | 3 | 666 | 854 |  |  |  |  |
| PREVAIL 2014 | 1 | 27 | 777 |  |  |  |  |
| PROSPER 2018 | 3 | 712 | 933 |  |  |  |  |
| PROSPER 2018 | 1 | 11 | 468 |  |  |  |  |
| Pu 2022 | 3 | 120 | 182 |  |  |  |  |
| Pu 2022 | 1 | 15 | 148 |  |  |  |  |
| SPARTAN 2018 | 4 | 723 | 806 |  |  |  |  |
| SPARTAN 2018 | 1 | 9 | 401 |  |  |  |  |
| STRIVE 2016 | 3 | 156 | 192 |  |  |  |  |
| STRIVE 2016 | 2 | 61 | 195 |  |  |  |  |
| TERRAIN 2016 | 3 | 151 | 184 |  |  |  |  |
| TERRAIN 2016 | 2 | 40 | 191 |  |  |  |  |
| Overall AEs | | | |  |  |  |  |
| study | treatment | responders | sampleSize |  |  |  |  |
| ARAMIS 2019 | 5 | 794 | 954 |  |  |  |  |
| ARAMIS 2019 | 1 | 426 | 554 |  |  |  |  |
| PREVAIL 2014 | 3 | 844 | 871 |  |  |  |  |
| PREVAIL 2014 | 1 | 787 | 844 |  |  |  |  |
| PROSPER 2018 | 3 | 808 | 930 |  |  |  |  |
| PROSPER 2018 | 1 | 360 | 465 |  |  |  |  |
| Pu 2022 | 3 | 167 | 198 |  |  |  |  |
| Pu 2022 | 1 | 153 | 190 |  |  |  |  |
| SPARTAN 2018 | 4 | 775 | 803 |  |  |  |  |
| SPARTAN 2018 | 1 | 371 | 398 |  |  |  |  |
| STRIVE 2016 | 3 | 183 | 197 |  |  |  |  |
| STRIVE 2016 | 2 | 177 | 198 |  |  |  |  |
| Grade 3+ AEs | | | |  |  |  |  |
| study | treatment | responders | sampleSize |  |  |  |  |
| ARAMIS 2019 | 5 | 188 | 954 |  |  |  |  |
| ARAMIS 2019 | 1 | 88 | 554 |  |  |  |  |
| PREVAIL 2014 | 3 | 374 | 871 |  |  |  |  |
| PREVAIL 2014 | 1 | 313 | 844 |  |  |  |  |
| PROSPER 2018 | 3 | 292 | 930 |  |  |  |  |
| PROSPER 2018 | 1 | 109 | 465 |  |  |  |  |
| Pu 2022 | 3 | 49 | 198 |  |  |  |  |
| Pu 2022 | 1 | 56 | 190 |  |  |  |  |
| SPARTAN 2018 | 4 | 362 | 803 |  |  |  |  |
| SPARTAN 2018 | 1 | 136 | 398 |  |  |  |  |
| STRIVE 2016 | 3 | 70 | 197 |  |  |  |  |
| STRIVE 2016 | 2 | 72 | 198 |  |  |  |  |
| TERRAIN 2016 | 3 | 73 | 183 |  |  |  |  |
| TERRAIN 2016 | 2 | 72 | 189 |  |  |  |  |
| SAE | | | |  |  |  |  |
| study | treatment | responders | sampleSize |  |  |  |  |
| ARAMIS 2019 | 5 | 234 | 954 |  |  |  |  |
| ARAMIS 2019 | 1 | 111 | 554 |  |  |  |  |
| PREVAIL 2014 | 3 | 279 | 871 |  |  |  |  |
| PREVAIL 2014 | 1 | 226 | 844 |  |  |  |  |
| PROSPER 2018 | 3 | 226 | 930 |  |  |  |  |
| PROSPER 2018 | 1 | 85 | 465 |  |  |  |  |
| Pu 2022 | 3 | 34 | 198 |  |  |  |  |
| Pu 2022 | 1 | 47 | 190 |  |  |  |  |
| SPARTAN 2018 | 4 | 199 | 803 |  |  |  |  |
| SPARTAN 2018 | 1 | 92 | 398 |  |  |  |  |
| STRIVE 2016 | 3 | 58 | 197 |  |  |  |  |
| STRIVE 2016 | 2 | 56 | 198 |  |  |  |  |
| TERRAIN 2016 | 3 | 57 | 183 |  |  |  |  |
| TERRAIN 2016 | 2 | 44 | 189 |  |  |  |  |
| Percentage of discontinued by AEs | | | |  |  |  |  |
| study | treatment | responders | sampleSize |  |  |  |  |
| ARAMIS 2019 | 5 | 85 | 954 |  |  |  |  |
| ARAMIS 2019 | 1 | 48 | 554 |  |  |  |  |
| PREVAIL 2014 | 3 | 49 | 871 |  |  |  |  |
| PREVAIL 2014 | 1 | 51 | 844 |  |  |  |  |
| PROSPER 2018 | 3 | 87 | 930 |  |  |  |  |
| PROSPER 2018 | 1 | 28 | 465 |  |  |  |  |
| Pu 2022 | 3 | 26 | 198 |  |  |  |  |
| Pu 2022 | 1 | 34 | 190 |  |  |  |  |
| SPARTAN 2018 | 4 | 85 | 803 |  |  |  |  |
| SPARTAN 2018 | 1 | 28 | 398 |  |  |  |  |
| STRIVE 2016 | 3 | 15 | 197 |  |  |  |  |
| STRIVE 2016 | 2 | 12 | 198 |  |  |  |  |
| TERRAIN 2016 | 3 | 52 | 183 |  |  |  |  |
| TERRAIN 2016 | 2 | 44 | 189 |  |  |  |  |
| AE-related mortality | | | |  |  |  |  |
| study | treatment | responders | sampleSize |  |  |  |  |
| ARAMIS 2019 | 5 | 4 | 954 |  |  |  |  |
| ARAMIS 2019 | 1 | 1 | 554 |  |  |  |  |
| PREVAIL 2014 | 3 | 37 | 871 |  |  |  |  |
| PREVAIL 2014 | 1 | 32 | 844 |  |  |  |  |
| PROSPER 2018 | 3 | 32 | 930 |  |  |  |  |
| PROSPER 2018 | 1 | 3 | 465 |  |  |  |  |
| Pu 2022 | 3 | 7 | 198 |  |  |  |  |
| Pu 2022 | 1 | 6 | 190 |  |  |  |  |
| SPARTAN 2018 | 4 | 10 | 803 |  |  |  |  |
| SPARTAN 2018 | 1 | 1 | 398 |  |  |  |  |
| STRIVE 2016 | 3 | 6 | 197 |  |  |  |  |
| STRIVE 2016 | 2 | 6 | 198 |  |  |  |  |
| TERRAIN 2016 | 3 | 9 | 183 |  |  |  |  |
| TERRAIN 2016 | 2 | 3 | 189 |  |  |  |  |
| Fatigue | | | |  |  |  |  |
| study | treatment | responders | sampleSize |  |  |  |  |
| ARAMIS 2019 | 5 | 115 | 954 |  |  |  |  |
| ARAMIS 2019 | 1 | 48 | 554 |  |  |  |  |
| PREVAIL 2014 | 3 | 310 | 871 |  |  |  |  |
| PREVAIL 2014 | 1 | 218 | 844 |  |  |  |  |
| PROSPER 2018 | 3 | 330 | 930 |  |  |  |  |
| PROSPER 2018 | 1 | 64 | 465 |  |  |  |  |
| Pu 2022 | 3 | 25 | 198 |  |  |  |  |
| Pu 2022 | 1 | 12 | 190 |  |  |  |  |
| SPARTAN 2018 | 4 | 224 | 803 |  |  |  |  |
| SPARTAN 2018 | 1 | 84 | 398 |  |  |  |  |
| STRIVE 2016 | 3 | 74 | 197 |  |  |  |  |
| STRIVE 2016 | 2 | 56 | 198 |  |  |  |  |
| TERRAIN 2016 | 3 | 49 | 183 |  |  |  |  |
| TERRAIN 2016 | 2 | 36 | 189 |  |  |  |  |
| Hypertension | | | |  |  |  |  |
| study | treatment | responders | sampleSize |  |  |  |  |
| ARAMIS 2019 | 5 | 63 | 954 |  |  |  |  |
| ARAMIS 2019 | 1 | 29 | 554 |  |  |  |  |
| PREVAIL 2014 | 3 | 117 | 871 |  |  |  |  |
| PREVAIL 2014 | 1 | 35 | 844 |  |  |  |  |
| PROSPER 2018 | 3 | 111 | 930 |  |  |  |  |
| PROSPER 2018 | 1 | 24 | 465 |  |  |  |  |
| Pu 2022 | 3 | 7 | 198 |  |  |  |  |
| Pu 2022 | 1 | 0 | 190 |  |  |  |  |
| SPARTAN 2018 | 4 | 199 | 803 |  |  |  |  |
| SPARTAN 2018 | 1 | 79 | 398 |  |  |  |  |
| STRIVE 2016 | 3 | 24 | 197 |  |  |  |  |
| STRIVE 2016 | 2 | 10 | 198 |  |  |  |  |
| TERRAIN 2016 | 3 | 13 | 183 |  |  |  |  |
| TERRAIN 2016 | 2 | 6 | 189 |  |  |  |  |

LCI: lower confidence interval; UCI: upper confidence interval; std.err: Standard error; HR: Hazard Ratio

# R code

install.packages("gemtc")

install.packages("rJava")

install.packages("xlsx")

library("gemtc")

library("rJava")

library("xlsx")

setwd("C:/Users/xianluzhang/Desktop/各种内分泌治疗药物网状meta/研究各个结局的数据/mCRPC/PPFS")

getwd()

#####PPFS#####

treatments <- read.xlsx("trtdes.xlsx", sheetIndex = 1, header = TRUE)

treatments

PPFS <- read.xlsx("PPFS.xlsx", sheetName = "Sheet2", header = TRUE)

PPFS

PPFS_nw <- mtc.network(data.re = PPFS, description = "Network of ARI", treatments = treatments)

setEPS()

postscript("PPFSnetwork.eps", width = 30, height = 15,fonts=c("serif"))

plot(PPFS_nw, use.description = TRUE,

vertex.label.cex = 1.5,

#ertex.size = PFS$sampleSize/30, #节点大小以"涉及总样本量/100"加权，此处倍数可自定义

vertex.shapes = "circle", #节点形状

vertex.label.color = "black", #标签颜色

vertex.label.dist = 3, #标签位置（距离）

vertex.label.degree = -pi/3, #标签位置（角度）

vertex.color = "darkblue", #节点颜色

dynamic.edge.width = TRUE, #连线粗细以涉及研究数加权

edge.color = "gray", #连线颜色

vertex.label.font = 1)

dev.off()

PPFS_mdl <- mtc.model(PPFS_nw, likelihood = "poisson", link = "log", type = "consistency", linearModel = "random", n.chain=3, dic = TRUE)

PPFS_result <- mtc.run(PPFS_mdl, n.adapt = 10000, n.iter = 50000, thin = 10)

summary(PPFS_result)

dev.new()

setEPS()

postscript("PPFS轨迹图与密度图.eps", width = 30, height = 15)

plot(PPFS_result)#轨迹图与密度图

dev.off()

dev.new()

setEPS()

postscript("PPFS收敛诊断图.eps", width = 30, height = 15)

gelman.plot(PPFS_result)#收敛诊断图

dev.off()

dev.new()

setEPS()

postscript("PPFS森林图.eps", width = 30, height = 15)

forest(relative.effect(PPFS_result , t1 = "3"), use.description = TRUE)#相对效应森林图

dev.off()

PPFS_tb <- round(exp(relative.effect.table(PPFS_result)),2)#联赛数据生成

PPFS_tb

write.xlsx(PPFS_tb, "PPFS_tb.xlsx")#联赛表生成

dev.new()

PPFS_rank <- rank.probability(PPFS_result, preferredDirection = 1)#概率排序图

setEPS()

postscript("PPFS概率排序图.eps", width = 30, height = 15)

plot(PPFS_rank)

dev.off()

print(PPFS_rank)

dev.new()

PPFS_sucrarank <- sucra(PPFS_rank)#概率sucra排序图

setEPS()

postscript("PPFS概率sucra排序图.eps", width = 30, height = 15)

plot(PPFS_sucrarank)

dev.off()

PFS_ns <- summary(mtc.nodesplit(PFS_nw, comparisons = mtc.nodesplit.comparisons(PFS_nw),

linearModel = "random", n.adapt = 10000, n.iter = 50000, thin = 10))

#####OAED#####

setwd("C:/Users/xianluzhang/Desktop/各种内分泌治疗药物网状meta/研究各个结局的数据/mCRPC/PRR")

getwd()

treatments <- read.xlsx("trtdes.xlsx", sheetIndex = 1, header = TRUE)

treatments

PRR <- read.xlsx("PRR.xlsx", sheetIndex = 1, header = TRUE)

PRR

PRR_nw <- mtc.network(data = PRR, description = "Network of ARI", treatments = treatments)

setEPS()

postscript("PRRnetwork.eps", width = 30, height = 15,fonts=c("serif"))

plot(PRR_nw, use.description = TRUE,

vertex.label.cex = 1.5,

#ertex.size = OADE$sampleSize/30, #节点大小以"涉及总样本量/100"加权，此处倍数可自定义

vertex.shapes = "circle", #节点形状

vertex.label.color = "black", #标签颜色

vertex.label.dist = 3, #标签位置（距离）

vertex.label.degree = -pi/3, #标签位置（角度）

vertex.color = "darkblue", #节点颜色

dynamic.edge.width = TRUE, #连线粗细以涉及研究数加权

edge.color = "gray", #连线颜色

vertex.label.font = 1)

dev.off()

PRR_mdl <- mtc.model(PRR_nw, likelihood = "binom", link = "log", type = "consistency", linearModel = "random", n.chain=3, dic = TRUE)

PRR_result <- mtc.run(PRR_mdl, n.adapt = 10000, n.iter = 50000, thin = 10)

summary(PRR_result)

dev.new()

setEPS()

postscript("PRR轨迹图与密度图.eps", width = 30, height = 15)

plot(PRR_result)#轨迹图与密度图

dev.off()

dev.new()

setEPS()

postscript("PRR收敛诊断图.eps", width = 30, height = 15)

gelman.plot(PRR_result)#收敛诊断图

dev.off()

dev.new()

setEPS()

postscript("PRR森林图.eps", width = 30, height = 15)

forest(relative.effect(PRR_result , t1 = "3"))#相对效应森林图

dev.off()

PRR_tb <- round(exp(relative.effect.table(PRR_result)),2)#联赛数据生成

PRR_tb

write.xlsx(PRR_tb, "PRR_tb.xlsx")#联赛表生成

dev.new()

PRR_rank <- rank.probability(PRR_result, preferredDirection = 1)#概率排序图

setEPS()

postscript("PRR概率排序图.eps", width = 30, height = 15)

plot(PRR_rank)

dev.off()

print(PRR_rank)

dev.new()

PRR_sucrarank <- sucra(PRR_rank)#概率sucra排序图

setEPS()

postscript("PRR概率sucra排序图.eps", width = 30, height = 15)

plot(PRR_sucrarank)

dev.off()
